# Characteristics of exceptionally good Doctors—A survey of public adults

**DOI:** 10.1016/j.heliyon.2023.e13115

**Published:** 2023-01-21

**Authors:** Christoph Schnelle, Mark A. Jones

**Affiliations:** Bond University, 14 University Drive, Robina, QLD, 4226, Australia

**Keywords:** Medical practice, Good doctors, Doctors' performance, Patients' opinion, Survey

## Abstract

**Background:**

Systematic reviews have found that doctors can have a substantial effect on patients’ physical health, beyond what can be explained by known factors. In a previous qualitative study, 13 medical doctors were interviewed on their experiences of exceptionally good doctors, and all had met at least one such doctor.

**Objective:**

To determine how common it is for exceptionally good doctors to be encountered by patients and what are the characteristics of exceptionally good doctors.

**Design:**

Mixed methods cross-sectional survey of 580 Amazon Mechanical Turk participants. Questions included doctor and participant demographics, and 34 Likert questions on characteristics of exceptionally good and average doctors. Free-text questions allowed participants to describe exceptional doctors, record their experience, and provide survey feedback. Stratified sampling ensured gender parity and 33% of participants aged ≥55 years. Analysis included descriptive statistics, statistical modelling of associations between Likert scale scores and patient demographics, and factor analysis.

**Results:**

Of 580 responses, 505 (86%) were included in the analysis. Factor analysis confirmed internal validity. Most respondents (86%) had met at least two exceptionally good doctors, of whom 55% were specialists. 58% of respondents regarded doctors as exceptional based on an overall impression with multiple reasons. Doctors were most commonly considered exceptional based on one or more of their personality, diagnostic, or intervention ability. Respondents who reported the doctors “willingly listened to them to the end” scored their doctors higher on 33 of 34 Likert questions, except for popularity. They also rated average doctors lower throughout.

**Conclusions:**

Exceptionally good doctors appear to be commonly encountered by the adult public. Listening to patients willingly to the end is a highly rated and influential characteristic, suggesting that listening could be targeted for quality improvement.

## Highlights

• 86% of members of the public remember at least one exceptionally good doctor.
• Such doctors are outstanding communicators, or diagnosticians, or interventionists.
• Exceptionally good doctors who willingly listen to patients to the end:
• Such doctors stand out even among exceptionally good doctors.
• They are considered more knowledgeable, caring, honest, with more integrity.

## 1 Introduction

Medical doctors are known to have a clustering effect in clinical trials [[1], [2], [3], [4]], i.e. patients of a particular doctor tend to have similar outcomes, which is likely due to confounding factors such as differences in patient demographics across practices, but could also be due to doctors having different levels of ability in treating patients.

To discern whether doctors' have varying ability in treating patients, the authors conducted a systematic review [[5], [6], [7]] screening over 10,000 studies and found that doctors have an effect on patients’ physical health, varying from the negligible to substantial, depending on the intervention and outcome measured. This effect persists after all known variables, such as doctor demographics and experience, hospital effects, patient demographics, and risk factors have been accounted for [8,9]. Some of the doctors had substantially better patient health outcomes, and the authors chose to label these doctors as “exceptionally good doctors”. However, there are few studies on exceptionally good doctors [[10], [11], [12]] or even good doctors [[13], [14], [15], [16], [17]] though a British Medical Journal (BMJ) 2002 special issue covered this subject [[18], [19], [20], [21], [22], [23], [24]]. and there are many opinion pieces [[25], [26], [27], [28], [29], [30], [31], [32]].

None of the studies that identified exceptionally good doctors provided recommendations for further research or published further details of such exceptionally good doctors [[33], [34], [35], [36], [37]]. In a recent qualitative study [38], 13 medical doctors stated their thoughts on what are the characteristics of exceptionally good doctors, with being both exceptionally skilled and very good at patient communication considered important. The doctor experiences and definitions from the qualitative study were used to design the present cross-sectional survey of the general public on their opinions on what makes an exceptionally good doctor, and their experiences of such doctors [39]. The survey objective was to determine how commonly exceptionally good doctors are encountered by patients, what are the characteristics of exceptionally good doctors according to patients, whether there are multiple types of exceptionally good doctors, if yes, whether patients evaluate different types of exceptionally good doctors more or less positively and whether patients evaluate exceptionally good doctors differently from average doctors.

## 2 Methods

The survey reporting follows the Strengthening the Reporting of Observational Studies in Epidemiology (STROBE) guidelines [40]. All details are presented in a previously published protocol [39].

### 2.1 Study DESIGN

This is an observational convergent design [41] cross-sectional survey including three qualitative, 19 quantitative (Table 1, Table 2, Table 3, Table 4, Table 5), and 34 5-point Likert questions (Table 6) where the 34 Likert questions were asked first to characterize the exceptionally good doctor nominated by the respondent and then the 34 Likert questions were asked of the average doctor (no specific doctor). The full survey is in Supplementary Appendix 7.

**Table 1:** Survey respondent demographics (N = 552).

Survey respondent demographics	n	%
**Demographics**		
Consented	587	100.0
Stopped at whether met EGD	35	6.0
Satisficers	35	6.0
Did not finish	12	2.0
Finished survey	505	86.0
**Sex (n = 505)**		
Male	237	46.9
Female	266	52.7
Non-binary	1	0.2
Prefer not to say	1	0.2
**Age (n = 505)**		
18-24	24	4.8
25-34	214	42.4
35-44	64	12.7
45-54	36	7.1
55-64	97	19.2
65+	70	13.9
**Education (n = 505)**		
No schooling completed	1	0.2
Grades 1 through 11	1	0.2
12th grade-no diploma	3	0.6
High school diploma	24	4.8
High school diploma equivalent	8	1.6
Some college (university)	21	4.2
1+ years of college, no degree	22	4.4
Associates degree	26	5.2
Bachelor's degree	277	54.9
Master's degree	96	19.0
Profess. degree (MD/ODS/DVM/LLB/JD}	8	1.6
Doctorate degree	18	3.6
**Country of Origin by IP address (n = 552)**		
United States of America	502	90.9
India	21	3.8
Brazil	7	1.3
Canada	6	1.1
Netherlands	5	0.9
United Kingdom	3	0.5
Italy	3	0.5
Unknown	3	0.5
Romania	1	0.2
Bangladesh	1	0.2
**Visits to any Doctor in previous 12 months? (n = 505)**		
Not at all	25	5.0
1–2 times	135	26.7
3–5 times	207	41.0
6–10 times	103	20.4
11–20 times	28	5.5
21–50 times	6	1.2
51 or more times	1	0.2
**Number of doctors met in life? (n = 505)**		
1-5	145	28.7
6-10	171	33.9
11-20	108	21.4
21-50	67	13.3
51-100	11	2.2
101 or more	3	0.6
**Number of exceptionally good doctors met in life? (n = 496)**		
1	69	13.9
2	185	37.3
3	138	27.8
4	51	10.3
5 or more	53	10.7
**Relationship to exceptionally good doctor? (n = 522)**		
I have been treated by one	469	89.9
I have met one	38	7.3
I know of one	10	1.9
None of the above	5	1.0

EGD: Exceptionally Good Doctor. Professional degrees: MD Medical Doctor, ODS Doctor of Optometry, DVM Doctor of Veterinary Medicine, LLB Bachelor of Law, JD Juris Doctor, Doctor of Law.

**Table 2:** Doctor information and evaluation (N = 517) – This table is best viewed while looking at the survey questions themselves to give context to the entries on this table.

Doctor Information		n	%
**Demographics**			
**Sex (n = 517)**			
Male		309	59.8
Female		208	40.2
**Age, estimated (n = 517)**			
Under 25 years		6	1.2
25-34		185	35.8
35-44		155	30.0
45-54		113	21.9
55-64		53	10.3
65+		5	1.0
**Type of doctor (n = 517)**			
Primary Care or GP		214	41.4
Hospital non-specialist		11	2.1
Likely Hospital non-specialist		5	1.0
Likely private non-specialist		3	0.6
**Subtotal**		233	45.1
Hospital specialist		174	33.7
Private practice specialist		51	9.9
Likely Hospital specialist		40	7.7
Likely private specialist		12	2.3
**Subtotal**		277	53.6
Other		7	1.4
**Specialty of doctor? (n = 414)**			
Cardiologist		63	15.2
All Surgeons (aggregate)		40	9.7
Emergency physician		35	8.5
Community child health		34	8.2
Psychiatrist		30	7.3
Dermatologist		23	5.6
Neurologist		13	3.1
Addiction medicine		12	2.9
Hospitalist/Internal Medicine		12	2.9
Surgeon, general		12	2.9
Not sure or not listed		12	2.9
Obstetrician and gynecologist		9	2.2
Oncologist		9	2.2
Gastroenterologist/hepatologist		8	1.9
Immunologist		8	1.9
Medical administrator		8	1.9
Public health physician		8	1.9
Pediatrician		7	1.7
Surgeon, orthopedic		7	1.7
Surgeon, cardio-thoracic		6	1.5
Urologist		6	1.5
Endocrinologist		5	1.2
Geriatrician		5	1.2
Gynecological oncologist		5	1.2
Intensive care physician		5	1.2
Nephrologist		5	1.2
Surgeon		5	1.2
Anesthetist		4	1.0
Neurosurgeon		4	1.0
Pain medicine physician		4	1.0
Ophthalmologist		3	0.7
Surgeon, pediatric		3	0.7
Immunologist and allergist		2	0.5
Surgeon, otolaryngologist		2	0.5
Infectious diseases physician		1	0.2
Respiratory and sleep medicine		1	0.2
Rheumatologist		1	0.2
Surgeon, plastic		1	0.2

GP General Practitioner, Primary Care Doctor.

**Table 3:** Patient-Doctor relationship (N = 513).

Patient-Doctor relationship	n	%
**How did you come across the doctor? (n = 954, multiple responses)**		
Recommended by a friend or family member or acquaintance	158	16.6
The doctor treated a family member	141	14.8
Recommended to me by a health care professional	128	13.4
The doctor is a close or extended family member	88	9.2
No recommendation, I found him or her myself	83	8.7
The doctor was my employer or superior	60	6.3
The doctor treated a colleague of mine	59	6.2
The doctor worked for me	54	5.7
The doctor was a colleague	46	4.8
The doctor was my teacher	44	4.6
Discovered via an internet search	43	4.5
The doctor was my student	32	3.4
Other	18	1.9
**How was doctor met? (n = 513)**		
General health check-up	248	48.3
Single health event	154	30.0
Multiple health events	61	11.9
Patient for a long time	31	6.0
Other	19	3.7
**Visits to exceptionally good doctor in previous 12 months (n = 513)**		
Not at all	69	13.5
1–2 times	144	28.1
3–5 times	187	36.5
6–10 times	84	16.4
11–20 times	24	4.7
21–50 times	4	0.8
51 or more times	1	0.2

**Table 4:** Patient opinions on Exceptionally Good Doctors.

Doctor Evaluation		n	%
What made you think this doctor is exceptionally good? (n = 1,910, 506 respondents)			
	It was an overall impression, there are multiple reasons	293	15.3
**Category:** Communication	I trust this doctor more than other doctors	224	11.7
Communication	I feel safe with this doctor, different to other doctors	194	10.2
	Because of this doctor I am *healthier* than I would otherwise be	179	9.4
Communication	The doctor listens to me willingly to the end	159	8.3
	This doctor definitely or probably saved my life	154	8.1
	I had an outstanding outcome, unexpectedly successful operation or recovery	144	7.5
Communication	I know the doctor will do whatever is needed to help me or has done so	142	7.4
	Because of this doctor I am *much healthier* than would otherwise be	114	6.0
Communication	The doctor allows me to make my own decisions	110	5.8
	The doctor treats financially poor patients at a discount or for free	83	4.4
Communication	Empowered me in my healing/treatment process much more than I thought possible	71	3.7
	The doctor is ready to extend guidelines and go off-label	43	2.3
**Could you state your reasons why you said this earlier (the doctor improved your health) (n = 925, 175 respondents)**			
Treatment	The doctor gave me a different treatment that worked very well	181	19.6
Treatment	The doctor changed my medication with a big beneficial effect	152	16.4
Diagnosis	I had a diagnosis that transformed my life for the better	119	12.9
Diagnosis	Difficult diagnosis because my symptoms were obscure/hidden/unusual	100	10.8
Diagnosis	I had a diagnosis that other doctors missed	91	9.8
Treatment	The doctor removed medication or other treatments and I was much better	85	9.2
Treatment	I had a dangerous or difficult operation and it went well	83	9.0
Treatment	I was not expected to recover a from a terminal illness but did	55	6.0
Treatment	I was not expected to recover from a non-terminal illness but did	47	5.1
	Other	12	1.3
**What is needed to be an exceptionally good doctor? (n = 513)**			
Outstanding in a single item		108	21.4
Outstanding in 2 or more areas		101	20.0
Surgeon one area, others multiple areas		65	12.9
Outstanding in everything		147	29.1
Above average in everything		80	15.8
Other		4	0.8

**Table 5:** Highest Likert ratings by respondents of exceptionally good doctors.

Likert ranking	n	%
How often was the characteristic below ranked as the most important characteristic among those characteristic where the respondent gave at least 4.5 out of 5, i.e. a very positive score in describing their exceptionally good doctor (n = 384 respondents with at least one Likert score≥4.5)		
Knowledgeable	45	11.7
Accurate diagnoser	35	9.1
Cares for patient	27	7.0
Good communicator	22	5.7
Sees patient as whole person	19	5.0
Very thorough in patient assessment	18	4.7
Honest	18	4.7
Understanding/empathy	18	4.7
Good at explaining	18	4.7
Very good observer	17	4.4
Patient trusts doctor	15	3.9
Confident	12	3.1
Listens, rarely interrupts	9	2.3
Open minded	9	2.3
Personable	9	2.3
Is caring	8	2.1
Connects on personal level	7	1.8
Always on time	7	1.8
Yes to patient's experience, knowledge	6	1.6
Humble	6	1.6
Great treatment room	6	1.6
Courageous in difficult decisions	5	1.3
Determined to get past obstacles	5	1.3
Popular	5	1.3
Good physical shape	5	1.3
Has patience	5	1.3
Good at following up	4	1.0
No fear of doctor, may be friend	4	1.0
Gives time needed	4	1.0
Has integrity	4	1.0
Organized	4	1.0
Avoids medical terminology	4	1.0
Good mental shape	3	0.8
Adaptable to the unexpected	1	0.3
**How often ranked as one of three most important (n = 384 respondents with at least one Likert score≥4.5, counting 1st place as 3, 2nd as 2, 3rd as 1, total 1837)**	**n**	**%**
Knowledgeable	166	9.0
Accurate diagnoser	155	8.4
Cares for patient	127	6.9
Good communicator	113	6.2
Very thorough in patient assessment	102	5.6
Honest	90	4.9
Patient trusts doctor	79	4.3
Sees patient as whole person	79	4.3
Understanding/empathy	76	4.1
Good at explaining	70	3.8
Very good observer	69	3.8
Open minded	65	3.5
Is caring	58	3.2
Confident	56	3.1
Gives time needed	48	2.6
Listens, rarely interrupts	42	2.3
Personable	39	2.1
Connects on personal level	36	2.0
Has patience	35	1.9
Courageous in difficult decisions	31	1.7
Humble	31	1.7
Determined to get past obstacles	28	1.5
Always on time	28	1.5
Yes to patient's experience, knowledge	27	1.5
Good at following up	23	1.3
Has integrity	23	1.3
Avoids medical terminology	23	1.3
Good physical shape	20	1.1
Adaptable to the unexpected	19	1.0
Great treatment room	19	1.0
Organized	17	0.9
Good mental shape	15	0.8
No fear of doctor, may be friend	14	0.8
Popular	14	0.8

**Table 6:** List of 34 Likert questions presented to respondents in random order for describing first the exceptionally good doctor they nominated and then the average doctor.

	Likert question ranging from 1 (completely disagree) to 5 (completely agree)
1	The doctor cares for patient
2	Acknowledges patient's experience and knowledge
3	Good at following things up or addressing items from prior consultation
4	Listens well, rarely or never interrupts
5	Connects with the patient on a personal level
6	The patient has no fear of the doctor and may see as a friend
7	The patient trusts the doctor
8	He/She sees patient as a whole person not just a collection of symptoms
9	The doctor is very thorough in the patient's assessment
10	The doctor is a very good observer
11	The doctor gives the patient the time needed
12	The doctor is confident
13	The doctor is courageous when making difficult decisions
14	The doctor is good at communicating
15	The doctor is adaptable, i.e. can respond to the unexpected
16	The doctor is honest
17	The doctor is humble
18	The doctor has integrity
19	The doctor is open minded
20	The doctor is organized
21	The doctor is personable
22	Determined to get past bureaucratic obstacles that affect treatment
23	The doctor is understanding and/or shows empathy
24	The doctor avoids using medical terminology I don't understand
25	The doctor is accurate in diagnosing the issue/problem
26	The doctor is good at explaining things
27	The doctor is knowledgeable
28	The doctor is popular (if you have seen the doctor with others)
29	The doctor is in good physical shape
30	The doctor is in good mental shape
31	The doctor is in an especially harmonious or cared for treatment room
32	The doctor is always on time
33	The doctor has patience
34	The doctor is caring

Participants were recruited through Amazon Mechanical Turk (MTurk) [42]. The MTurk worker population is a suitable proxy for the general population [[43], [44], [45]] and has been used extensively by social scientists [46], allowing stratification by gender and age. MTurk workers aged 55 and older and female MTurk workers were oversampled to get to a 50/50 gender split and to have 1/3 of respondents aged over 55. Further details are provided in the protocol paper [39]. Otherwise there were no further exclusions or inclusions – any MTurk worker could participate.

Initially all questions were derived from a qualitative study that interviewed 13 medical doctors on their experiences of exceptionally good doctors [38]. The authors conducted a pilot study with 210 respondents and employed a survey specialist with extensive consumer survey experience to improve the quality of the questions. The pilot study showed that respondents understood the term ‘exceptionally good doctors’ and factor analysis showed that this term was distinct from the term ‘doctor’.

The authors investigated alternatives to the term ‘doctor’, but such attempts were confusing and discouraging for respondents and were not used in the survey. The consent form and questions 5, 8, and 27 clarified that a doctor is a physician by using the term “doctor (physician)”.

Ethical approval (#CS03416) was granted by the Bond University Human Research Ethics Committee on April 27, 2022.

### 2.2 Survey sample

Adult MTurk workers were recruited as participants. The sample size was identified based on the results of a pilot study of 210 participants showing 400–450 participants were required to reduce the Likert question margin of error to ∼4%. A sample of 580 ensured 500 completed and valid responses.

### 2.3 Data collection

Demographic information collected included the respondents’ age in decades; gender; education level; and previous 12 months count of doctor visits. Their IP address (Internet Protocol address) identified their country. Additional questions included the number of exceptionally good doctors and total number of doctors the participants had previously encountered.

The respondents provided details on an exceptionally good doctor, including their estimated age, gender, specialty, and the reason why they nominated that doctor.

The participants were also asked 34 Likert questions, each listing a characteristic, derived from the previous qualitative study [38] – rating both the exceptionally good doctor and the average doctor on this characteristic using a scale from 1.0 (completely disagree) to 5.0 (completely agree) (Table 6). All respondents were asked all 34 Likert questions for both types of doctors rather than random allocation to either Likert questions type to allow within person comparison of exceptional and average doctors.

A subsequent question displayed the subset of Likert questions, if any, where the respondent rated a characteristic of an exceptionally good doctor as 4.5 out of 5 or higher and asked the respondent to nominate the top three of the listed characteristics in order.

Three free-text questions were asked: one at the beginning to nominate 3–5 characteristics of exceptionally good doctors; another mid-way through the survey to optionally write about their experience of the exceptionally good doctor in their own words, and one at the end of the survey to provide feedback.

Bias due to question order was minimized by, where possible, randomizing the order of multi-item questions such as the Likert questions. All quantitative questions were mandatory. Respondents who provided either logically impossible answers or highly uniform answers, i.e. satisficers [47], were excluded.

### 2.4 Statistical analysis

The analysis includes descriptive statistics on the respondents' and doctors' demographics, how the respondents met their nominated doctor, and why they considered that doctor exceptional. The results from the 34 Likert questions for exceptionally good doctors and the average doctors are shown in graphic form as kernel density plots (a smoothed form of histogram) [48]. Factor analysis assessed internal validation of the Likert questions. A linear regression each was run for the mean of the 34 Likert scores for the exceptionally good and the average doctor as factor analysis showed that there were only two factors with Eigenvalues above 1, one for exceptionally good and one for average doctors. Linear regression models were also used to explore the explanatory variables association with the individual Likert scores to identify Likert questions whose associations differed from the other Likert questions. T-values with absolute values ≥ 2.5 (p ≤ 0.01) were used to confirm evidence of association. As answers to Likert questions were not always normally distributed, we also conducted non-parametric and ordered logistic regression. We compared regression results between respondents who were patients of their nominated doctor and those who knew the doctor in other ways. In addition, we compared regression results for those who had an outstanding health event and those who didn't.

## 3 RESULTS

### 3.1 Factor analysis

Each Likert question is substantially correlated with the other 33 as shown in the factor analysis in supplementary appendix 6, i.e. all 34 measure a similar quality. Factor analysis identified two substantial factors with one constituting being a doctor and the second being an exceptionally good doctor. After varimax rotation both factors were near equal in size with Eigenvalues of 23.1 and 21.1 and all other Eigenvalues 0.78 or smaller. Therefore this survey measures two substantial separate factors only, one on which all Likert questions about exceptionally good doctors load and one on which all for average doctors load. The only question with a negative loading after rotation is “The doctor listens to me willingly to the end” which is negative for the average doctor, implying that listened-to respondents give average doctors lower Likert scores than other respondents.

### 3.2 Respondents’ and reported doctors’ demographics

Respondents' demographics are presented in Table 1. Of 587 respondents, 505 (86%) completed and provided valid answers. Thirty-five respondents (6%) did not know any exceptionally good doctors. Another 35 respondents were satisficers [49], and 12 respondents did not finish the survey. 53% of respondents were female, 42% were 25–34 years old, 33% were aged over 55 years, 55% had a bachelor's degree, 19% had a masters' degree, and 91% were from the US. 86% of respondents had met at least two exceptionally good doctors in their life.

Table 2 provides details on the exceptional doctors with 55% being specialists, 15% cardiologists, 10% surgeons, and 9% emergency physicians. 37% of doctors had an estimated age below 35 years, with 11% aged over 55 years. Most respondents (87%) had visited the exceptionally good doctor in the previous 12 months.

### 3.3 Respondents’ perspectives on exceptionally good doctors

**Fig. 1:**
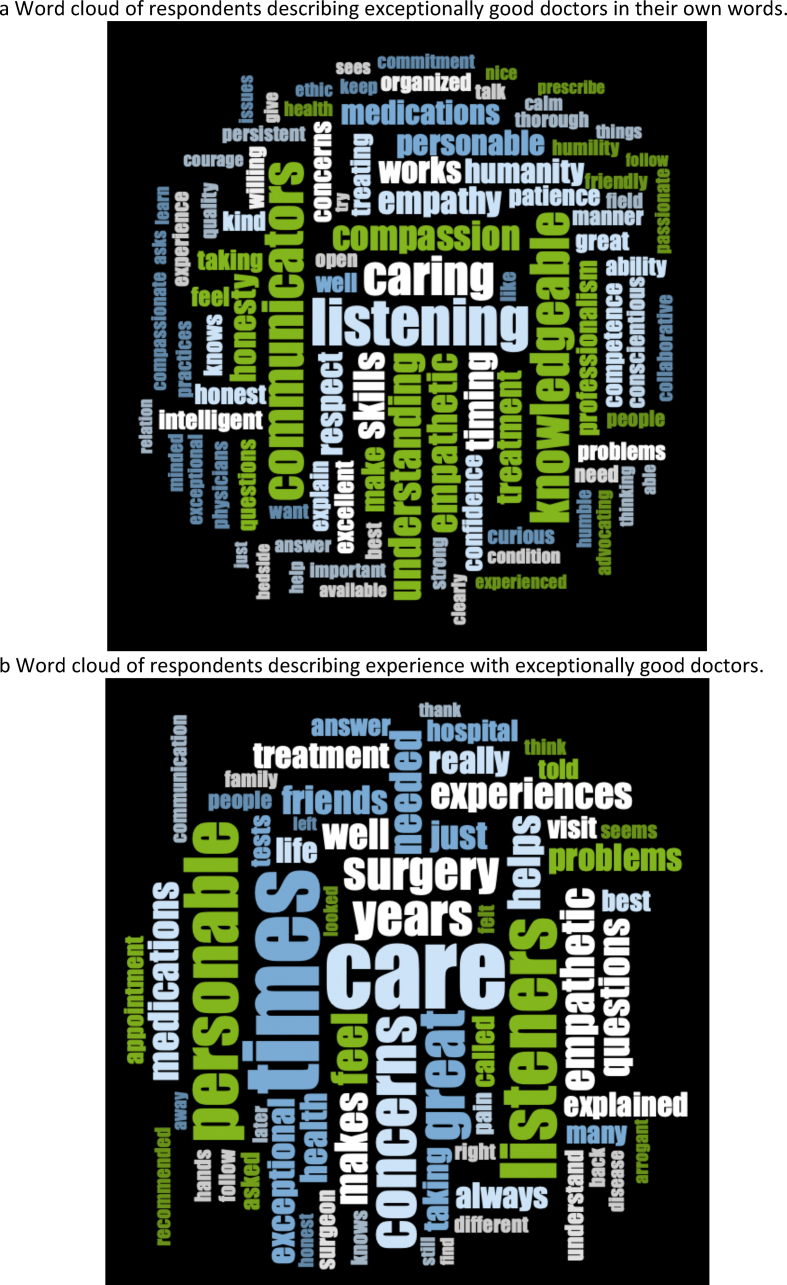
Wordclouds of free-text qualitative questions.

No consensus was shown in how many items a doctor had to fulfill to be considered exceptional. Qualities of exceptionally good doctors nominated by respondents are shown in Fig. 1a as a word cloud. Approximately 150 participants quoted verbatim from the highest-ranked google results on exceptionally good doctors and were excluded from the word cloud analysis [50,51]. Participants’ experiences with an exceptionally good doctor are summarized in a word cloud (Fig. 1b) and shown as raw data in Supplementary Appendix 2. A total of 468 respondents provided a response and 388 responses included 5 to 673 words.

The Likert question results showed that average doctors were rated with mean scores of 3.5–3.9 out of 5, and exceptionally good doctors at mean scores of 4.0–4.3. Exceptionally good doctors were nominated for three broad reasons: They were exceptional diagnosticians, exceptionally successful with interventions, or exceptionally good at relating to the patient. The respondents gave similar Likert scores to groups of exceptional doctors based on each of these three categories (Table 4, Doctor Evaluations). The exception is listening as outlined below.

The survey respondents were asked to select and rank the three most important questions among the Likert questions they scored 4.5 to 5 (the maximum score). A total of 387 respondents (77%) provided at least one score of 4.5 or higher; with 45 top ranks given to the doctor being knowledgeable, 35 for being accurate at diagnosing, and 22 for communication (Table 5, Likert ranking).

Results from the linear regression analysis of the mean Likert scores for each respondent showed that respondents aged 55 or older provided higher Likert scores for the exceptionally good doctors (t-value of 4.9, p < 0.001). The 159 respondents who reported “the doctor willingly listens to me to the end” scored their exceptionally good doctor higher than the other respondents (t = 6.9, p < 0.001) but also scored the average doctor more negatively than their peers (t = −3.3, p = 0.001). Female respondents scored average doctors higher than male respondents (t = 2.3, p = 0.02) and respondents who were patients of the exceptionally good doctor for a long time scored both types of doctors more highly (t = 2.1 and 2.4, p = 0.03 and 0.02). (Supplementary document, appendices 4 and 5). There was no difference in scores between the 334 respondents who were patients and the 218 respondents who knew the doctor in other ways, nor between the 362 respondents who had an outstanding health event and the 190 respondents who didn't. (Not listed).

Appendix 5 shows a summary of the 34 individual regressions for exceptionally good and average doctors to show variations in outcomes for some independent variables. For example t-values for exceptionally good doctor Likert questions for “The doctor willingly listens to the end” are above 2.5 (p = 0.01 or smaller) for 31 of the 34 Likert questions but not for the Likert question “The doctor is popular”.

**Fig. 2:**
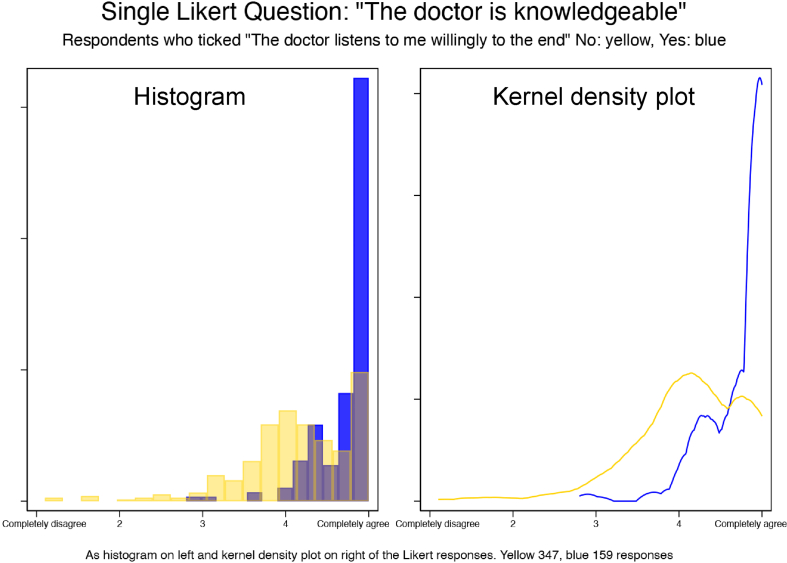
Histogram and kernel density plot of Likert question “The doctor is knowledgeable” by whether the doctors listens to the client.

Fig. 2 shows the distributions of answers to the Likert question “The (exceptionally good) doctor is knowledgeable” as histograms and kernel density plots stratified by whether the respondents affirmed that “The doctor listens to me willingly to the end” – blue for “Yes”, yellow for “No” [48].

**Fig. 3:**
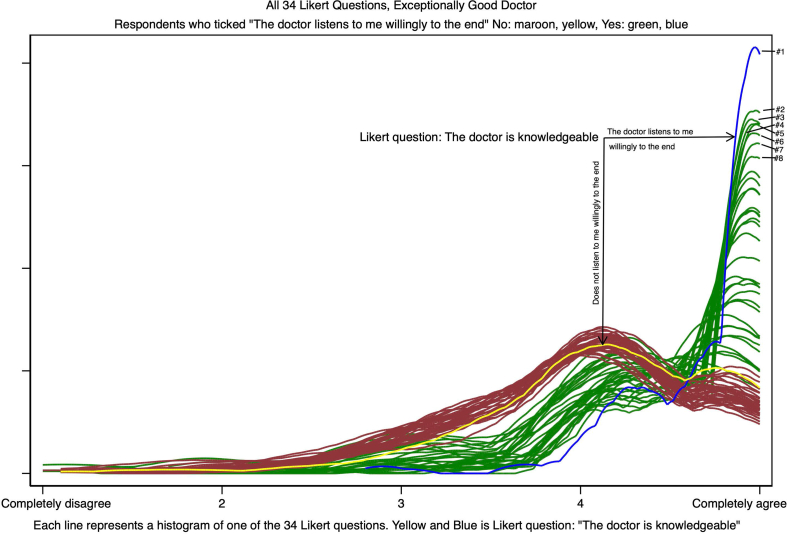
Kernel density plots of all 34 Likert questions. Respondents whose doctor listens consider the doctor much more knowledgeable (blue line) than respondents whose doctor does not do that (yellow line).

Fig. 3 shows all 34 Likert questions with the question shown in Fig. 2 highlighted. Fig. 3 illustrates where respondents, who were listened to by the doctor to the end, gave higher Likert scores than other respondents with the largest differences for.

1. The doctor is knowledgeable (blue line)
2. The doctor is caring (top green line)
3. The doctor is honest (2nd top green line etc.)
4. The doctor is good at communicating
5. The doctor cares for the patient
6. The doctor is understanding and/or shows empathy
7. The doctor has patience
8. The doctor has integrity

Supplementary Appendix 1 shows 18 descriptive graphs of items in Table 1, Table 2, Table 3, Table 4, Table 5. The qualitative responses are included in the Supplementary Appendix 2. Survey feedback obtained from 221 respondents is shown in Supplementary Appendix 3. Supplementary Appendix 4 shows the results from the regression analyses of the mean Likert scores per person. Supplementary Appendix 5 shows summary results from each of the 34 individual models for the exceptionally good doctors and also shows multiple subgroup analyses as similarly implemented in another paper on good doctors [52].

## 4 Discussion

Of the 580 respondents to our survey of the general adult public, 86% could recall meeting an exceptionally good doctor and describe such a doctor in detail. This result suggests a substantial number of such doctors exist, a finding consistent with a recent qualitative study in which 13 medical doctors were able to recall at least one exceptionally good doctor [38]. Hence, the dearth of research on such doctors or even good doctors is surprising [[13], [14], [15], [16], [17],^53^,^54^].

The survey respondents nominated doctors as being exceptionally good for at least one of three overarching reasons: for exceptional communication with the patient, an exceptional diagnostic, or an exceptionally successful intervention. The respondents gave similar scores to groups of exceptional doctors based on these three characteristics. The respondents therefore echoed medical doctors who stated that a doctor can be an exceptionally good doctor for a heterogenous set of reasons.

Of the variables assessed for association with the Likert scales ratings, three showed consistent positive or negative associations with exceptionally good and average doctors: Female respondents scored all doctors higher than their male counterparts; those aged 55 or higher scored exceptionally good doctors higher but average doctors lower than the younger respondents; and the 154 respondents who, responding to an item in question 17 of the survey, reported the doctor willingly listens to them to the end gave higher scores to the exceptionally good doctor and lower scores to the average doctor.

These 154 listened-to respondents considered their doctor to be particularly knowledgeable, caring, honest, and with integrity in addition to the expected qualities of being understanding, patient, and good at communicating. The quality of listening was associated with a host of seemingly unrelated positive associations for the patients. Patients being more critical of average doctors after meeting a doctor who listens could provide motivation for average doctors to undermine their exceptional colleagues. It is not a surprise that patients want their doctor to listen but there is no published research that shows quantitatively how much more positively listened-to patients rate their doctor. These participants considered the exceptionally good doctor to be substantially more knowledgeable and honest in addition to being better communicators and were substantially less positive about average doctors. These findings need replication but could potentially be a fruitful avenue for further research; addressing questions such as why doctors who listen to the end are considered more knowledgeable and honest, and why are their patients more critical of average doctors?

A medical specialist, in the same qualitative study described the process of listening as:

“Every patient, every person, every being is different. Every person has a different reading. So how can you be generalized into a sample or whatever, a random whatever. So that makes exceptional physicians more humble because you can't let go - be complacent. I got books everywhere. … But when I'm with a patient, I'm totally dedicated to listening - by listening I don't know - something comes up, an impress is given, the whole package of treatment comes through - more and more than ever before And that's what [three famous and exceptionally good doctors] did all the time”.

These statements suggest ‘listening’ could lead to more accurate diagnoses and appropriate treatments, supporting the 154 survey respondents' impression.

Currently, there is no definition of what is an exceptionally good doctor, and no characteristic in the survey was nominated as most important by more than 12% of the respondents. This suggests there are multiple ways to be an exceptional doctor. Doctors with exceptional communication qualities, excellent diagnostic abilities, or outstanding treatment success were equally valued by the respondents and the respondents had no consensus on how many qualities are needed to be exceptional. For our previous systematic reviews, we operationally defined an exceptionally good doctor as one who has exceptionally good patient physical health outcomes [[5], [6], [7]]. Conversely, our survey respondents took a much broader view on their opinions and experiences of exceptionally good doctors.

This survey has some limitations that need to be acknowledged. First, it was subject to potential non-response bias, as it was unable to know how many MTurk workers accessed the survey but chose not to participate. Nevertheless, response rates are less important than response representativeness [55], which was ensured as our sample of respondents are of gender and age distribution similar to that of the general population of adults. Second, the respondents were English speakers, predominantly from the US. Thus, it is uncertain whether the findings can be generalized to other regions, particularly developing nations. Third, in the US the percentage of health care costs covered by private health insurance (28%) and out of pocket expenses (10%) is higher than in other countries and doctors in the US system may differ from doctors in countries where public health systems pay more than 49% of health care costs [56]. In addition, due to heterogeneity regarding patient demographics, types of interventions, and types of outcomes relevant to different medical conditions, there may be differing criteria on what makes an exceptionally good doctor for different medical/surgical specialties and doctors and patients may differ in their perceptions of what makes an exceptionally good doctor as their perceptions differ in areas such as acute pain [57].

Despite the limitations, this survey of adult public provides an insightful view of exceptionally good doctors, who appear to be commonly encountered by the general adult public. They tend to be exceptional communicators, diagnosticians, or interventionists. The highest ratings for exceptional doctors are given by patients whose doctors listen to them willingly to the end. The ability to attentively listen makes an exceptionally good doctor stand out among their peers but its lack then also makes average doctors appear worse. Targeting listening skills for quality improvement could improve patient perceptions of doctors and potentially lead to better patient outcomes and higher doctor satisfaction.

## Author contribution statement

Christoph Schnelle: Conceived and designed the experiments; Performed the experiments; Analyzed and interpreted the data; Contributed reagents, materials, analysis tools or data; Wrote the paper.

Mark Jones: Conceived and designed the experiments; Analyzed and interpreted the data; Wrote the paper.

## Funding statement

This review has been funded by the first author as part of his PhD studies. No external funding was received.

## Data availability statement

Data will be made available on request.

## Declaration of interest's statement

The authors declare that they have no known competing financial interests or personal relationships that could have appeared to influence the work reported in this paper.

## Additional information

Supplementary content related to this article has been published online at [URL].
