# Supplementary material for: Characteristics of exceptionally good Doctors—A survey of public adults
Source: Heliyon. 2023 Jan 21;9(2):e13115. doi: 10.1016/j.heliyon.2023.e13115 (PMC9883187; doi:10.1016/j.heliyon.2023.e13115)
Supplement: Multimedia component 1 [file mmc1.docx]

# Supplementary document

# Appendix 1 – further graphs


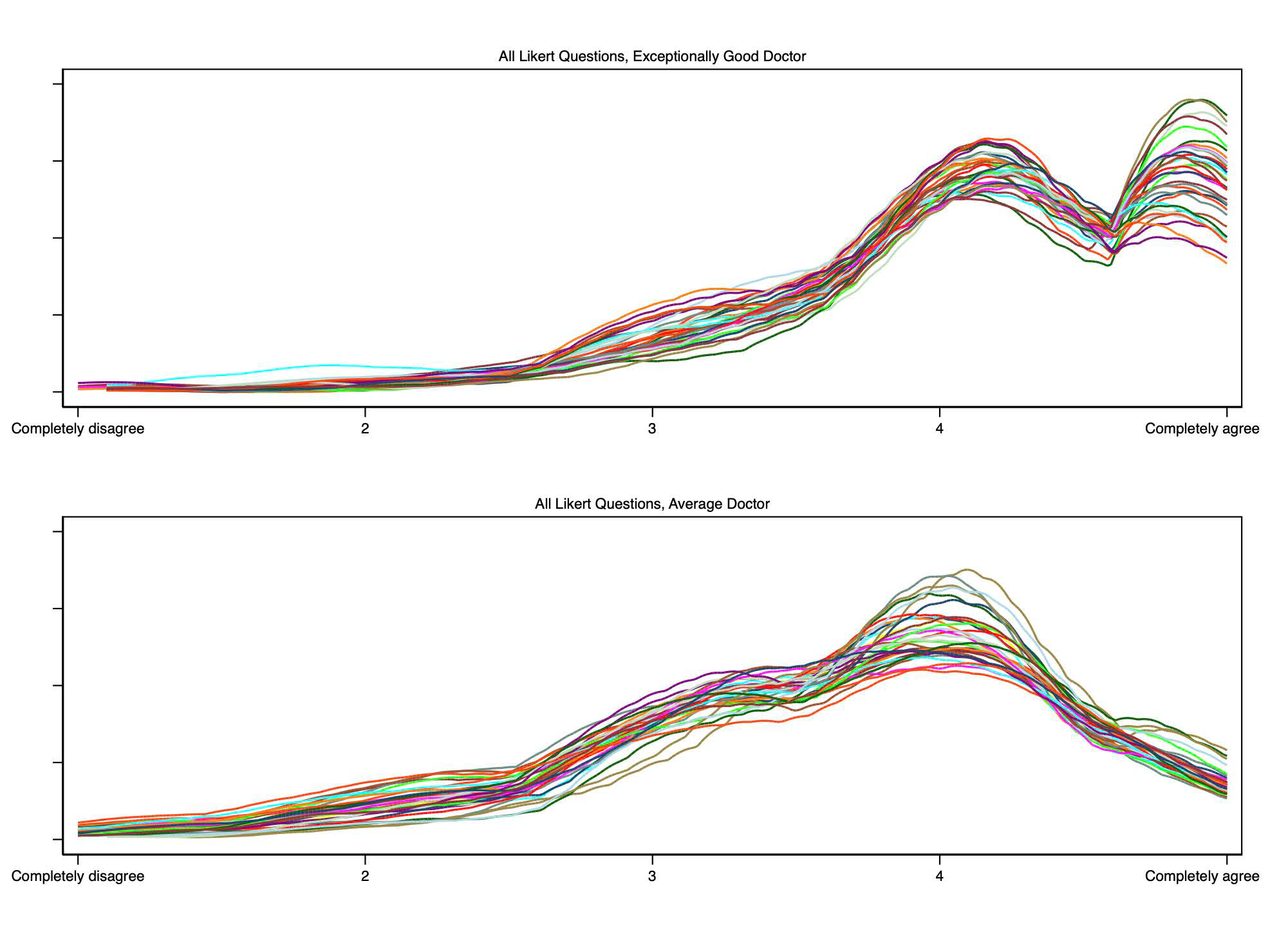


| 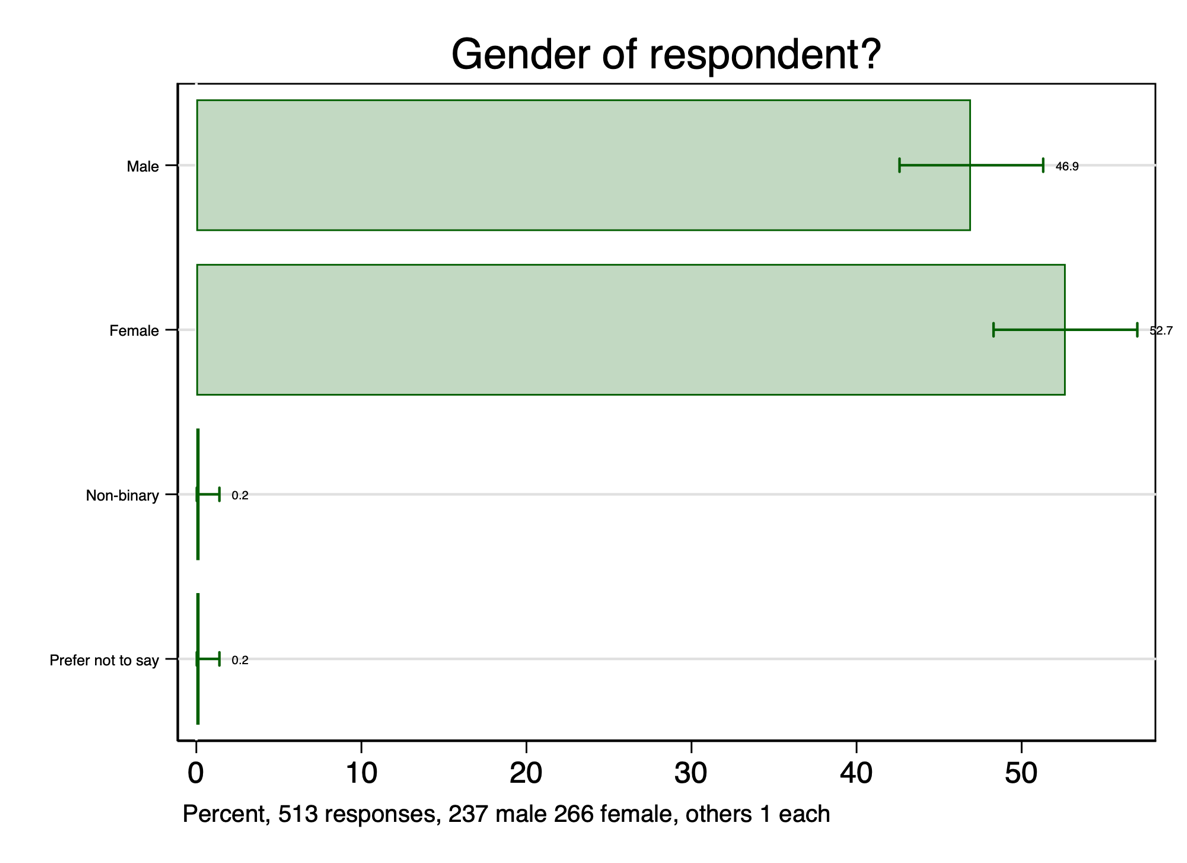 | 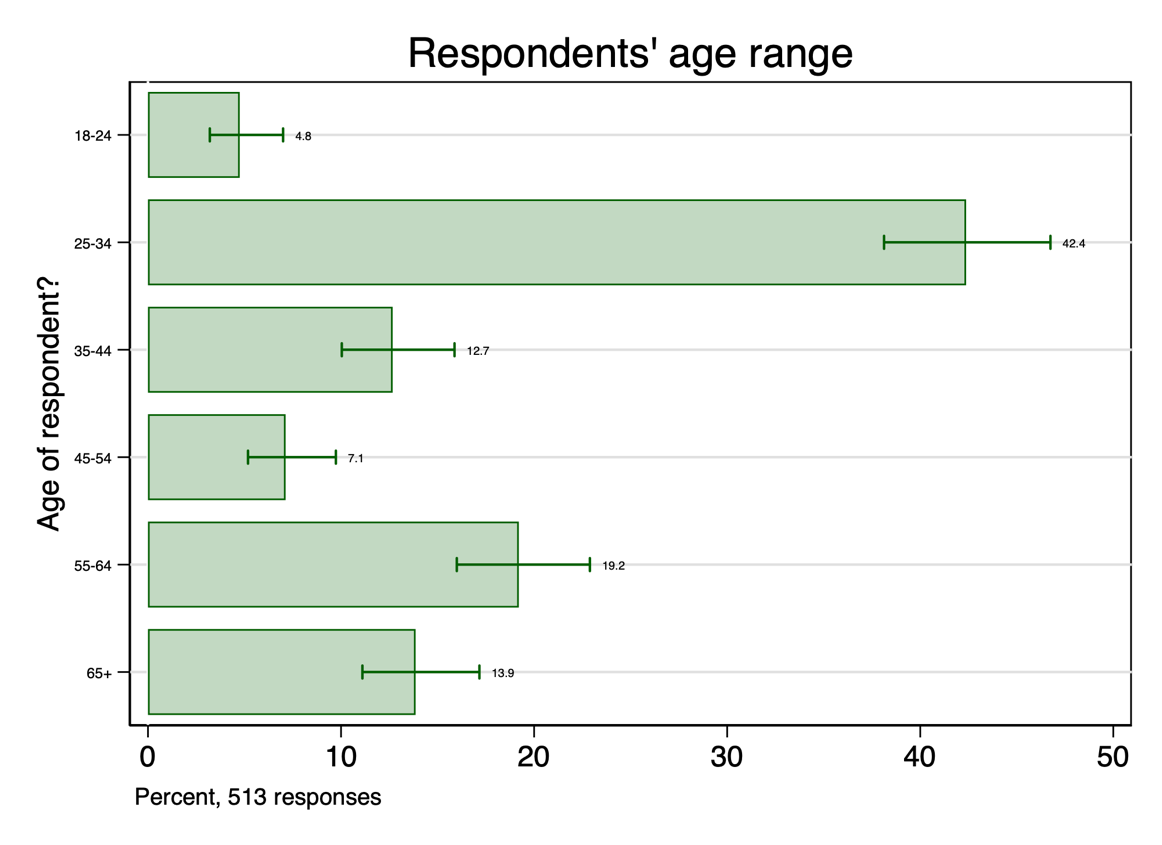 |
| --- | --- |

| 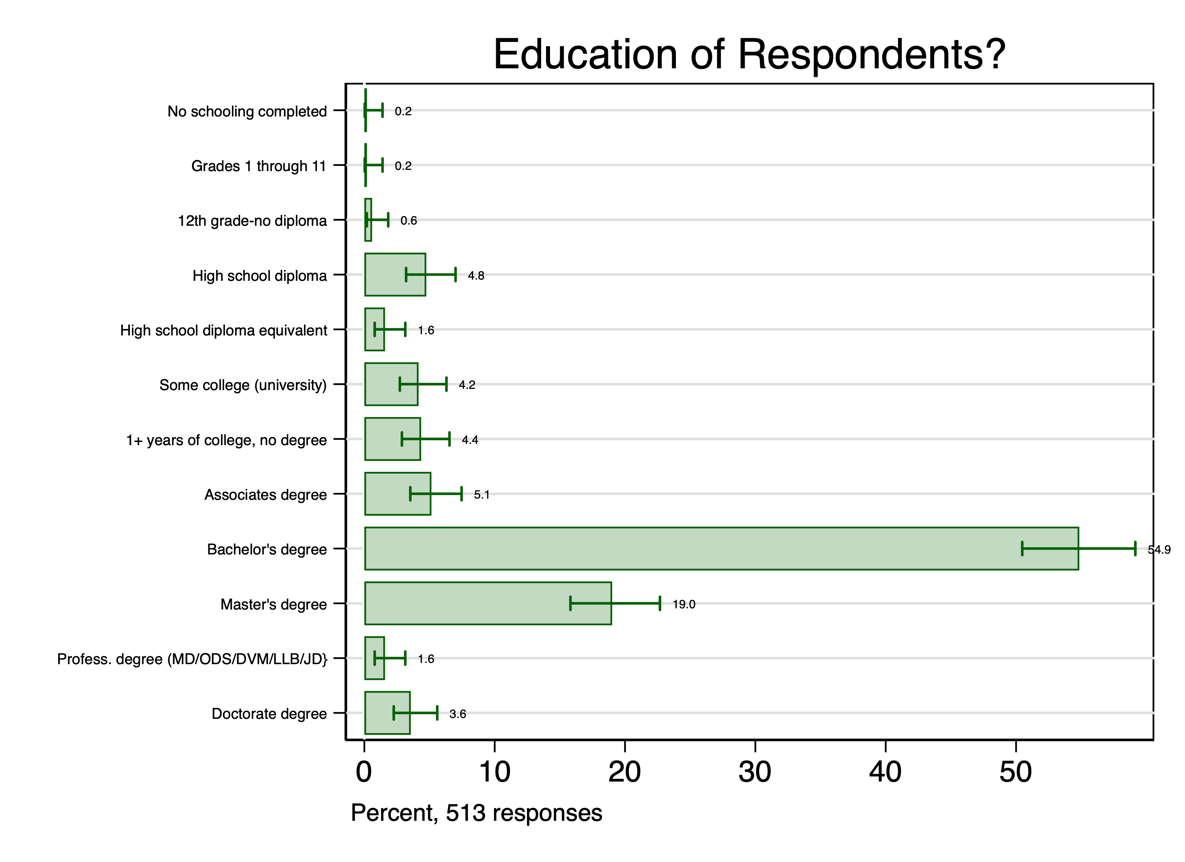 | 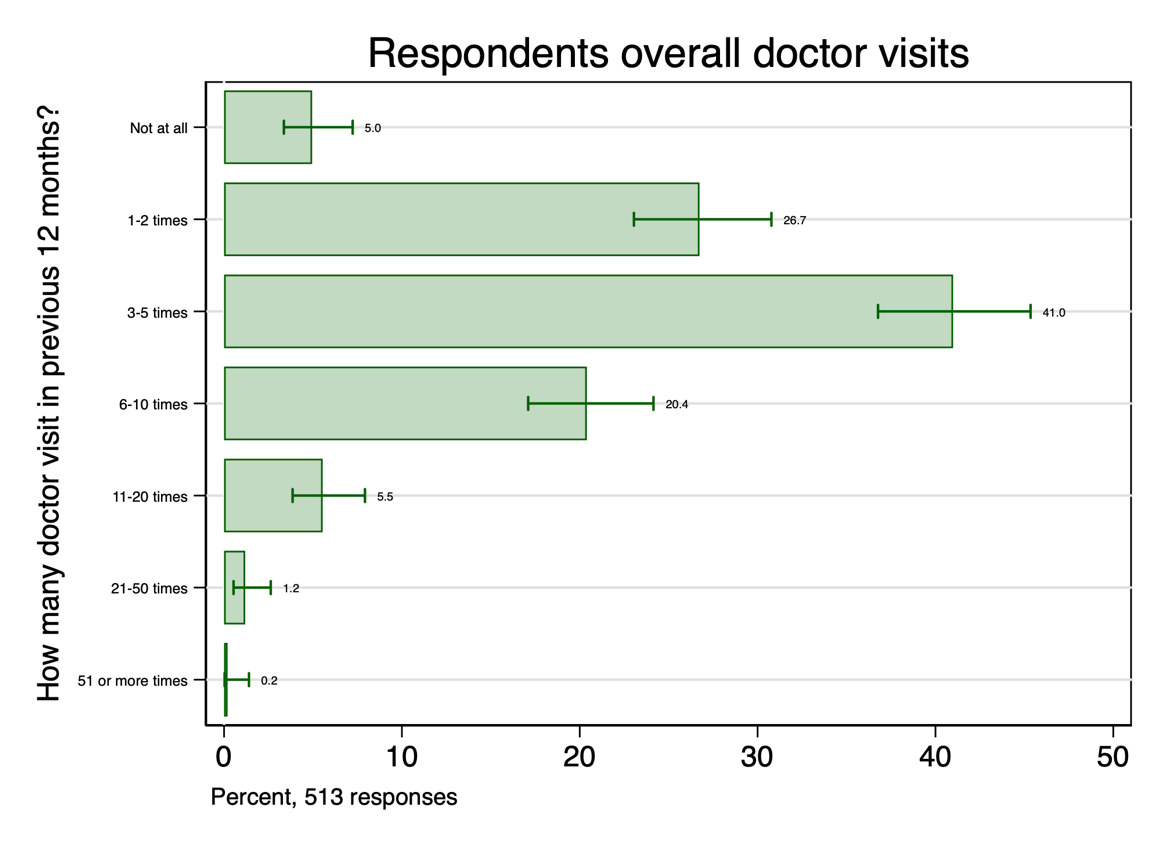 |
| --- | --- |

| 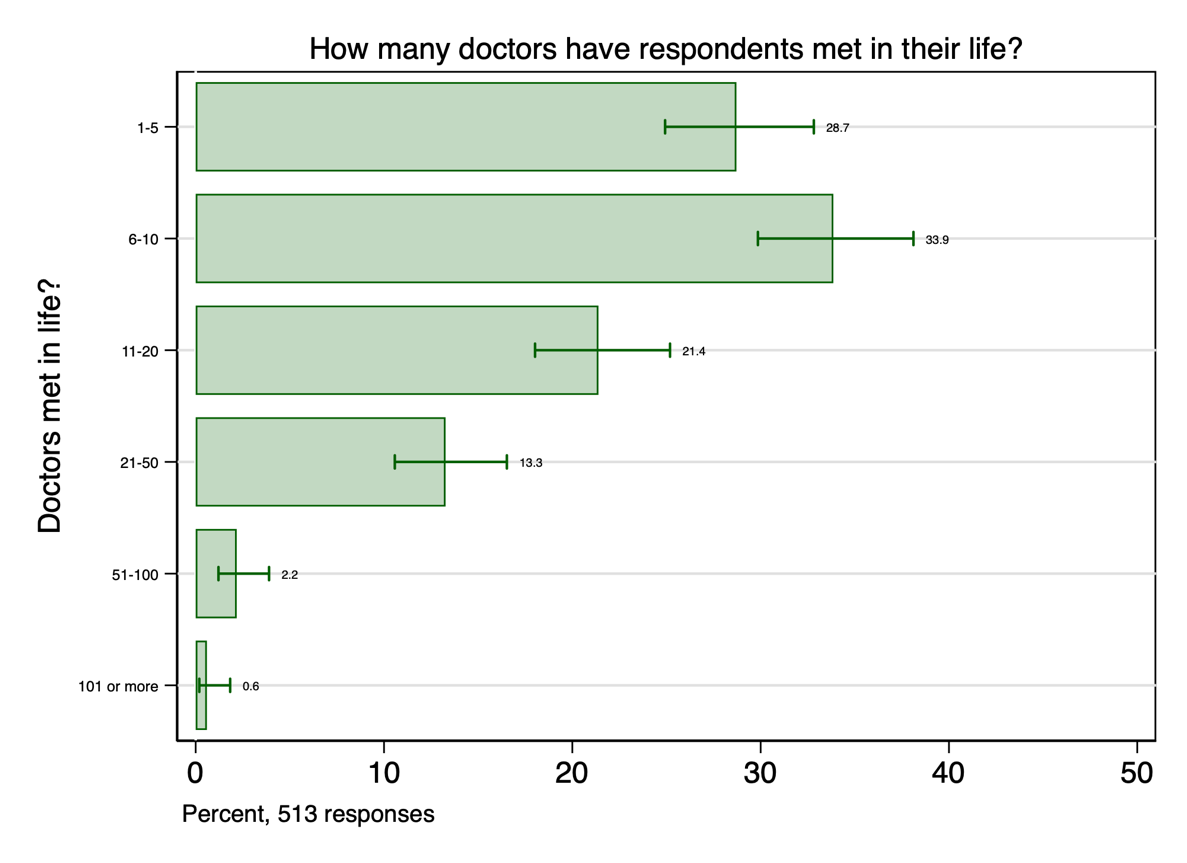 | 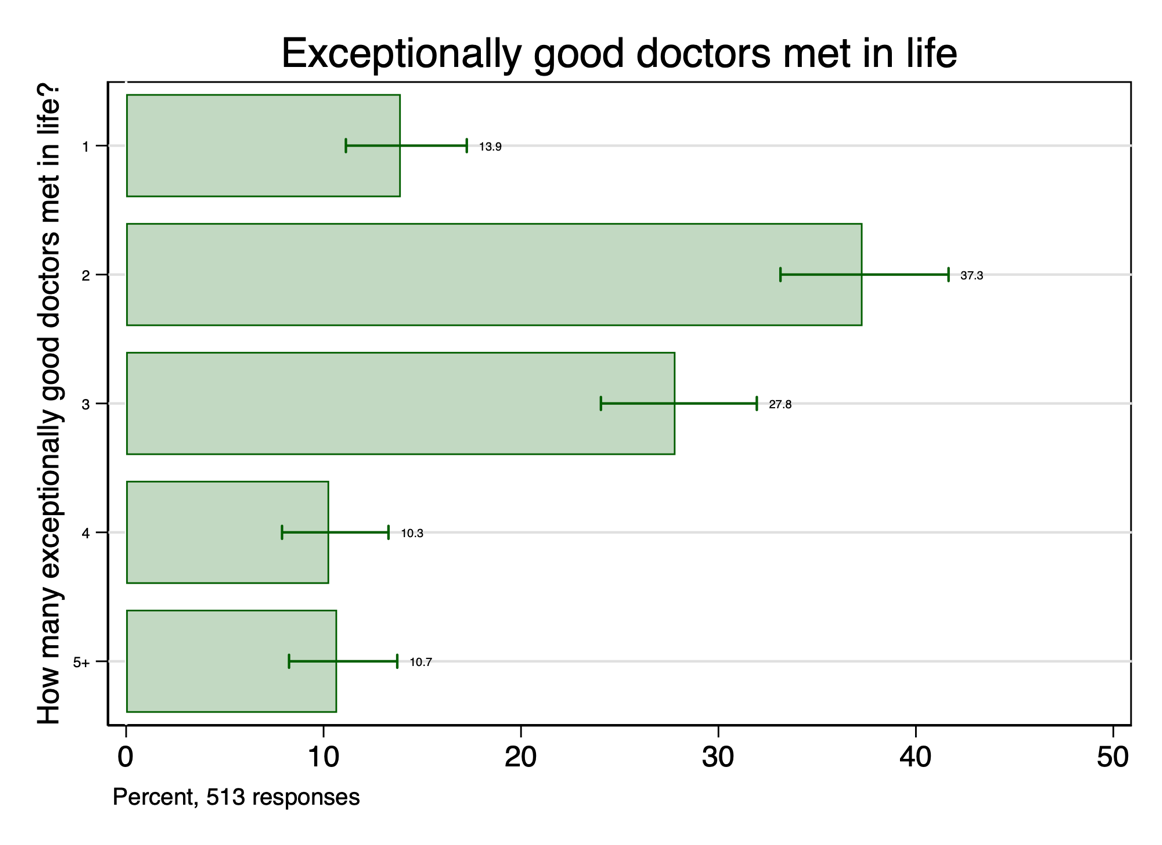 |
| --- | --- |

| 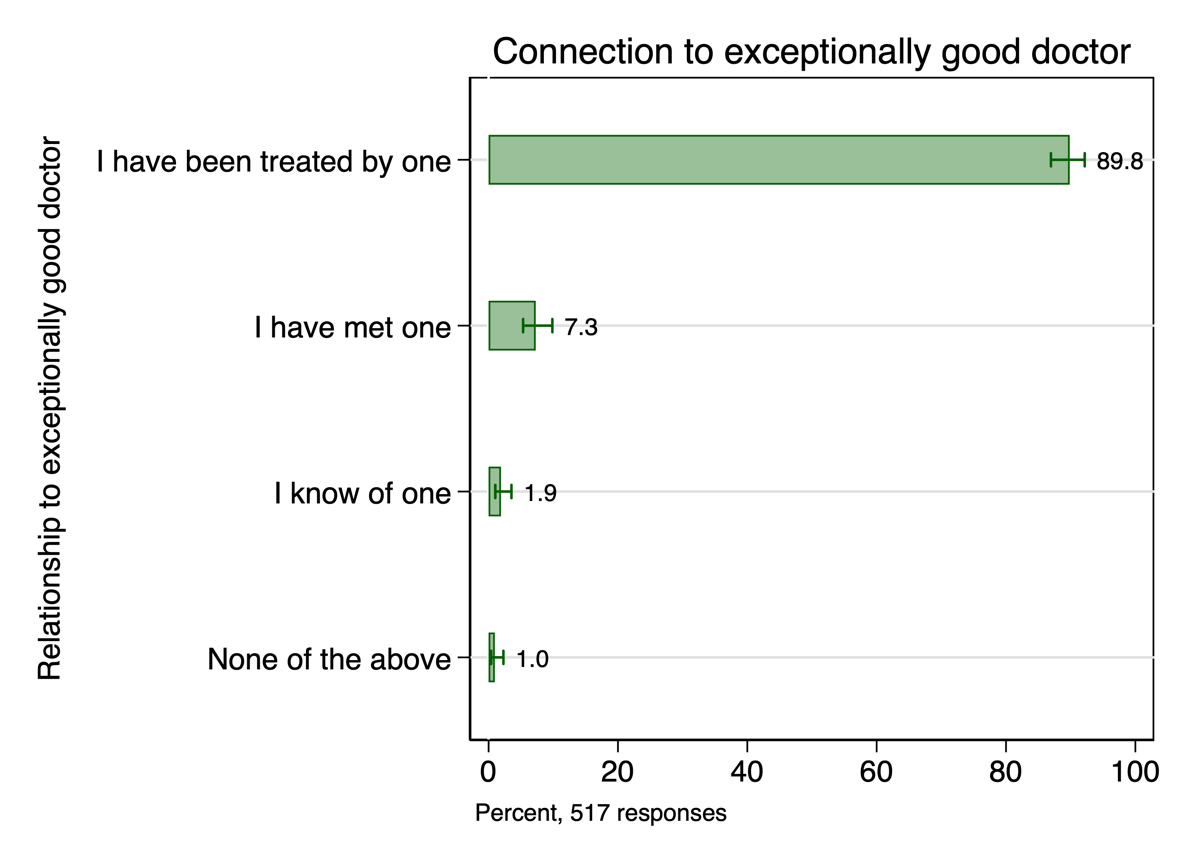 | 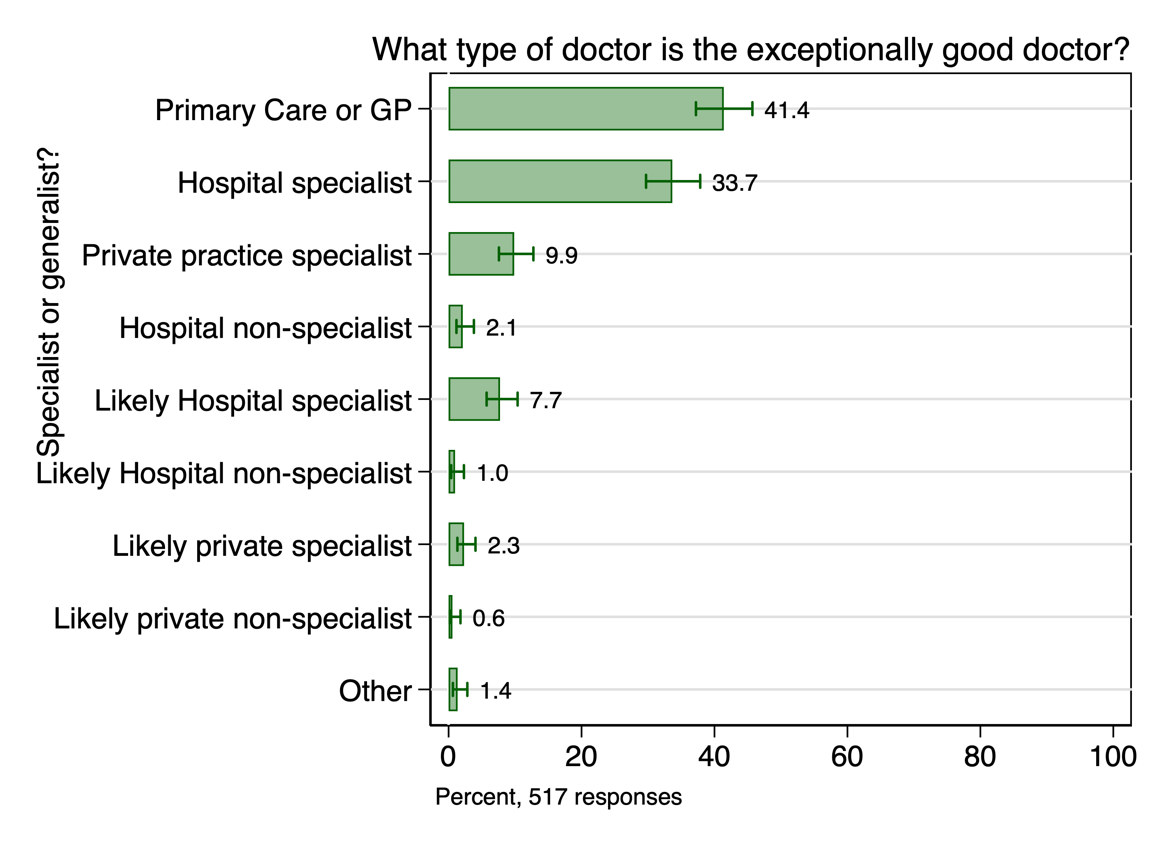 |
| --- | --- |

| 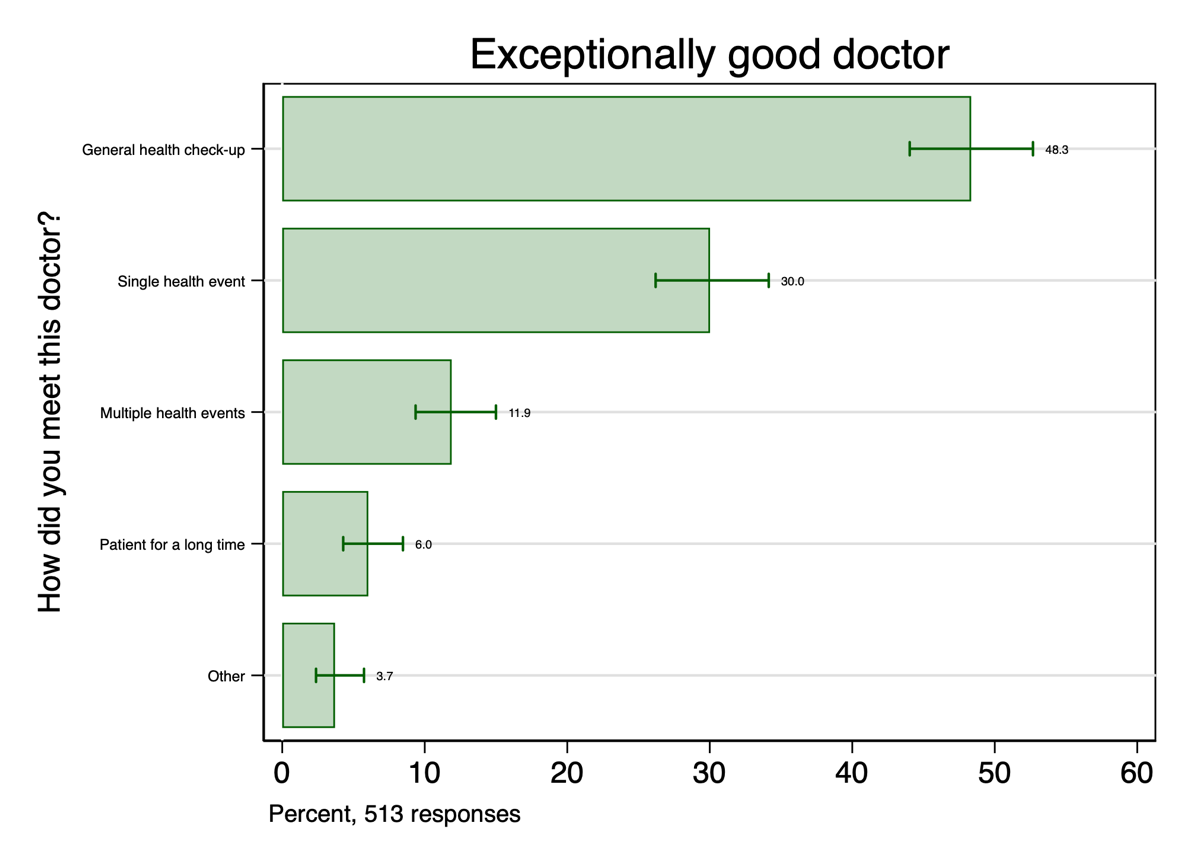 | 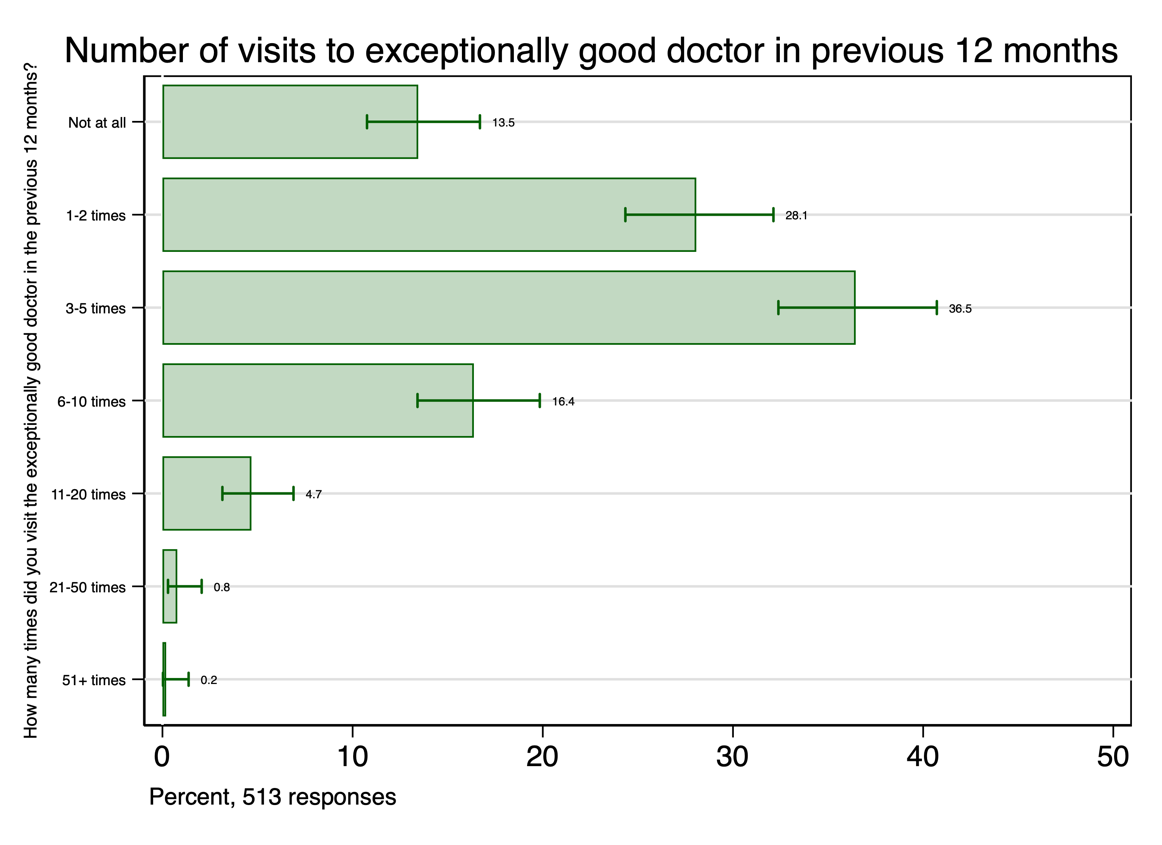 |
| --- | --- |

| 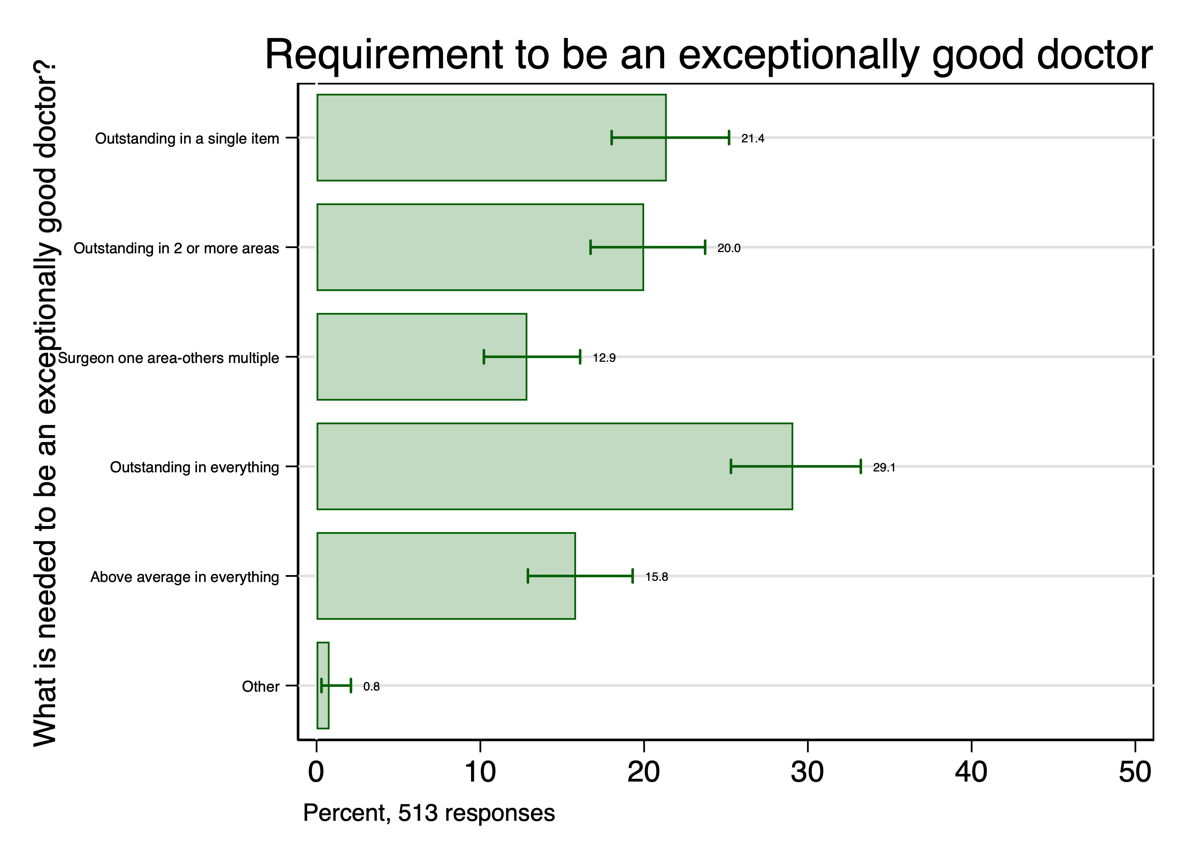 | 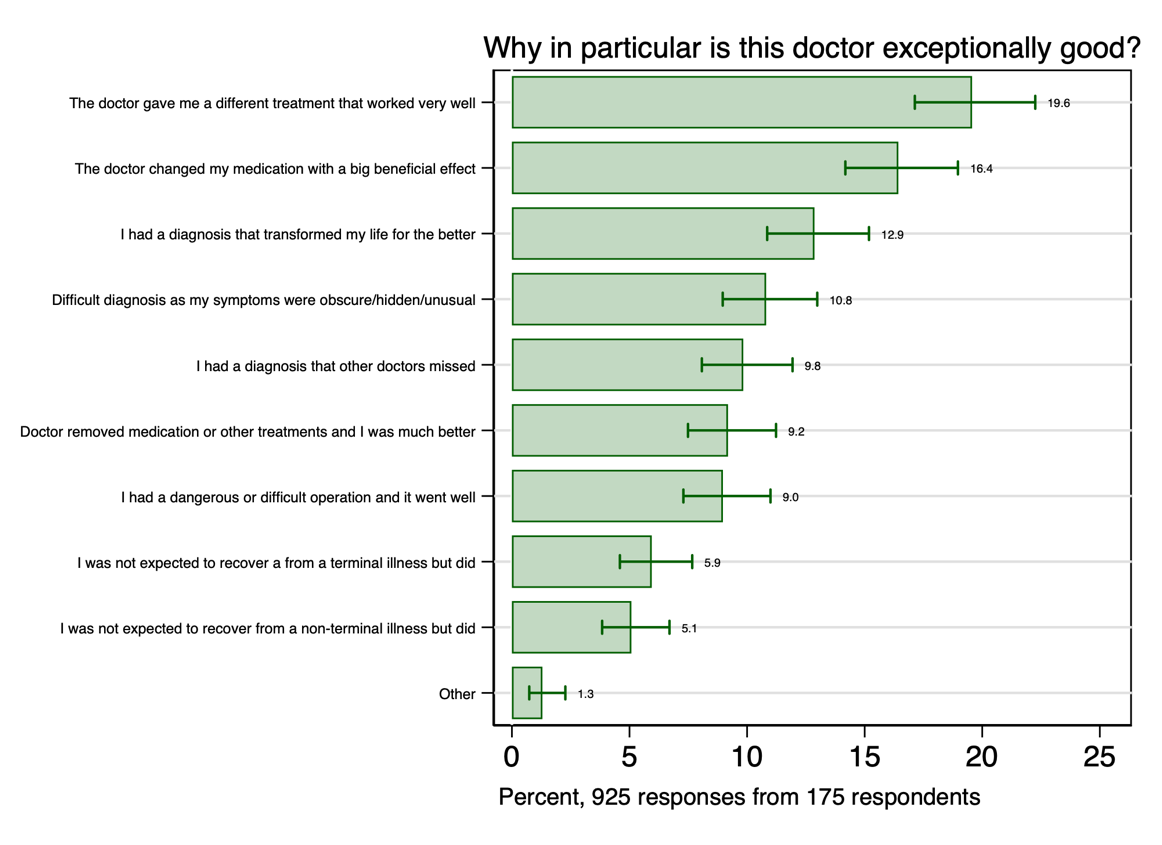 |
| --- | --- |


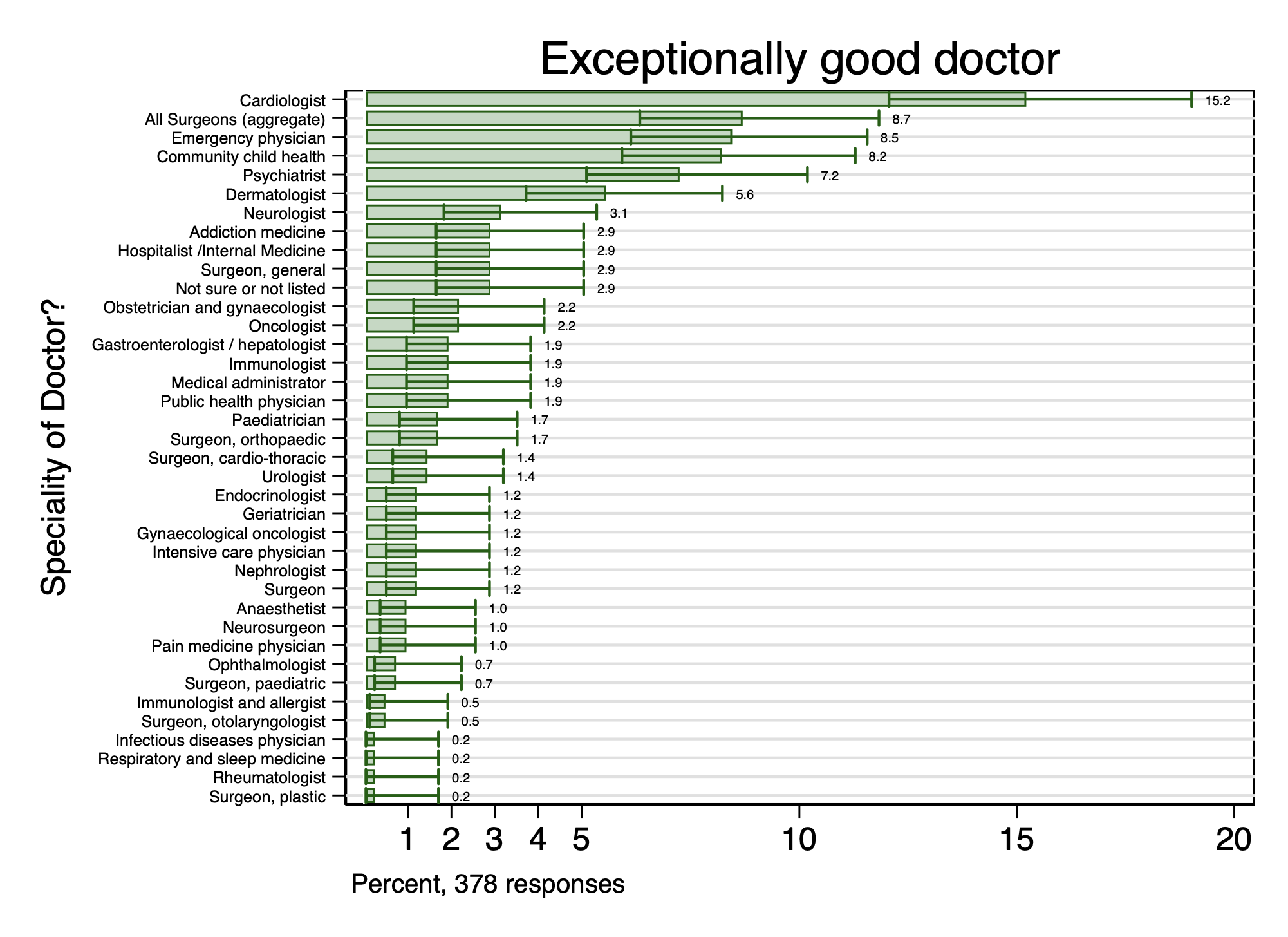


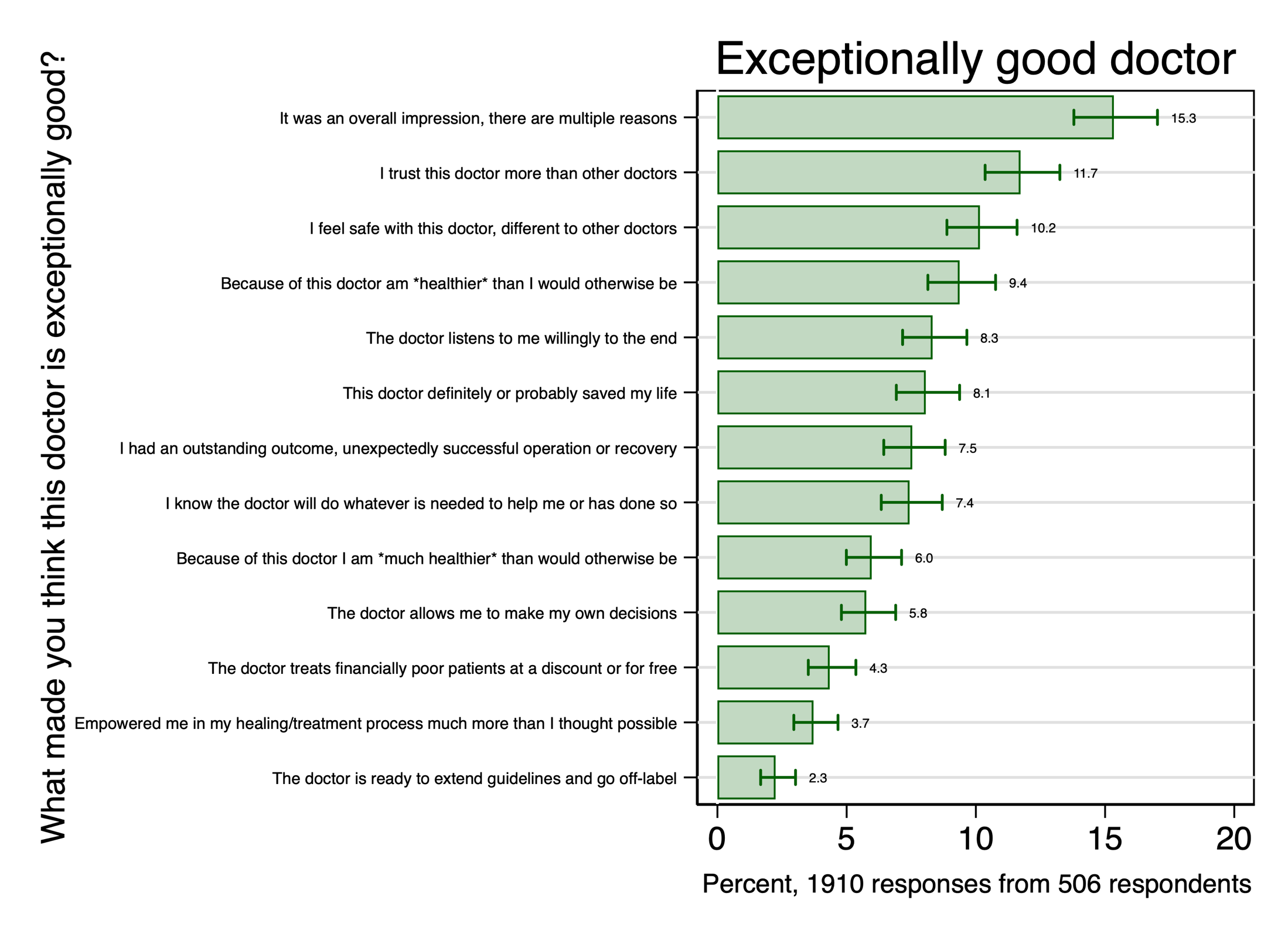


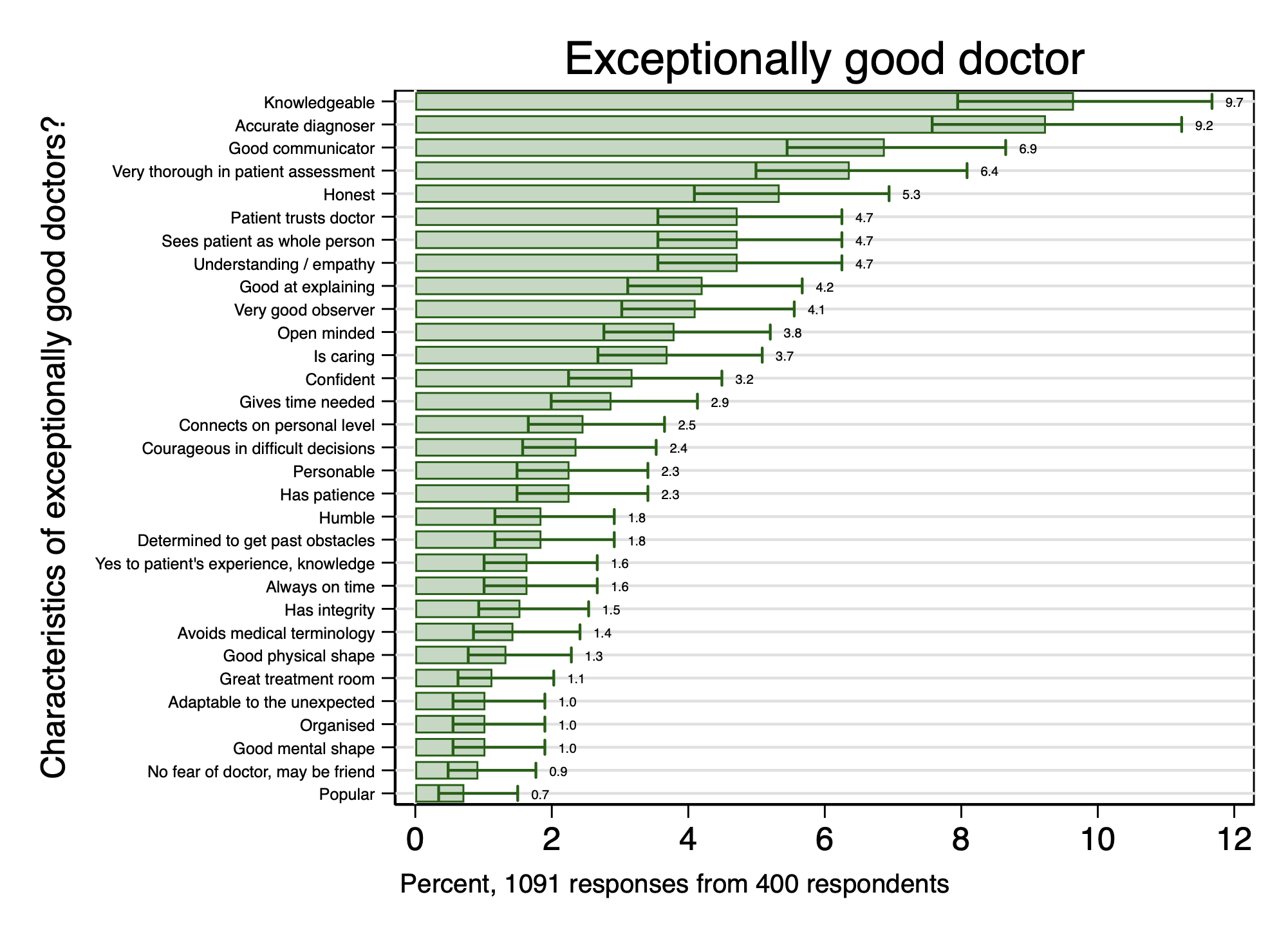


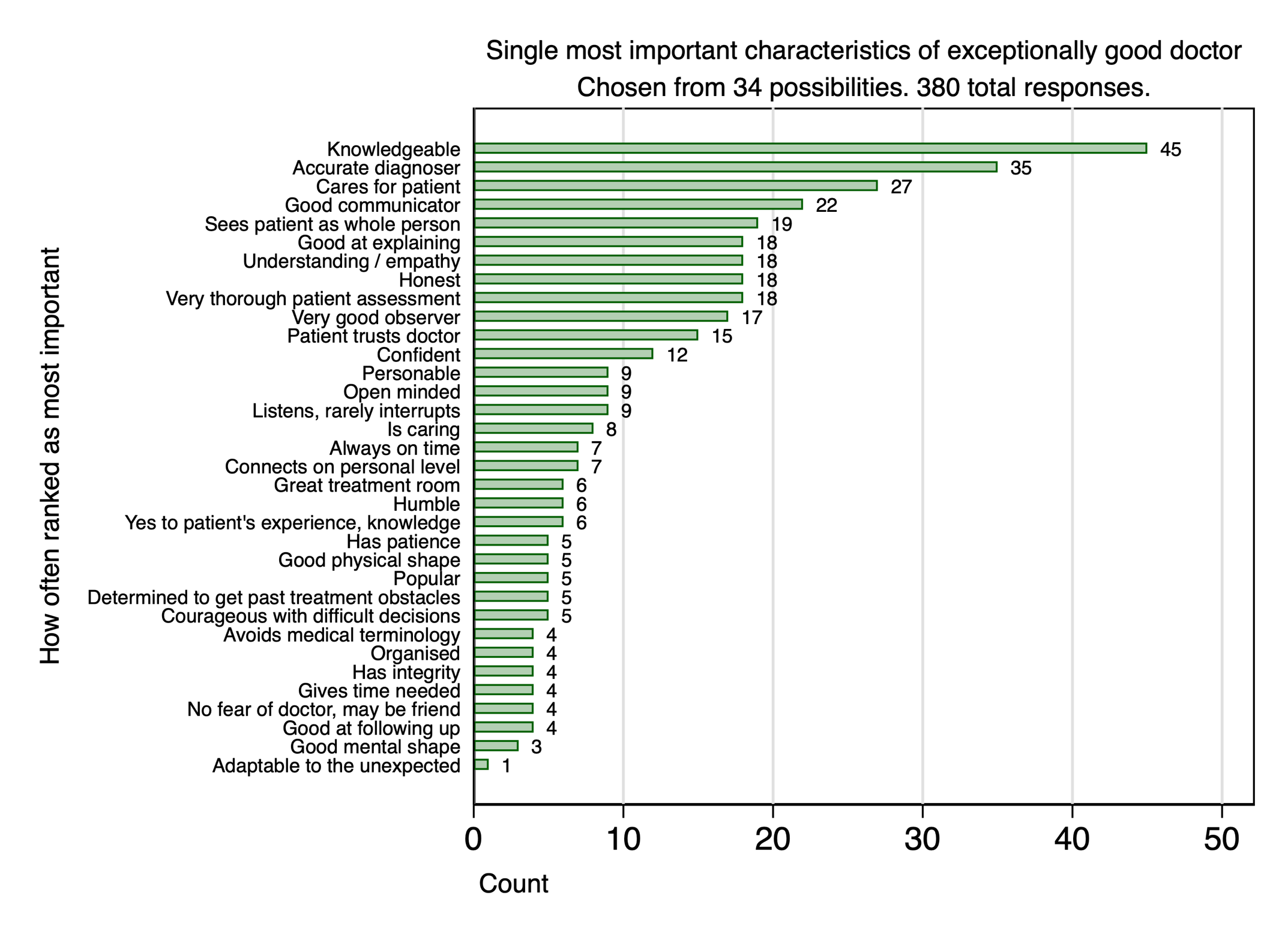


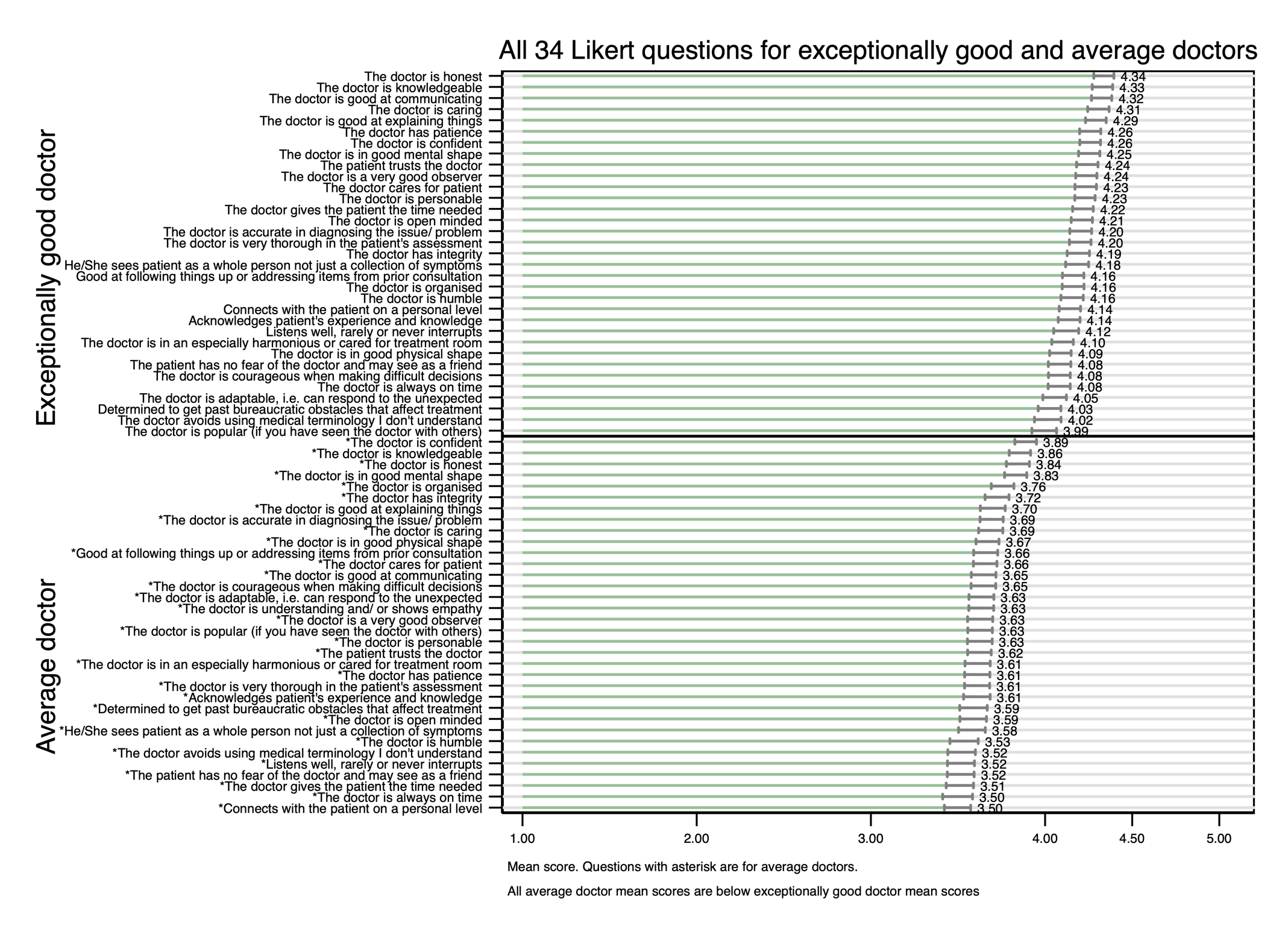


# Appendix 2 Respondents’ experiences in their own words ordered by category and length

Question 16: Would you like to tell us about your experience with this exceptionally good doctor in your own words? (Optional) That would be very helpful because every individual experience can be very different and currently little is known about exceptionally good doctors.

## Category 1: Respondents’ own statement of what is needed to be an exceptionally good doctor

| **Respondents’ own statement of what is needed to be an exceptionally good doctor** |
| --- |
| She was very patient in listening and also communicating with me about my father. She gave me a clear understanding of what the situation was and what to look for in the future. My dad was being treated in a hospital and she was overseeing things as she was a friend of my friend. Doctors usually have massive egos that is sometimes not earned by them. They don’t like being asked questions. They have their juniors to block these types of communication. They also follow protocols blindly most of the time without using their common sense or really observing the patients.  This neurologist was really exceptional because she liked discussing things and was open to questions. She also had the acumen to change course in treatment as the patient showed different symptoms or improved without just sticking to "protocols". I had a really bad experience with incompetent doctors so this was really refreshing to see. It was not about money at all as I didn’t pay her much. She had a sense of humanity and a genuine sense of caring nature. It made me feel secure and I could explore information myself and consult her. Some of the doctors, especially during and post corona have lost their focus and their ethics. They do not question or even care to question anything. This is very unscientific and makes me nervous for other people as well. A doctor without common sense can be dangerous. They ought to have discipline and dedication regardless of the circumstances. They can’t give up easily and shouldn’t, just because there is some pandemic or the statistics suggests that humans die after all. Doctors should be inventive, treat people as humans who are owed dignity and have a right to live. They should be updated with technology.  I saw hospitals that had really low level sensors and gadgets. This is embarrassing. Doctors who mundanely do duty just to make money can never be good doctors. They should try and strive to make the medical industry better. They should have enough courage to question things. It is not just about cramming antiquated books. Right now, I am thoroughly disappointed with the general set of doctors we have. Some of them are downright immoral and bereft of any guilt. A great doctor would do justice to the oath they took. |
| Patients don’t care about their physician’s medical school grades or other accolades—they want to feel that they are in good hands. A good doctor knows how to make a patient feel as though they are being cared for, that their concerns are valid, and that they are being heard. |
| this doctor was really good nature. the good physician treats the disease, the great physician treats the patient who has the disease. this doctor treat the patient, not the disease. |
| I just think what makes him exceptional is his ability to really listen to the issues I have and how he helps with those issues. |
| Good doctors will provide good tips and manage all the health care. |
| good doctors are good communicators |

## Category 2: Treatment examples given by respondents

| **Treatment examples given by respondents** |
| --- |
| The man remembers me from past visits and remembers my past history (although he could be checking a laptop to refresh his memory). He asks questions, and if I have any complaints, he explores them. He takes his time with me and isn’t in a rush to get to the next room/patient. He tailors my treatment (i.e. prescriptions, recommendations) to my personal situation at that time, not a generic "do this."  He schedules whatever tests (i.e. lab work) he thinks are necessary. He monitors my progress on various prescriptions. He knows what he’s doing. If a follow-up is necessary, he schedules it. He is friendly and has a good sense of humor, but he doesn’t clown around. He cares about my well-being; I’m not just another name on his laptop. He follows up on my needs. If one of my problems/ailments is outside of his specialty (i.e. when I had an ear problem which turned out to be trivial), he sends me to specialist. |
| He was my pediatrician/primary care doctor from around 6 years old until I went off until college. He was intelligent, knowledgeable, had a great sense of humor, and as I got older, respected my privacy and knew what he should and shouldn’t share with my parents. There seemed to be mutual trust and respect.  I have not had that kind of relationship with any doctor I’ve had as an adult. I think my esteem for him has only grown as I’ve gotten older and experienced the dreck that passes as fully licensed physicians For example, my current primary care doctor is mediocre at best and I feel makes a lot of assumptions that lowers the standard of care provided. I am hesitant to even see him at this point. We are not on the same wavelength and I have started to believe googling symptoms wouldn’t get any worse results than seeing him. |
| He is the kind of doctor that likes to solve puzzles. So if a patient comes to him with an illness he won’t just rule things out and make a quick diagnosis, he will try his best to figure out the cause of the problem. He spends time with his patients and doesn’t have his hand on the doorknob ready to leave the exam room and get to the next patient.  ’d say that he is a doctor for the right reasons, he really cares about taking care of his patients. When he came up with the right combination of medication to help me and I felt better, I was so happy that I bought him a bottle of wine and he was very appreciative. I had been misdiagnosed by other doctors so I really appreciated that he kept on trying different things until something worked. |
| I first met this doctor when I interviewed for a job at her clinic, I later became her patient, and worked for her for 26 years. We are both retired now. I think she was an exceptional because she was truly caring to every patient. She spent time with each patient and listened to each of them. Sometimes she spent so much time with patients that others got impatient, but when it was their turn they received the same care and time as the one before them, and they ended up coming back to see her for many years. When difficult cases came up, she did not give up. She would call specialists in the area for advice or send the patient to them. |
| I met her 30 years ago. My regular physician was too busy to get in to see so I took an appointment with the "new kid" at my group of doctors. In the first appointment she started listening and helping me with multiple issues I could never get help with. I still remember her listening to my symptoms and educating me on what I needed to do to feel better. Getting me to take the medicine that would eventually make it easier for me to breathe, sleep and thrive. No one had ever helped me so much. She is an expert communicator, detective and so very caring. She continues to take good care of me after all these years I trust her completely. |
| Although I have had the good fortune to have received treatment from several good doctors my family practitioner is exceptional. From the very first meeting, his easy-going personality and obvious knowledge of current healthcare practices put me at ease. I was especially impressed with his thoroughness and the way he made me feel like my concerns were important. As an older male patient, I especially liked the way he targeted in on those things they may affect men of a certain age and made sure any related testing was either immediately conducted or referred out. Each time I’ve visited him I expect and receive a pleasant experience that has satisfied my healthcare concern. |
| Whenever I see him, it is just for a health checkup, but he is also extremely nice and personal. He likes to talk about my personal life and what is going on with it. He asks important questions about how I’ve been feeling, and he always has a friendly demeanor about him. He actually cares about my well-being and it shows. I am almost the most comfortable I’ve been with a doctor when I see him. Some just want to get you in and out, but I never got that feeling with him. He seems like he is just as much a friend as he is a doctor. |
| Every time I visit with my doctor, I hardly have to wait for too long. My doctor is very personable. He takes the time to ask about the symptoms I may be experiencing. Then he gives me a thorough checkup. He always explains my symptoms in language I can understand. He’s never in a rush. It’s like I am the only patient. He’s caring and very passionate. He discusses the treatment options and I always trust his opinions about what I should do. He always answers all my questions or concerns. I feel I am in good hands with my doctor and wouldn’t think about changing. |
| I have had my doctor for 23 years and I am still seeing her to this day. She belongs with my health network. Found her 23 years ago. She has been the best doctor that I have had my entire life. Dr. before her was a quack. She has been able to identify all of my ongoing health issues. And she keeps in contact with me especially if she has heard from me in a few months. She makes me feel so calm and peaceful each time I leave from visiting her. She listens, and is very sympathetic and empathetic to my health needs. |
| I don’t have much to say, the Doctor is excellent. I am the mother of 2 boys and 1 girl and they all went through the hands of this doctor. I remember that once one of my children had a more serious problem and the doctor gave him weekly follow-up care, and this was not mandatory on his part, he was always very nice, polite, and really knew what to do in any situation, no matter how difficult it seemed to be, and the most important thing is the tranquility and security that he passed to everyone. He was very reliable and friendly. |
| I made an appointment with this doctor shortly after moving to Connecticut. He was more outstanding than I can express in words. I have 5 children and he treated all of us. He was incredibly talented at making an accurate diagnosis and encouraged me to contact him at any time - day or night if something was wrong. He attended to me while I was in the hospital and carefully reviewed the work of any specialists. He was caring, compassionate and truly dedicated himself to the health of his patients. I was lucky to have him for 10 years before he retired. |
| He was the father of one of my best friends growing up and reached out to my parents to tell them he could see me as my primary if they wanted. He was always very nice and professional. He passed away when I was about 17, but I always remember him as kind of the template of what I think a doctor should be: smart, caring, personable, and he never treated me like a child, even though I was one. He just gave off this calm, confident air of authority that made me feel safe and well taken care of. |
| Dr. Cutchins was one of those doctors that made you feel very comfortable when you were in his office, he knew what he was talking about and he was always right. He took the time to listen to you and let you ask as many questions as you needed to. He wasn’t one of those that looked at his watch and only let you talk about what problem you were there for. He even made my husband look forward to going to the doctor and if you knew him you would know that is surprising. |
| To me it is hard to explain but I will try. Seeing this doctor was not stressful at all. I never felt as if I was being talked down to or against or anything like that. I felt as if the doctor and I were on the same team. They explained everything they where doing and allowed for questions. They carefully answered any concerns and were happy to discuss anything. I was very nice. The most comfortable experience with a professional that I do not know personally. I wish all doctors were like that. |
| I had a harder time responding to a few of the prior questions because of the nature in which I met this doctor. I worked for the same hospital that this doctor worked at. However, my role was to assistance patients from developing countries through their appointments. I met this doctor as she was the pediatric surgeon that I often interacted with. I saw the impact she made through not only her exceptionable work, but through her kindness, understanding, and respect for every patient. |
| This doctor has been treating me for various health problems for almost 4 years. He remembers even the details about my home life I have told him before. When I expressed anxiety over a procedure, he ordered a prescription to calm me down the day of the procedure. He returns calls when I call with a question or a refill. He offered an alternative for another procedure that I was dead-set against. He seems genuinely concerned with my concerns, and addresses them. |
| I first met Dr. S. while working as a floor nurse in an oncology unit in my city. I noticed that he was the only doctor who routinely called (at 7:15 am) wanting to know how his patients were doing. He would ask for specific lab work and was always polite and caring. It was over many years that I learned his patients seemed to get well and discharged faster than other oncologist’s patients. His patients also had a higher cure rate. |
| Dr. B was a very intelligent and caring doctor with a great deal of experience. I was surprised to find him in such a small town. I think he liked it that way. He was patient with me, took his time, and was thorough. He was able to come up with quick and correct diagnoses. In addition to all of that, he had a great bedside manner and joked around a bit. It was a pleasant experience in an unpleasant circumstance. |
| Both me and my wife have the same doctor, Dr. Jackson. He is very approachable and tells me a lot of things. If we come to him and need a specialist, he is very willing to do whatever it takes to get us to see someone. He has told me about the non-medical things, like he really likes motorcycles. He also told me about some of the research that he does. Very easy to talk to. |
| I have been going to this doctor for over 40 years. I met her when she was just starting her practice in ob/gyn. She delivered all 3 of my children and I still go to her for annual checkups. Her manner is exceptional and she is truly caring. When she found me crying because of something a nurse did, she took over and did the procedure herself, although it was a traditional nurse job to complete. |
| my doctor is exceptional because he is good cardiologist. about 1 year ago my heart was blocked and my doctor treated me very well. and he has enough knowledge for this diseases. now taking this treatment and advice i am much better than previous days. i also go to the doctor for regular check up and he told everything after the test. and he also has a good prediction about the diseases. what will happened. |
| Some years later my mother was dx with colon cancer and I mentioned she should go to Dr. S. He cured my mother and she has been cancer free for over 15 years. I have never met a more exceptional doctor. I saw daily how he cared about all of his patients and seemed to be born for what he does. He is also very involved in our community. He is one of a kind. |
| This exceptionally good doctor is a specialist in his field of neurology and otolaryngology. he is a surgeon and also teaches in the hospital where he is employed. he is extremely kind and has an exceptional bedside manner. he is extremely conscientious about his work and highly particular in his work. he has treated me for my condition for 30 years. i especially enjoy conversations with him and appreciate his kindness. |
| I saw a psychologist (psychiatrist was the closest option) for about 4 years following a severe bout of depression caused by overlapping losses and uncertainty. He really helped me to find myself during trying times, and always remembered details about my experiences despite having many clients and being a professor. He was always on time with his appointments, and was always curious about my life when I met with him. |
| My experience with this doctor is that she takes the time to go over my file with me in detail. She asks me questions about my lifestyle and is willing to work with me to help me achieve my goals. One of my goals was to get off of my blood pressure medication. She listened and agreed with me and I succeeded in being able to get off of them. |
| He is a very capable doctor, and he listens to what his patients have to say. He is nice and kind ,and he keeps up with the latest information in his field. He pays attention when I ask him a question and if he is not sure of the answer, he will research it and get back to me. He never makes me feel like I am unimportant to him. |
| The exceptional doctor I am referring to helped/is helping me through several medical issues. She is a great listener, and I feel as if I am the only patient she is treating - she never "hurries" through an appointment. This in of itself makes me feel as though she really cares about me. She explains/communicates very well, and is not bothered if I ask her the same questions several times. |
| I went to him because he was recommended to me by a friend of mine. The thing that made him stand out for me is that he listened to me and didn’t make me feel like I was wasting his time. He made me feel like he was there for me. I felt like he really cared about and listened to what I had to say. |
| My doctor listens to any concerns I have and carefully explains any recommended suggested medications, tests, or procedures. She answers my questions fully, and returns my messages via the electronic care system promptly. I have given her feedback about medications, and she was receptive to my ideas as far as increasing or decreasing dosages based on how I felt. |
| The doctor is efficient, quick, and knowledgeable. He is able to dance around and get approval for medications which my insurance uses many forms of bureaucracies that bog down other doctors in other fields. He knows immediately what treatments I need, what conditions to treat, how to treat, and is very quick in deciding and expert in administering treatments. |
| I have nothing but good words for him. He is a very exceptional medical practitioner. He’s patients come first. He makes good sure that I’m aware of whatever he is doing by passing deep and intellectual knowledge. He handles everything with care and purpose. He is such a kind heart. He is also very smart at everything he does. |
| My current doctor has always gone out of his way to help me (and listen to my concerns). Oftentimes, he has been ahead of the specialists I have seen. More than anything though, he is available. Last year, I needed to see this doctor immediately. He made time for me the very next day despite his full schedule. |
| I found him by chance and on the first visit immediately adapted to him as a person and a doctor as he was friendly, appeared to be honest, and was very upfront in his conversation with me. My wife and daughter soon went to him and both felt he was the best doctor they have ever visited with. |
| THE BEST DOCTOR WHO IS OF WELL AGED AND EXPERIENCED IN GYNAECOLOGY. AN EXPERT IN BRINGING ANY COMPLICATED SITUATION TO A NORMAL ONE. A LUCKY CHARM WHO IS FILLED WITH COMPASSION TO TREAT THE NEEDY AND THE POOR TO FROM HER OWN EXPENSE. A GUARDIAN ANGEL TO MANY AND A BIGGEST TO HER ORGANISATION. STAY BLESSED MA |
| He is always personable and greets you when he comes into the room. He remembers what care has been given in the past and is willing to discuss ongoing care options. He can explain everything in a manner that I can understand so I can communicate his care to any specialists that I may need to update. |
| I found my doctor a few years ago. He’s with a small practice so he can spend more time with me when I make an appointment. He’s great, nice and friendly and listens to my concerns with compassion. He has helped me with minor and major ailments and he’s very quick about referring me to specialists. |
| they are very caring that you can tell by the things they say and do. They started a charity for abused children and when the police or social services have a child they know of in that situation they call her and she helps the child and does other things like find therapists for the child |
| All I have to say about that doctor is that he is my alumnus. I could not recognize him but he recognized me. I am more proud that he is my student than that I am a teacher to him. A physician has the patience to care for patients and have all the professional devotion |
| He has really great communication skills and adapts his level of medical information based on what he knows I will understand. He never assumes something is too complicated for me to grasp. He has also helped me thru some significant health problems and brought me though them with complete frankness about my situation. |
| I am now 73 years old. I am acquainted with him about 20 years. I like him very much for his very friendly approach. He is a very good listener and very much empathetic to patient. He is very much sensible and knowledgeable and a good stress manager. He also follows strong ethics. |
| The doctor is great person who cares about more than just my health. He is genuinely interested in me as a person. He always is eager to talk to me about anything. This doctor wants to know about my life and habits and basically as much as he can about me. |
| He has been my doctor for more than ten years. I chose him as an option when I select a PC from my healthcare plan. Although I had the option to change my PC I have never felt the need to change. He has guided me through a few serious issues. |
| He is Really an exceptionally Good Doctor, He Treated me Like as Colleague Not Only Me, He Treated and Gained Their Medical History Like the Next Door Boy and His Prescription Selection was Too Good for all People, It Cures all of Petient’s Disease within Couple of Days. |
| He is Really an exceptionally Good Doctor, He Treated me Like as Colleague Not Only Me, He Treated and Gained Their Medical History Like the Next Door Boy and His Prescription Selection was Too Good for all People, It Cures all of Petients Disease within Couple of Days. |
| I have been with my doctor for 25 years and he has saw me thru many difficult medical situations in my life. Taking the time to actually talk and explain anything you ask him is a very important part of his doctoring. Friendly and a great bedside manner. |
| She is a very caring person. She is easy to talk to and always answers question in a very under stable way. It is obvious that she cares about you as a person and not as somebody that is only a source of income for her. |
| I met this general practioner doctor in a community where I’d moved to about 9 years ago. I really liked him. He had a great sense of humor, spent time with me, and was very knowledgeable. He didn’t immediately shunt me off to a specialist. |
| Every visit my doctor is attentive. She listens to what concerns I have and answers to the best of her abilities. If she is not sure of something, she gives me websites and places on where I can find the best information on the subject. |
| i would like to say. the doctor would often come to my house to give me and my husband medical treatment. man of good character and good patience. patience can only be learned from him. he forgets that we are patients and treats us. |
| All of the people (about 3 I think) I have sent to him speak just as highly of him as I do. I cant imagine having more confidence in any doctor. I look forward to having him as my doctor for many more years |
| My primary doctor is patient, exceptionally up to date on the latest trends in medicine, conservative but proactive, never rushed, honest and forthright without being harsh, a very good listener, very good at follow up and knows how to navigate the medical world |
| She is a really good listener, and is willing to listen and understands things that I tell her or ask her about. She personally answers her messages very quickly and gets back to me promptly with great answers and suggestions. |
| As a child, my pediatric doctor made me feel like I was going to be ok, no matter how many stitches I needed at the time. He still has my old drawings hanging in his office, 20 years later. |
| Instead of just telling me what medications I should take, this doctor listens to my concerns and we then decide together. He always takes his time with me, so I don’t feel rushed into making any decisions. |
| He didn’t seem to be bothered by time. He patiently answered all my questions and we discussed medical treatments. He was never judgmental and never told me my problems all come from needing to lose weight. |
| My personal doctor was treating me very well. He asked all types of my issues in by body oriented and gave the treatment. Then I feel very relaxed. He helps me a lot. I like him. |
| She always makes me feel better before I leave her office. She shows concern for me as an individual. She doesn’t make me come in for every little thing as she knows my health history. |
| he’s deceased now. He was great! Was always ready to listen and actually took the time to answer my questions. He’d been at it for 30+ years. My doctor now sucks compared to him. |
| When I approach the doctor he will give me a good treatment. Only when I go to him will I be at peace. Only when I go to him will I be at peace |
| I am HIV pos., this doctor is a local doctor and I was just paired with them when signed up for appointment. The doctor is skilled and community minded. I’m lucky to have her. |
| He was an oncologist of sorts at John Hopkins. He was very knowledgeable, friendly, empathetic, and communicated each step well. He explained the procedures, and helped her overcome her fear of MRIs. |
| He is friendliness.He was willingness to invest time with me.HE was so caring and treated me good. He has perfectly common scense to comminucated with someone. He was a knowledgeable person. |
| this doctor is very friendly and take time to listen to my medical problems The doctor tale as a person and does not treat me as someone who is an idiot |
| She is very understanding. She listens to me and what I have to say. She recommended things but let me decide what to do. She encourages me to have better health. |
| I have been a patient of this doctor for many years. We have become friends. I trust his judgment and his expertise and that he has my best interests at heart. |
| Thorough describes this physician. It amazes me how she remembers conversations we’ve had during prior visits. She is full of compassion, understanding and patience. She always asks about my family. |
| I visited this doctor for the past 25 years, he was down to earth, realistic on expectations and knowledgeable for both general practitioners and female issues as I aged. |
| kind and thoughtful concern in regard to my health and providing me with excellent medical and physical care. I am most grateful for your kindness whenever I see you. |
| They were very kind and empathetic to my situation. I was certainly afraid of being in the hospital at that time and they made my stay much less terrifying. |
| The doctor is very kind and very understanding my situation of the treatment. He really very calm and hear my all Sad situation in my life and treat me. |
| The doctor I’m referring to retired a couple of years ago, but was an exceptional doctor. He took time to listen and was always polite and professional. |
| MY EXPERIENCE WITH THE DOCTOR WAS TOO GOOD. HE WAS SO PASSIONATE. HE UNDERSTOOD MY PROBLEMS AND THE WAY HE COMMUNICATE WITH PATIENTS WAS ALSO SO GOOD |
| My Family Doctor Very Good & Comfortable Person. My Family Health issue any One coming Time I am Fist get To my family Doctor Appointment Order . |
| Better Experience for Doctor. Because I am very fear so I am Not Going Mostly Hospital. But Now Going To Hospital . Its ok Very Comfortable. |
| The doctor is a helping human being the very excellent human being and patients are care very super but he is great man of the doctor |
| I liked the guy from the start of our relationship, as he seemed like a good old southern boy, with a genuine smile and real laugh. |
| My doctor is such a kind heart person some time he will not ask fee to me when i am not in good financial conditions. |
| My Doctor is very kindhearted person. Even he cannot asked me fees when I have not enough money. So I like him very much. |
| Treating the patients very honestly and take care of the patient health and giving valuable tips to improve our daily life health care. |
| The doctor could take care on my health and treated me as her family member. Apart from money she spread love on patients |
| My doctor is kind heart some time he will not ask fee to me when i am not good in a financial conditions. |
| The services that i receive from excellent.Wonderful experience, Great medical office, Wonderful and great experience as a first timer.This practice is terrific. |
| The services that i receive from excellent Doctor. wonderful experience,great experience as first timer, this practice is terrific and Great medical office. |
| My primary doctor is very caring and honest. I like him and he always gives me his undivided attention with my health. |
| My doctors is kind hear some time he will not ask fee to me when I am not in a financial conditions |
| My Family health Issue Any one coming Time Always going To My family Doctor. That Person very comfortable & Good Person |
| THIS DOCTOR TREATMENT IS REALLY GOOD. BECAUSE MY PROBLEMS IS COMPLETELY CURE.SO I SUGGESTION FOR OUR FAMILY AND FRIENDS. |
| kind and thoughtful concern in regard to my child health and providing me with excellent medical and physical care. |
| From the beginning when I started to see my doctor, I noticed he always smiled and shook my hand. |
| SHE TREAT ME VERY HUMBLE AND GOOD. SHE RESPECT ME A LOT. I LIKE THE DOCTOR AND HER TREATMENT. |
| This doctor always took time to listen to my concerns; she was also non-judgmental. She explained treatment very well. |
| I EXPERIENCE A PROUDNESSN REGARDING THE DOCTOR BECAUSE HE IS SUCH A TALENTED PERSON I WOULD EVER MET |
| This doctor is meticulous in prescribing not only a suitable drug but the best drug for my condition |
| Yes he consult me calmly and he speak very friendly and he talk and care about my health |
| My Family doctor Very Comfortable Person Because Mostly Patients Going to the Hospital Comfortable place & Vibes |
| he is so kind and down to earth and gives exact medications and advices without any exaduration. |
| Lots of Thanks for such an excellent treatment. most importantly he is a a great human.. |
| This Doctor seems to know what specialist to send me to if I have a problem. |
| THIS JOURNAY VERY GOOD EXPERIENCE FOR ME AND I AM SO PROUD ABOUT THAT DOCTOR |
| He is good person and take care of all the patient good human being |
| He spends extra time with me and make sure all my questions are answered |
| Great medical office, wonderful and warm experience from start to finish. ... |
| They taking care of patience healthcare and give best treatment to everyone. |
| esse médico me atendeu muito bem com atenção e dedicacão e respeito |
| THE DOCTOR IS VERY GOOD AND HE USE TO GIVE GOOD ADVICE |
| reat medical office, wonderful and warm experience from start to finish. |
| If you go to them, the disease will get better soon |
| This doctor is one of the best that have treated me |
| friend of docter and helping hand and most sweet person |
| they treat all are equal and they are very humble |
| She was very friendly and she handled me very well. |
| I like him very much for the above mentioned qualities. |
| She always listens to my concerns and gives informed advice. |
| The doctor is very good person and good treatment. |
| The taplets he gives is a low dose taplets. |
| he was soo carying and good at his work |
| THE FEES COLLOTIONS IS OUR PATIENT STIUTAIONS UNDERSTANDING HER. |
| He treating his patience very empathy and respect. |
| He is very open minded and very humble |
| THE DOCTOR IS EXCELENT TREAMENT AND CARRING PETIEANT, |
| She is very humble and very loyal |
| the best person i have ever met |
| Great experience as a first timer |
| the best and the caring person |
| She is very humble and honest |
| She is very humble and honest |
| The fees him will be less |
| He seemed to be genuine. |
| He is a good doctor. |
| VERY KNOWLEDGABLE AND KINDFUL PERSON |
| He will look patiently |
| he is treated good |
| She is good doctor |
| I AM VERY PLEASED |
| Doctor Exceptionally good. |

## Category 3: Respondents’ description of the exceptionally good doctor

| **Respondents’ description of the exceptionally good doctor** |
| --- |
| A colleague at work went to him for gynecological surgery and was impressed with his willingness to treat her when others refused. Her surgery went flawlessly and she recommended that I see him to get another opinion, as she had. I made my appointment and brought my husband along as a second set of ears. The doctor walked in with his hand extended and making direct eye contact with both of us. He introduced himself by saying "Hi, I’m Michael. How can I help you?". We had a lengthy conversation, about one hour, where he said that my condition was challenging but he believed he could do what I need laparoscopically, avoiding the major surgery and lengthy recovery I was told by six previous doctors that I needed. He met with me once a week for 8 weeks to personally treat me so that my body would be ready for the surgery. When I arrived at the hospital on the day of my surgery he was there waiting for me, not in the OR, but in my room to answer any questions - he didn’t allow the nursing staff or residents to reassure me, he did that himself.  I was prepped for surgery and walked to the OR, he told me later he thinks people are more comfortable when they feel somewhat in control of the situation, and he was waiting for me in his scrubs outside the OR doors to walk me into the OR himself. He left me only to get prepared for the surgery, came into the OR and asked me what my favorite music was. I told him and he directed someone to please play it for him, he then told me it was his favorite too and then waved at me and said "Good Night". I saw him after my surgery when he was there to make sure I was comfortable, checked if I needed any pain meds, and then said he’d see me in the morning before he’d discharge me.  He was at my bedside at 7am the next morning to make sure I was ready to go home, went over the instructions for my follow-up care personally, and then said to behave and not think I didn’t have surgery because I felt fine. He explained what was necessary to make sure I didn’t do anything to slow the healing, even though I couldn’t feel any pain I had still had surgery and internally I had to heal. Then, when he was satisfied that I had heard him and understood what I was to do/not to do he told me he’d call the next day to check on me, which he did. I was back at work in 10 days rather than the 6 weeks that the other doctors said I’d need for a totally different procedure which I was glad I avoided.  I have since recommended many friends, family members, and neighbors to him. One coworker, who was terrified of developing ovarian cancer asked me for his name. She said her doctor, and several others, refused to do a hysterectomy which she requested due to her age - she was 38 and had one adopted son. Her mom had just died from second site ovarian cancer, her first site was breast cancer, and my coworker had just completed chemotherapy for breast cancer. She was terrified for her son’s and her future. I called my doctor, I had an appointment for a follow-up the next day, and asked if he would fit her in and talk to her. He said "absolutely, bring her along". He spent an hour with her and agreed that this was the best thing for her to do, if only for her mental health. She was delighted, relieved, and scheduled her surgery with him. To this day she is alive, well and cancer free. This doctor is a very successful department Chair in a very prestigious NYC hospital. At his level you usually find doctors to be rushed and impersonal. Not my doctor. |
| She was very patient in listening and also communicating with me about my father. She gave me a clear understanding of what the situation was and what to look for in the future. My dad was being treated in a hospital and she was overseeing things as she was a friend of my friend. Doctors usually have massive egos that is sometimes not earned by them. They don’t like being asked questions. They have their juniors to block these types of communication. They also follow protocols blindly most of the time without using their common sense or really observing the patients. This neurologist was really exceptional because she liked discussing things and was open to questions. She also had the acumen to change course in treatment as the patient showed different symptoms or improved without just sticking to "protocols".  I had a really bad experience with incompetent doctors so this was really refreshing to see. It was not about money at all as I didn’t pay her much. She had a sense of humanity and a genuine sense of caring nature. It made me feel secure and I could explore information myself and consult her. Some of the doctors, especially during and post corona have lost their focus and their ethics. They do not question or even care to question anything. This is very unscientific and makes me nervous for other people as well. A doctor without common sense can be dangerous. They ought to have discipline and dedication regardless of the circumstances. They can’t give up easily and shouldn’t, just because there is some pandemic or the statistics suggests that humans die after all. Doctors should be inventive, treat people as humans who are owed dignity and have a right to live. They should be updated with technology.  I saw hospitals that had really low level sensors and gadgets. This is embarrassing. Doctors who mundanely do duty just to make money can never be good doctors. They should try and strive to make the medical industry better. They should have enough courage to question things. It is not just about cramming antiquated books. Right now, I am thoroughly disappointed with the general set of doctors we have. Some of them are downright immoral and bereft of any guilt. A great doctor would do justice to the oath they took. |
| After having my right hip replaced by an exceptionally BAD doctor resulting in permanent sciatic nerve damage, I was quite scared and apprehensive when, years later, I needed my left hip replaced. My primary care physician recommended that I go to an orthopedic practice in a nearby hospital, and based on online reviews I chose Dr. H. I was frank with him about how the other hip operation had permanently altered my life with terrible repercussions. He listened and I felt he truly understood what had happened to me. He was honest with me and said in his years of practice he had performed one operation which resulted in sciatic nerve damage, and I was surprised that he revealed that. I was very overweight at the time and my health insurance required me to get to a certain BMI before it would pay for elective surgery, so I saw Dr. H. several times over a period of months while I worked on losing weight. He was always encouraging,  I eventually lost 68 pounds and the operation was authorized. Actually, when I went in for the last weigh-in, I was 3 pounds short of my goal, but he told me I’d made it. His attitude on every visit was kind and personal. Despite being a busy surgeon, he allotted as much time as I wanted to visits, explained everything carefully, answered all my questions, and never acted like he was too important to bother with me. After the operation I went to a rehab and every time a nurse changed my dressing they would remark how beautiful the incision was, clean and precise. Every time I mentioned that he was my surgeon, nurse, therapists, and other doctors gushed effusively about how great he is and what a wonderful reputation he has. On my post-surgical visit I told him that, and he blushed like a schoolboy. My hip healed with minimal pain, and has been perfect ever since. In short, Dr. H is a highly-skilled, well-respected, busy surgeon, but he treated me extremely well. |
| I’ve had foot problems my entire life. I was pigeon-toed and flat-footed as a child. Of course this carried through my adult life. I’ve spent much time and money on shoe inserts and other orthopedic doctors. I had one foot surgeon actually tell me I had "ugly monster feet". He was trying to joke, but it brought me to tears after having a lifetime of foot issues. It was the last time I saw him. I put up with the pain for years until I couldn’t stand it any further. I asked my primary care doctor for a referral and he referred me to this surgeon whose office is out of town, in a larger city about 40 miles away. I immediately liked this guy. They did x-rays and he explained what was going on with my feet. One was quite severe and he said he could fix it. He said it would be a challenge, but he could fix it. He didn’t tell me my feet were monstrous. He was caring and wanted to help.  As a result, I had my first surgery in September 2016 on my right foot. He built an arch, shifted my heel, and grafted a bone in it. It was 8 weeks non-weight bearing, then therapy to learn to walk on my new foot. My left foot wasn’t as painful so I put that surgery off until this year as it was getting progressively worse. On March 28, 2022 I had my left foot operated on and he did the same thing, but this time there was no bone graft needed. I’m currently half way through my 8 weeks of non-weight bearing. I started therapy this past week to help move my toes in a non-weight bearing way. It’s a long process. I’ve said many times to many people that this man changed my life. I believe he’s the top of his field and would recommend him in a heartbeat. |
| I needed a hernia surgery (I had an obvious bump above my belly-button, and my self-diagnosis was confirmed by my Primary Care Physician). I searched online for hernia-surgery specialists near where I was living at the time. I found the doctor who performed my surgery via Google. I saw that he was written up in a local magazine as being the best overall doctor in the area (granted, he was 40 minutes away from me). I could see from my online research that he had many years of experience performing ABDOMINAL hernia surgeries specifically, so I knew that he was knowledgeable and had real-world experience. I didn’t want him to come across something he had never seen before while operating on me and then not know what to do! I had one consultation with him for him to look at my abdomen, and he confirmed that I should get surgery.  He was very friendly and smiled a lot. He answered my questions with just the right amount of detail. His assistant was also friendly. Right before the operation, as I was getting wheeled into the OR, he was reassuring (as were his assistants). After the operation, when I was awake for 10 minutes, he came to check up on me. He continued to smile and helped me to get out of bed and walk for a minute or so. He said that the operation took longer than he thought because I had a lot of fatty abdominal tissue that he needed to excise or tuck back in place. I liked hearing this, because it meant that he cared enough about doing a good job that he took the extra time needed to do so. |
| On my way to work in the morning a year or so ago, my phone lit up with an unknown number and I dismissed it several times. When I did answer it was my mother’s doctor. He was calling to tell me that if I wanted to see my mother before she passed, I needed to come now. This is during the height of the pandemic. My mother is in isolation. She can only see us thru the glass. I met with and spoke to the doctor for the first time. He told me how he has been seeing my mother for several years and I thanked him and told him I appreciated him.  I added that I would like to see her, too, one more time before she passes. That was not in the protocol for the hospital and this ward. I then heard him in the background on the phone and directing nurses and his entire focus was arranging a way for us to be in the room with her and hold her. It required special masks not available on that floor and gowns and gloves and he made it happen. We were allowed into her room and I had a chance to speak with her moments before she passed. "No regrets, no regrets." She responded the same. For me and my family this was the most precious moment that I could have had and I will be forever indebted to that doctor. If not for this doctor, this man, we would have had to watch her pass thru a window. There would have been no touch and no conversation. He’s on my Christmas card list, just so you know. |
| My experience with an exceptionally good doctor was many years ago when I was suffering from extreme contact dermatitis on my hands. I was becoming increasingly debilitated and tried every remedy without relief. During a routine gyno exam, she saw my hands and referred me to a dermatologist. I completed the usual paperwork, then the elderly doctor walked in and sat down next to me. He peered skeptically over his spectacles, wiggled his white whiskered moustache, looked briefly at my hands, then starting reviewing my history.  I was preparing to answer standard questions and receive routine prescriptions but was surprised when the doctor began slowly asking different kinds of probing questions that weren’t even on the history. He delved into my lifestyle, personality, environment, life stage and several other areas that seemed fairly irrelevant. As he gathered more information, he began to construct a profile of circumstances that had contributed to the condition, and didn’t just right away start to treat the condition itself. He had a specific treatment regimen but more importantly, an overall strategy for me to implement on an ongoing basis. That day, I gained not only supportive and responsive care, but more importanty, tools and pearls of wisdom from his keen insights and guidance. The results not only led to the swift, total cure of my hands but provided me with deeper insights into myself and have stayed with me throughout my life, helping me to avoid many other skin eruptions, discomfort and pain. |
| I met Dr. Cooper 28 years ago, This woman is a exceptional doctor. She had the training in oncology and knew the latest ways to tackle my disease. Se works still to this day at the same learning teaching hospital, Being affiliated with this hospital gave way to the latest in her field and was and is accredited with the american cancer society and trials. Se is exceptional for her expertise and knowledge. She is also excpetional as she works with a team of dcotors, She is compassionate with her patients and with her field of medicine. She is kind and thoughtful. I can remember her holding my hand and encouraging me. Always giving me hope and comfort. Made sure I got the care I needed. It wasnt easy back then and without her care I wouldnt be here today I am quite sure.  Proud to say I am in remission 28 years for leukemia, She performed my third bone marrow transpamnt using stem cells from my brother which was new back then. I had my previous 2 transplants done at a different hospital and they didnt work as they didnt know what needed done. I left their due to an arrogant doctor who told me I am the doctor and I am going to do what I want to do. So dont be an arrogant doctor is my advise to you also. |
| I went to this particular doctor after an ER visit for a dislocated finger discovered that I had extremely high blood pressure. I needed a GP that accepted Medicare, so I called around until I found one. In consideration of my age at the time (65) and the fact that I had not seen a doctor in years, she went about diagnosing and treating my condition systematically, looking for possible causes and effects. She took X-rays and an ultrasound and discovered I had an impacted kidney stone that had caused one of my kidneys to swell. In short order, over the next three months, she referred me to a urologist (who performed surgery on the stone), ophthalmologist, and neurologist to determine how, if at all, the hypertension had adversely affected other organs in my body. Since I had never had a colonoscopy, she referred me to a gastroenterologist for that procedure;  I have also seen a cardiologist (for heartbeat irregularities), a nephrologist (for concerns about kidney disease, ruled out for the moment), and a dermatologist (I needed plastic surgery on a large patch of basal cell carcinoma on my face). She continues to monitor me closely. She is warm and compassionate, communicates well, and doesn’t leave a stone unturned when it comes to my health. |
| I had my regular check-ups and during one of those, it emerged that there was something wrong with my left breast, the ultrasound technician said that I had an adenoma. Since I knew nothing about it, I went to my GP to ask some more questions but, she didn’t took her time to explain the issue and discuss the topic, she was a sloppy doctor and I fired her ass right after the episode.  I went to this specialist, a friend of mine told me about him, and I was extremely worried and scared, didn’t know what to do, what to think. He was kind and nice, he took the time to explain the matter to me with simple words, he showed me he cared about me, he was human, less of a doctor and more like a caring friend. My GP triggered my Anxiety while he shut it off. Some people should be banned from attending med school because they don’t fit, they are after the money. Some other should be rewarded because they not only share their knowledge, they treat you like a human being, not just a cash machine. |
| This doctor was the pediatrician for my children. He was enormously kind and exceptionally intelligent. My daughter had a couple of very rare problems when she was a toddler. One of them was a cholesteatoma in her inner ear that required major surgery, and if left untreated could have resulted in her becoming deaf or worse case scenario death. He was checking her ear and said,"I’m not sure what I’m seeing. Let’s make an appointment with an ENT. He’ll have a more powerful scope to see in there." So, he actually referred her to someone else. And for that I am thankful. The other one was bladder reflux. And we worked together on that was also was referred for the necessary tests. Through all of this is was calm and pleasant and cheerful. He was wonderful and made daughter loved him, even though she went through a lot. All of this required bi-weekly checks, and he only took whatever my insurance would pay. I did not have to pay anything out of pocket. I will always be appreciative of how he took care and seemed to love my children! |
| I had fallen and hurt my wrist. The next day when the wrist was swollen I went to emergency. At the time I was pretty well know bar owner so I was treated very well by the staff and put into a emergency room. I think it was the cardiac unit. The attending doctor, female was also fantastic and would be the doctor specific to this study if not for the resident who actually set my wrist. This guy was as I said a resident and the doctor he worked under was a jerk. (I believe the jerk was the attending bone guy but he was terrible). In any case this resident showed sympathy for my injury, explained more the well what he was going to do. We had fun (including the ward doctor) while setting the wrist. I won’t go through all that was done lets just say they all were impressed with my pain tolerance during the procedures. All in all it was a fine experience getting my wrist set and those two doctors were perfect! |
| This doctor was a young man (not much older than myself) who had previously treated both my mother and brother before we ever met. I had developed a rather nasty case of bacterial cellulitis owing to a spider bite while I was outdoors camping in the summer time. By the time I had returned from my trip three days later, I was in immense pain and could barely walk as my feet were too swollen to fit my shoes. Dr. Banks was very professional, he did not make me wait for longer than a few minutes. While examining me, he told stories of his own mishaps while outdoors which calmed me down considerably. He explained the mechanism by which the infection had spread (at this point I realized how serious the bite had actually been) but indicated it was no great cause of concern. I was given a prescription antibiotic and instructed to stay off my feet for the next couple days. I have continued visiting him ever since - as he is now my GP. |
| I didn’t have a physician (my longtime family doctor had just retired) but I suspected I had bronchitis and/or pneumonia and searched for a doctor in my insurance provider’s network. I picked one who was close to my home and right away I could tell she was exceptional. We instantly developed a good rapport, she was easy to talk to and took her time to field all my questions. She was very patient and understanding through all my coughing! She seemed to genuinely care about making me well. She even called me at home later to see how I was feeling. Just before my follow-up visit, my mom passed away. I kept my appointment even though I had just suffered a significant loss the day before. She sat with me for an hour while I cried and grieved. When I left her office, I felt cared for. She was a rare breed, the opposite from other experiences I’d had before with doctors. |
| I had a really bed springboard accident and broke my neck, shoulder, and wrenched my ribs away from my spine. I went to doctors for ages and each one told me my pain was in my head and nothing was wrong with me. Turned out some of my ribs had dislocated from the spine, and with his help, we were able to relax the muscles and after working with me for a while they finally went back in place. I can’t even tell you first, what a relief it was to find a doctor that didn’t make me feel I was just trying to get meds, and then helped solve the problem that the other doctors never bothered to find,. The relief from the pain was truly profound when the problem was finally fixed, and even though I have pain from the arthritis these breaks caused, it is nothing like the pain I felt then with those ribs being misplaced. |
| I was very pleased with my experience at Doctors Care. I can be a hard critic of emergency care places and the people employed by them. Combine that attitude with an innate crankiness when I’m not feeling at all well and the result can be much less than pleasant. This time (My first time at Doctors Care) was a world away from previous experiences elsewhere. It was good to be treated with professionalism and care. When I say the doctor and all the other employees of the Dorchester Doctors Care made me feel that I was in safe hands and even made me smile when I left, then you can believe they are the best. I am truly grateful and won’t get that awful feeling of dread the next time I get sick and am unable to see my own physician. I appreciated their follow-up and willingness to answer questions also. A big thank you to all of them.” |
| I first met the cardiologist when he saw my husband for the first time for chest pain. While in the exam room, my husband went into cardiac arrest and had to be revived several times. The doctor went in the ambulance with my husband to the hospital to keep him stable. The hospital was 40 miles away so it was not a short trip. He stayed with my husband for most of the day until he was stable. His office had patients waiting to be seen after my husband and everyone was sent home, as well as other patients on the schedule to be seen that day. This man did an extraordinary thing by taking care of my husband at the expense of losing money that day by not seeing his patient load. You do not find many like him. My family and I became his patients when we needed any procedures or tests related to the heart. |
| I suffered from kidney stones all of my adult life and usually had to pass them. The last time it they were problematic. Twice before I went through sonic wave, and in each time the procedure failed. The doctor decided instead of medicine they should just be removed because of their size. Extremely nervous because out of the two choices he seemed to bank on the one that was the most invasive. He actually explained the procedure extremely well, was honest about the any discomfort and recovery time while not minimizing my anxiety about an incision in my back. It didn’t make any less nervous but I knew understood logically why it was the better choice. Having dealt with kidney stones for so long, I had always heard of the procedure but finally having someone take the time and answer all of questions meant I could finally have the best treatment that could handle my particular situation. |
| My doctor was recommended to me by a friend. I have seen her for several years now, but what I really noticed when I first met her was how warm and kind she is. I’ve had many doctors who were cold or seemed rushed and annoyed when I asked too many question. This doctor makes me feel like I matter. She listens to what I have to say and takes the time to answer my questions. She makes me feel like I matter. She also seems reasonable. For instance, my blood pressure is almost always higher than normal when I go to a doctor. She knows this and will take it again later in the appointment when this happens. Inevitably, my reading will be much better after I’ve had a chance to relax. She always seems to be on time and I have never waited more than a few minutes after arriving. |
| For 20 yrs, I went to doctors with same symptoms. They would check calcium and it was sky high. Check parathyroid levels and it was normal and refuse to explore further. Repeatedly referred to therapy. I finally ended up with kidney stones from sky high calcium. Urologist said same thing, high calcium but normal parathyroid. Go to therapy. When I applied for aid to pay for surgery, hospital sent me to a brand new young and foreign born gen practitioner. She ordered the expensive test and found the largest parathyroid tumor ever seen by surgeon,. endocrinologist, imaging specialists. I’d had the tumor the whole time and was one of tiny percent of cases with sky high calcium and high hormone that test registered as normal. It was because she was a she, young and foreign that she bucked the system to track down real problem |
| Sadly after this great experience I was placed with the jerkie attending for additional x-rays and release. What an ass... this guy had an orderly wheel me up to x-ray while he went on a jaunt way ahead of us stopping and talking to people along the way. (It was clear the orderly didn’t like him much). Then after I went into the x-ray room he placed my arm on the table then he left the area (without explanation) and I could hear him talking to some female about them going out to dinner. After about 45 minutes another person came in and took my x-rays. All the while the jerk never said a word to me, rather he guffawed around with the people in the x-ray booth. Later he said to me, well that’s the best we could do... that was it. |
| The doctor was a specialist pediatric surgeon who made the right calls for my 24 week old micro preemie triplet baby to be delivered since the other two triplets passed and a forced delivery would inevitably happen. She made the right call to give the steroid injections for my baby to fully develop his lungs as much as possible before being born since he was only 24 weeks old when he was born and babies are generally still developing inside the womb during this time. A week later he was delivered and he came out very strong and bigger than they all expected. My doctor also made the decision to do a certain c-section to deliver him. She was so spectacular, compassionate, caring and understanding with me through the whole journey. She really is a special doctor. |
| Dr. N is good about connecting me to other specialists when needed. He has sensitively counseled me about the need to lose weight without fat shaming me. He shares with me some of his family’s struggles with weight and the treatments that have worked for them. We commiserate over the care of aging parents, vaccine resistant friends, and crazy local politicians. I appreciate him sharing himself with me and it makes me trust him more. I never feel like he is pushing me to do anything, just making suggestions, and asking for my input. I like being treated like an adult and a collaborator in my healthcare. The only problem I see with Dr. N is that he is about my age, and will likely retire one day soon. That will be a sad day for me. |
| The first time I went to see this doctor, I sat in his waiting room for 45 minutes. Then they took me back to the exam room and I waited another 30 minutes. I got up and left at that point. That afternoon, Dr. N called me and apologized profusely. I decided to come back and I found a doctor who was an exceptionally good listener. (I think he lost track of time that day because he is a good listener and doesn’t blow through exams as many other doctors have with me in the past.) Dr. N now has someone to transcribe his notes for him and his office staff seem to be much better at setting appointment times realistically. Depending on what’s going on, the average time he spends with me is 20-30 minutes. |
| 25 years ago I had tri-geminal neuralgia. I first tried having it treated with carbetol, but it only worked slightly. Finally I asked for a referral to a surgeon. I went and he explained about a new type of surgery that had avery high success rate so far of fixing the problem but there had only been about 200 surgeries performed world wide at that time and only in two places but he new he could do it. He was the top neurosurgeon in the state. Anyway he gave me tons of liturature and explained in all in several follow up visits and finally I had the surgery. 100% successful...His knowledge and communication skills were what convinced me to have the surgery rather than cutting the nerve as was done up to that time. |
| I met her 30 years ago. My regular physician was too busy to get in to see so I took an appointment with the "new kid" at my group of doctors. In the first appointment she started listening and helping me with multiple issues I could never get help with. I still remember her listening to my symptoms and educating me on what I needed to do to feel better. Getting me to take the medicine that would eventually make it easier for me to breathe, sleep and thrive. No one had ever helped me so much. She is an expert communicator, detective and so very caring. She continues to take good care of me after all these years I trust her completely. |
| After many years of suffering extremely debilitating headaches, nausea, vision loss and several other symptoms, I was again stricken with an episode. I had seen many doctors previously that dismissed my concerns and complaints as a migraine condition without further investigation of any kind. I was forced to continue to try to find a resolution for this problem because I was so debilitated. When I went to my first visit with this perceptional doctor he took my concerns seriously, listened to my complaints fully and did thorough testing. Through this testing and investigation of my symptoms he was able to promptly diagnose my medical condition. After years of suffering I finally had an answer, which saved my life and relieved my suffering. |
| My leg was blistering and swollen up due to the heat of an infection I had. The doctor listened to me and looked at my leg thoroughly. He chose the course of treatment he thought best. He explained it in full to me and I agreed. It meant having 2 IV drips in me at the same time. I had a massive dose of both Penicillin and Fusidic Acid. He drew around my infection and said if it went out of the line he would have to put me in isolation. I had not to worry though because he too would be in isolation with me and he stayed with me for an hour until the drugs started to work |
| I was referred by my primary physician for a hysterectomy. Due to the reason for the surgery, when he recommended traditional abdominal surgery, I inquired about laparoscopic surgery as an alternative. He explained that he was not trained in the procedure and recommended that I proceed with abdominal surgery. I continued the discussion with him but, ultimately, scheduled the surgery. About a week later I received a call from him. He explained he considered my request, contacted an experienced surgeon, reviewed my case and, if I agreed, he wanted to proceed with assisting the experienced surgeon with the laparoscopic surgery. He indicated this would provide him with the opportunity to learn and assure I was treated by a seasoned surgeon. |
| He reiterated the post-surgery care that I needed to adhere to, and he said that he’d call me in two weeks. He did call me 2 weeks later, and when I expressed concerns about my mesh possibly moving out of place in the future, he said that he put tack welds onto the repair every centimeter, indicating that the repair was going to stay in place. We ended the conversation in a friendly manner. I think maybe one of his assistants called to check up on me several months later, which was nice. This surgeon possessed all 5 qualities which I expounded on at the beginning of this survey: friendly, willing to take time, not scolding, knowledgeable, good follow-up. |
| I was suffering from severe stomach pain. One of my friends suggested to me an exceptionally good doctor. The doctor was a good listener. He gave me an injection to relieve my pain as soon as I arrived. He diagnosed me and prescribed medicine. He advised me to eat mostly home-cooked meals in the summer and drink filtered water only. He told me to call him if I need him again. He was very down to earth. He joked about a few things to make me feel better. I was relieved. I could feel he was genuinely concerned about treating me and not just charging me a hefty bill. |
| This doctor was just so amazing. He was so human and reachable in a real way, not just talking in terms of medical knowledge. He was interested in what was going on in my life. He was an osteopathic doctor, and so he was in tune with other forms of medicine as well. I think what I liked about him the most was his acceptance, his non judgemental way of approach and his caring attitude. I mean, he REALLY cared. He made me feel like I was just the most important patient he had, and I think that made a big difference in how I faced my medical challenges. |
| The doctor that I had was named Matthew Wise. He was an exceptional doctor because he was very kind to me during a rough patch in my life where I was very concerned with my mental stability. He gave me ways that I could improve my health and did things that no other doctor did during my visit. He took the time to listen to me, placing a stool next to me and listening to every single thing I had to say. He wouldn’t even let me leave until I was completely looked at. I think every doctor should be like him. Even his telehealth visits are exceptional. |
| I was having multiple issues occuring approximatly 10 years ago. I had gone to 4 other Doctors for issues due to itching on trunk, fatigue and generale malisse. This new Doctor ordered a Hep-C blood test where the previous for had only ordered generic blood tests. I had the Hep-C that showed treated and I am "cured" now. This Doctor also helped me get on SSDI and suggested outside medical help programs for me. He went above and beyond and has helped me through many other issues since. His name is Dr. Mohammad Ahmad @ Assension Hospital, Racine, Wi |
| I had sores that would not heal. I wernt to my regular doctor and he just gave me some antibiotics that I should take without doing any testing. I was still bothered , so I got a second opinion from a new doctor. He took one look at tyhe sores and diagnosed it right away. He took a sample to have it tested to make sure and prescribed the correct antibiotics. The pain and sores went away very quickly. I have been going to him ever since and is a pleasure to have him as my personal physician |
| I was having difficulty seeing in the office, often having a glare on my computer screen. I wanted to wear sunglasses all the time. He found a difficult to diagnose type of cataract. Following surgery I not only no longer had this issue, I had near perfect vision. Moreover, my Mom was a patient of his fo decades with many vision problems. When she was on hospice, they declined to treat the eye which caused her great discomfort. I went to him and he was able to give me an RX and detailed instructions to alleviate her discomfort. |
| I found a lump in my breast. Was referred to this doctor. Not too long a wait for the appointment, and not too long a wait in the office at time of appointment. Doctor was matter of fact, kind and thorough. After a discussion of my family history, he examined me and recommended a breast biopsy. The lump was a benign tumor. He knew breast cancer ran in my family. After the biopsy I woke up and he was right there, saying "it’s benign". Followups every six months for several years with various tests. He was wonderful. |
| But what made them most exceptional was that they actively advocated for me with other health care providers. It was especially welcome in dealing with neurologists. On one occasion, they were forwarded a copy of a brain MRI that had been conducted by another doctor. That doctor indicated that there was nothing to be concerned about in the findings. This doctor looked at the report and pointed out that there were, in fact, a number of abnormal and concerning findings in it. They actively sought a referral to a different neurologist and flagged those concerns with them. |
| I was experiencing blood clots and no one could figure out why. I was also having extreme breakthrough bleeding and my original gynecologist scheduled surgery but opted out of my insurance provider network. My primary care doctor sent me to a new gynecologist and she was thorough and went through every test and record and I generated in the past year and also wanted to know why my pap smear was irregular - which was news to me. She found several things wrong and 2 surgeries later I was good as new and no problems since. |
| He was assigned to me as part of medical operation that involved the rupture of my intestine, which was a medical emergency. My original primary care doctor did not treat at the hospital I was taken to. From the time I met him he showed me how much he was interested in my care and always willing to help in any way possible. As, a result of this, I have been going to him as, my primary care doctor, for the last eight years. He is not only a good doctor, but an exceptional person. |
| Some time ago a knot was increased in the food pipe of my wife, she had trouble in swallowing food. An endoscopy and any tests showed she had cancer. A friend of my son who was treated by this doctor told us about this doctor. We went to this doctor who patiently hear us, examined my wife. And ask for some laboratory test. After two days we went to that doctor with that report. After carefully going through the reports he assured us that within some period my wife will be cured. |
| I had a bad case of pneumonia. I could barely make it to the phone to call my ex-wife. My doctor took me in immediately without having to wait in the waiting room. He told me he was going to give me a shot of something he called Rosephrine (I am guessing at the spelling). He said it was previously uses to treat horses and had been approved for human use. I made no objections because I would have done anything. I could hardly breathe and felt like I might die. |
| This doctor really listened to me. She spent an hour with me, going over and explaining exactly what was going on with my heart. Additionally, she prescribed tests which uncovered a condition my former cardiologist missed entirely, because he was so dismissive of my symptoms. The new doctor’s physician assistant and cardiology nurse are on hand all the time to provide advice. They are really responsive when I call. They also set me up with a good nutritionist. They treat the whole patient, not just one symptom. |
| This doctor was my doctor when I was pregnant. He delivered my baby. I was young, ashamed and embarrassed at the time and he really was kind and gentle with me and took the time to make me feel comfortable on all of my visits. He took the time to thoroughly answer all of my questions without making me feel foolish. He was able to supply me with other resources that could help me also. He made me feel comfortable during a very scary experience. |
| This doctor became my oncologist after I was diagnosed and operated on for ovarian and endometrial cancer. Cancer is a very scary word, but he was such a cheerleader in helping me develop a "can-do" attitude. I visited him many times for check-ups and chemo infusions. Each time, he would talk to me about my concerns, how I’m coping and offer different strategies to keep myself positive. Every time, he made me feel as if I mattered as a human, not just as a patient! |
| I was referred to this surgeon by my GP when I needed to have my thyroid removed. She was very clear in explaining (with diagrams) what she could do and what my life would be like afterward. She told me without bragging that she is very good at what she does and she would do an excellent job for me. Everyone I met in the hospital from admitting to the nurses made a point of telling me what a good doctor she is. |
| I had pain in my legs when I walked for any amount of time. It became so bad that I had to quit my daily walks. He suspected that one of the veins in my leg had closed and the lack of blood flow was causing my muscles to be oxygen starved. He scheduled a ultrasound test which would point out the area and, when it was determined that he was correct, he scheduled me for surgery and had a stent put in. |
| I first met Dr. S. while working as a floor nurse in an oncology unit in my city. I noticed that he was the only doctor who routinely called (at 7:15 am) wanting to know how his patients were doing. He would ask for specific lab work and was always polite and caring. It was over many years that I learned his patients seemed to get well and discharged faster than other oncologist’s patients. His patients also had a higher cure rate. |
| I was operated on by this doctor after I suffered a broken neck. He gave me a 50/50 chance of being a quadriplegic or dying from the injuries and surgery. I felt a peace and calmness coming from him and put myself in his hands. After 9 long hours the surgery was complete and I was alive and completely mobile. I healed well and after more than 20 years I am still fully functioning. I credit it all to this fine neurosurgeon. |
| I was referred to him from my own doctor when I was younger. I needed a surgical procedure and it was rare and he was recommended. He was on the staff at UC medical center in San Francisco. He was great, I was younger and appreciated his calmness. He did the surgery and everything went well. I was in the hospital for 10 days after that and he or his students checked on me regularly. I had follow up visits with him. |
| His final surgery, 5/6/2021, with a west coast surgeon for 2 iliac aneurysms, was a horror with heavy bleeding, hemorrhagic shock, etc. The clotting factor led to incredible organ damage. He spent nine weeks in the ICU, 17 trips into the OR, before he finally caught the superbug and pulled the plug. By the time he died, the ICU doctor told me only his brain and his liver were functioning on their own, everything else was run by machine or medication. |
| I have small fiber neruopathy and have seen about 10 different doctors and have had several diagnosis but all were not correct and made no sense. This doctor helped me and performed multiple tests and surgeries to confirm whats wrong. Now this has been about a 5 year endeavor and after only 1 year he was able to diagnose me and its stuck and I have been receiving care and he’s really helped me more than I could’ve ever imagined. |
| A few years back I showed up to the ER as a patient in need of emergency surgery with little to no ability to pay (and I looked the part) but the doctor that was assigned to be my surgeon treated me with the upmost respect and with a level of genuine caring that I was totally not expecting. His soothing presence and calming demeanor transformed what should have been a disastrous ordeal into only a minor medical setback. |
| My regular gynecologist was on leave, so this doctor was available to see me for my yearly exam. She talked to me as a human being, and I genuinely felt that rather than the professional demeanor that all doctors have to put on. Also, I was confident of her skills and knowledge because she is about my age -- 65 -- and she has been in practice a long time. I just was really impressed with her. |
| Some years later my mother was dx with colon cancer and I mentioned she should go to Dr. S. He cured my mother and she has been cancer free for over 15 years. I have never met a more exceptional doctor. I saw daily how he cared about all of his patients and seemed to be born for what he does. He is also very involved in our community. He is one of a kind. |
| He was chairman of the OB/Gyn department of a major teaching hospital. I worked for the department and chose him to do my annual pap smears and follow me during my two pregnancies. The first delivery was difficult but he took care of everything and all turned out fine. With anyone else it may have not turned out well. The second delivery he came in on his day off to perform it. Amazing man. |
| by the time we saw this doctor, he was my husband’s only chance for survival. the dr. was direct and honest, very intelligent, and very serious while at the same time, he showed intense care and concern for what we were going through. he called me the day my husband died and asked me to come in so we could talk. I’m sure he was going to prepare me for the worst. |
| I found them to be exceptional for a number of reasons. Part of it was that we had the luxury of spending time to explore issues in depth - hourly sessions weekly or every other week were very helpful in taking the time to understand the problem. They were very empathetic and patient, but also challenged me to help me make sense of what was going on and develop tools I needed. |
| Yes, this doctor is the reason I’m writing to you now I was stabbed in my heart doctor did some little surgery right away then I was rushed into real surgeon surgery. Man I thank this doctor then after I was out of my crisis this doctor would do so many follow up with me to make sure I’m doing what I’m supposed to do to maintain the best man ever |
| My experience with this doctor is that she takes the time to go over my file with me in detail. She asks me questions about my lifestyle and is willing to work with me to help me achieve my goals. One of my goals was to get off of my blood pressure medication. She listened and agreed with me and I succeeded in being able to get off of them. |
| Dr. Girardi is, without doubt, a brilliant surgeon, with an excellent team. After his first reading of my son’s ascending aortic artery film he called him, at 10:30pm on a Wednesday night and said, "You should be dead, I’d like to schedule your surgery on Friday!" Through his good efforts, including a dissection and an abdominal aorta aneurysm my son would live another nine years, dying at age 42. |
| I have MS but I am still quite active compared to some, but it does still cause me problems. My GP was very understanding and sort of said there isnt a league table for problems and what is difficult for me isnt in competition with other peoples experience of MS and no matter how I suffer, it is still a big thing. It made me feel liek someone understood. |
| This doctor who takes care of my illness was introduced while I had been for a check up. He was someone who would not say medical terms which I had no knowledge of instead he would clearly give me in detail as to what what was my problems and make sure that I would not worry too much about my illness. He does not prescribe costly and heavy drugs. |
| At first I thought he seemed a like he wouldn’t be friendly etc....all business. But he became warmer as time went on. He was so smart and knew exactly what the problem was after asking me a bunch of questions. He knew all along from the x-rays, but wanted to make sure I guess. Great job explaining things to me. I wasn’t nervous at all about the surgery. |
| I found this doctor during a time in my life when I was at a crossroads whether to live or die. He was able to see me without an appointment and spent an hour with me, just talking and listening. He took care of me that day and for many years after that. He died a few years ago and I have not had a good doctor since. |
| Before he went in for this last surgery he called, wondering if he should fly back for a Doc G surgery. Alas, the pandemic was on and he, unvaxxed, was afraid to take the chance with flying. We discussed another surgeon, a west coast recommendation from Girardi, but he opted out of that, not wanting to jump through the hoops of changing docs at the last moment. |
| The main thing was, she was very thorough in her examination of me. She took her time, explaining what she was looking for. I also felt very comfortable by her gentle manner. I could tell from what she was doing, and saying, that she was quite knowledgeable about women’s health issues. She also had a "calmness," about her, which was quite reassuring to me, as a patient. |
| The doctor was my surgeon during a gall bladder emergency surgery. They were very compassionate, understanding and considerate towards my needs. They went above and beyond to explain so many details and never got frustrated with all of the questions I asked. They made me feel like my health and life were of top concern and not just another routine emergency surgery. |
| This good doctor would do routine blood work at least twice a year. If there was something that showed up in the blood test that he felt was more critical, he would repeat in 3-4 months time. He was kind and caring on each visit. I never felt rushed where he failed to listen to what I needed to tell him. |
| Thanks for this survey, my son been gone almost a year now and you are the first medical person I have discussed this with! Dr. Girardi is without doubt a fine person, a brilliant surgeon, an excellent manager of his team, his department. One of his residents told me, with awe, how fast he is as a surgeon! |
| By the next day, I was breathing much better and could take care of myself. I was still sick but the shallow breathing I had went away completely. He had saved my life. Later, he would do something similar for my daughter when she had a staph infection. He also probably saved her life. |
| He listens to me. He takes time to understand my concerns. He is ok with my checking his recommendations with my gf who is a pediatric anesthesiologist. Sometimes his orders include Talk to Elizabeth before deciding course of action. :-).. Empathy and taking the time to listen I think makes a huge difference. |
| JAN were all very friendly and helpful. I especially loved how Dr.JAN really took his time to explain my conditions with me as well as my treatment options. I had a great visit and the doctor’s demeanor has really put me at ease so I highly recommend this clinic. |
| He is very through and takes him time to check you out really well. The las time he scraped my foot for almost an hour taking a vey thick callus of my foot. Taking his time and getting debris and everything out to make it feel much better. |
| He is very kind. He explained my problem to me in a way that I and my son who was along for the visit that I can understand what is happening to me. Having dizzy spells is just terrible and I need to get this taken care of. |
| He was beginning his practice when I was a teenager and because of a car accident, I was thrown through front windshield headfirst and needed immediate surgery. He was one of the doctors who operated on me that day & the opthalmologist I saw over many years. |
| The most wonderful treatment. I well and truly admire the person that you are and the doctor in you is honorable. The brilliant treatment deserve immense thanks and appreciation. Sending over our most humble and heartfelt thanks for your immense care and comfort during the treatment. |
| He took the time to listen to me when I had my visit. I was not feeling well and he was gentle and patient with me. He didn’t judge me at all and worked to make me comfortable while he was figuring out his diagnosis. |
| In the end, this resulted in a diagnosis of multiple sclerosis - a relief after years of seeking care for a range of symptoms but never receiving an accurate diagnosis. His persistence and advocacy made a huge difference in my life as a result. |
| Well he treated me for a accident I had at work he did a surgery on my right hand he took well care of me while I was at the hospital and when I went for my check up after the surgery. |
| I MEET THIS DOCOTOR BY MY FRIEND I HAD A DISK PROBLEM IN NECK AFTER A SMALL SURGERY IT HAS BEEN CLEAR, SHE IS VERY FRIENDLY AND SO EXPERIENCED IN THIS TYPE OF PROBLEM AFTER I MEET HIM I WAS CURED |
| I had a large bump on my head for years. I finally went to dr lee at tufts. He had a good demeanor and listened. He evaluated me. I came back for a procedure to remove it. Then again for followup |
| My daughter was extremely sick and did not end up making it out of the hospital. During the period, the doctor was honest with me, but still empathetic and understanding of the tough times we were going through. |
| The doctor was very patient during the examination. He thoroughly explained the surgical procedure. He went above and beyond to remove cataracts from both eyes, and adjusted the sight in each eye. He was very warm and friendly. |
| He was aware of all alternate options for my treatment and was clearly up to date on what those options were. He was very interested in looking at life hollistically in addition to in a purely pharmaceutical sense |
| The doctor welcomed me happily. asked me about my day then offered a seat. The Doctor was so humble and listened to me with great keenness. He then promised to help me as much as he could. |
| I had a problem with my toenail (needed to have it removed) and was in really bad pain. Doctor treated me with care, asked for my input, gave advice and resolved the problem quickly and relatively painlessly. |
| I was requested for a two-hour observation in the emergency room after a fall. The doctor said that she need to confirm no bleeding in my head. She explained in detail and was knowledgeable in her field. |
| At the appointment, she was kind and compassionate. She had put all my imaging and chemistry studies together, studied them and came up with a plan to help me avoid more stones that included medications and diet. |
| Not sure if there is somewhere to state this later on, but my good doctor of 20+ years got sick 2 years ago and had to retire. Out of necessity I had to find another doctor. |
| When I handed her the stone I had passed, she was actually excited to see it! I knew that she was a nerd for her specialty. She truly loved the study of prevention of kidney stones. |
| This dr has a wonderful "bedside" manner. He’s my knee nerve ablation dr. Ive had nerve ablations on both knees multiple times.He’s not judgemental, he’s a great listener and he’s great at what he does. |
| He spoke with such concern, I felt some sort of guilty for my behavior. Since that day I never really cared about anything. We can rarely find such honest doctors now a days. |
| Was being screened for prostate cancer and the doctor was very kind and answered all of my questions. He was able to put me at ease while performing some rather invasive procedures. |
| This doctor took a lot of time with me. She listened carefully to my concerns. She did not dismiss my concerns as me being too paranoid as some doctors have done. |
| I’m also a firefighter lieutenant on injury retirement with exposure to 9/11. I have Wegner’sfrom that. So I have a lot of unique concerns and have done a lot of research. |
| Went to the doctor several times in the past year as part of an ongoing health problem. Procedure went well and doctor was great during all the follow-up visits. |
| I have visited an exceptionally good doctor. My experience to visit him is very nice. He is very caring to his patient. He is also very kind and empathetic. |
| I have migraine, and this doctor in question was the only one to solve my problem, today I have a quality of life like I never had before. |
| He was a God Send.He diagnosed me with NMO.When I met him I was in a wheelchair and could barely walk.Within a month he had me walking again. |
| I had a trimalleolar fracture of my right ankle. It was severe. He took care of it and I walk without a limp still over 30 years later. |
| They were actually listening, flexible with their treatment, and willing to learn about PREP which I asked for and asked genuine questions about my weight loss plan |
| I had a really bad injury and nearly died from a cut and this doctor repaired me over 4 surgeries and was just an amazing kind person |
| In the shift on duty about a relative of mine, the doctor explained thoroughly about the clinical detailing of the patient, with reception, clarifying all doubts. |
| When I had an episode of depression the doctor was kind and compassionate. He took time to listen to me and laid out steps for treatment. |
| I MEET THIS DOCTOR FOR SMALL SURGERY IN MY LEG, I HAD A GOOD OUTCOME, I FEEL SAFE WITH THIS DOCTOR DIFFERENT TO OTHER DOCTOR |
| They took a holistic approach to my issues - they understood that it would involve a combination of talk therapy, medication, and underlying medical issues. |
| This good doctor conducted an extremely delicate surgical operation to remove a kidney where a carcinogenic tumor had appeared that was endangering my life. |
| I was very pleased with my experience at Doctors Care. Doctor was very nice and helpful in explaining how to take the medicine prescribed. |
| I HAD A ONE TIME CARDIO ATTACT AT THAT TIME THE REPOSABLITY TO CARE ME IN EVERY SECOUNDS ITS ENCOURGAE TO SAY THESE |
| She welcomed me with pleasant smile , I have the great experience towards her. She cared me lot and communicate well |
| I made an appointment to see her and she ordered imaging and lab tests to be done prior to the appointment. |
| I am a kidney stone maker and was referred to this doctor because she specializes in prevention of kidney stones. |
| I had passed a stone before the appointment and was instructed to take it with me to my appointment. |
| He gave me a gastroscopy, without feeling pain and solved the problem only with the treatment of medicines. |
| I got full explanation what should I do to get this small (3mm) stone out of my body. |
| This doctor was a psychiatrist who I was referred to for treatment of on-going depression issues. |
| She reassured me that she would do everything possible to take care of me. |
| He asked some questions, and found out my diagnose very fast: kidney stone. |
| Very helpful for me during this pandemic time and given a best medication |
| he speak very friendly and he talk and care about my health |
| He pretty much ruined what was a rather beautiful, wonderful experience. |
| OK, that was quite emotional, good emotions. Thank You. |
| I had a strong pain on my low back. |
| I visited emergency room in my local hospital. |
| Doctor I had met was highly professional. |
| I believe he is exceptionally good doctor. |
| Yes he consult me calmly |
| Wonderful Experience with doctor |

# Appendix 3 Survey feedback

Question 35: (Optional) Would you like to comment on the survey? Anything that can be improved or your opinion or any feedback or anything you would like added or removed? Did you enjoy doing the survey? Was it difficult? Was it quick or did it take a long time? Is there anything else you would like to add?

There were 221 answers

| **Most common** **survey feedback** |
| --- |
| Good survey or 'good' (63 times) |
| Nice survey or 'nice' (16 times) |
| No or nothing (27 times) |
| Gather more information (9 times) |

| **Survey feedback: Other answers, sorted alphabetically** |
| --- |
| A good doctor is not condescending, and admits when they don't know something. They will team with your to address your health issues. |
| A very good study. |
| All are good. I like very much. Interesting survey. Beautiful experience. |
| All clear and easy to understand, thank you. |
| All clear, no problems. |
| All good - thank you. |
| Awesome survey. |
| Do you have any additional comments, questions, or concerns you would like to share? KU Employee Satisfaction Survey |
| Enjoyed |
| extremely well survey and i am very interesting that survey |
| For a period of 8 years, I asked different doctors about a spot on my thigh that just appeared one day and for those 8 years, I received the same response, "Looks good to me, no problem there, it's okay and if you are worried, just keep an eye on it." 8 years later I had class 2 melanoma surgery and wear an 8 inch scar on upper right thigh. At least 5 doctors did no more than a cursory glance and told me not to worry about it, that it was nothing. An excellent doctor listens to a patient's worries for we know our bodies better, first and that is why we seek medical help because we have been told our entire lives, ',if you see something funny, check it with your Dr, they are the experts. I do. Those experts are full of themselves too many times. |
| GOOD AND EASY |
| GOOD AND EASY |
| Good And Easy To Do |
| good care of human life. |
| Good hit good pay. |
| GOOD OPINION |
| GOOD SURVEY I VERY ENJOY |
| GOOD VERY LIKE |
| good work |
| GOOD, YES ENJOYED |
| GOOD, YES I ENJOYED |
| GREAT JOB |
| Health foundation care life. |
| healthy funditation care life |
| hospitals are businesses. I understand that. Just because a doctor has been doing it for a long time... doesn't mean they're a good doctor. There's so many factors. I'd rather have one that's experienced and isn't going to bs me. no sugar coating. |
| I appreciate the OBGYN's I've had in the past. I had normal births, with no complications. I also appreciate that the women OBGYN's gave me helpful ways to deal with menopause.  I've had good internal medicine doctors in the past. They took the time to listen to me, treated me like a human. These days, so often I feel the doctors are treating patients as how the insurance companies want. |
| I didn’t have any problems with the survey, either technically or in understanding the questions. Good job. It was well done. Thank you for the opportunity. |
| I enjoyed sharing my thoughts and insights about what makes for an exceptional doctor. |
| I enjoyed the survey and I'm sorry that I wrote so much and in so much detail about the doctor, but I wanted to explain how skilled and personable he is. |
| I have met 2 great doctors One was extremely intelligent the other really is through and cares strongly for each of is patients. |
| I like a survey |
| i like that and i am very interesting that survey. Extremely very well project |
| i like this survey. |
| I REALLY ENJOYED AND INTERESTED THE SURVEY. AND NO COMPLICATED . |
| I really liked doing this one. I haven't thought about the doctor recently, but I truly appreciated all he did for me. |
| I think it is good that a researcher cares enough to research what patients believe is an exceptionally good doctor. |
| I think people are lucky to find a good doctor, we tend to stick to a good doctor for life. |
| i think this was very good |
| I thought that the survey was fine. It did take slightly longer than I thought it would though. |
| I would have liked the choice of "very skilled" or "experienced" in what makes a great MD.   I did enjoy the study. It was not difficult. It took me longer because I really wanted to write more about the doctor and probably included too much! |
| I’m so grateful to have been involved in this fantastic survey |
| Interesting survey. It made me think about my reasons for staying with the doctor I now have. The survey was a reasonable-length, without excessive bubbles/choices. |
| Interesting survey. Not difficult. Took reasonably quick amount of time for length, easy to understand. |
| interesting. |
| IT VERY INTERESTING AND VERY KNOWLEDGEABLE QUESTIONS IT HAD |
| IT VERY KNOWLEDGEABLE AND IT VERY NICE |
| It was a bit long for the compensation. |
| It was a perfect survey for me because I got to give feedback with a expierence I had with a good doctor I finally got to tell my story which I appreciate you for this thank you have a blessed day. |
| It was a very satisfying survey, I enjoyed answering it and I thank you for that. It was good to relive some old memories that in the end worked out and made me happy. Simple and quick survey. |
| it was an intresting survey |
| It was clear enough, no errors seen. Maybe add a few small photos? It was about right time and length wise. |
| It was easy to recount and describe my experience with the exceptional doctor who treated me. She was a diamond in the rough, I had never before been to a doctor who acted like they genuinely cared. She was a good listener and allowed me to pour my heart out after my mom died. The only reason I stopped going to her was because she retired from family practice and began homeopathy and in-home care. Good luck with your study. Thanks for the opportunity to participate. |
| it was fun |
| it was fun to be answering |
| It was nice study and the things are highly relatable to me lot. |
| It was very repetitious with the same questions asked many times (although about a different person) and it got boring after awhile. I did want to mention the exceptional doctor took no credit for his skill. He owed everything to God that he did or said. In fact, he jogged everyday to the hospital from his home and stopped at a chapel to pray for guidance in his work for that day. He was very humble. |
| it would be great if all doctors were like the exceptional one that i know |
| its easy survey i loved it thanks for the survey i am happy. |
| its most useful survey |
| Its very useful survey |
| its very useful survey |
| its very useful survey |
| its very useful survey |
| its very useful survey and its better than my feedback |
| Like it survey |
| Ok Thank you. |
| Overall, I enjoyed participating in this survey. Thank you for the experience. |
| Research is very important to reflect on the health professionals who serve us. It didn't take long, all the aspects asked were important. |
| Survey was good, and I had no problems with it. Thank you! |
| Thank you for allowing me to participate in this important research. |
| Thank you for providing us a 24-hour time, that is why I completed the optional fields too, you've been kind to us and I want to be considerate too of your time and patience. The survey was enjoyable, and I wish it could help to improve things on the topic, because there is room to improve. It is a bit personal but, I'm willing to share if it can change things. I wish you the best for your career. <3 |
| Thank you, I thought all of the questions were well explained. I would perhaps add the option to describe additional physicians if there is more than one outstanding doctor. The time frame for the survey seemed ideal, just long enough to adequately describe the essential features of the experience. |
| Thanks for giving chance do to stuyd |
| thanks for the opportunity |
| Thanks! |
| The survey is all right. I enjoyed doing the survey. Thank you. |
| The survey was clear and concise. One thing I'd like to remark on, was that the exceptional doctor I had, got fed up, with all the red-tape she had to deal with, and retired early, which was a great loss to the medical community. |
| The survey was easy to complete. Thank you. |
| The survey was interesting. I enjoyed the survey. It was not difficult. It was pretty quick. My impression of doctors has really changed with covid.We moved and my long time doctor was in our old state. I had a zoom call with him my first few days of covid and he said I would be fine and just ride it out. The longer I rode it out the sicker I became. He had been my doctor for over 20 years and I felt that he did not care about me one bit.   I submitted with my mturk id as the timer was expiring, I will message you the code, my internet had issues today and was down for quite awhile which is why my timer was expiring. |
| There were know problems. This was a very interesting study, I liked it. Made me smile thinking about my doctor cause he always has a few jokes to tell. So far it has taken me 22 min. |
| This is very interesting survey. |
| This subject is good but recalling doctor or hospitals are not enjoyable, but we cannot survive without them. |
| This survey is very interesting. |
| This survey was usefull and very enjoy. |
| This survey was very usefull and realy enjoy. |
| This was a great survey and first of it's kind that I have taken. Thank you for putting it together. |
| This was a very good survey and addressed topic which I feel should get more attention and it is unique among surveys, I have taken over 14,000. Might be interesting to throw in a few questions about the worst doctor experience too! - thanks. |
| Time was appropriate, wasn't too long, pay was good. |
| To find the mode: Look for the largest frequency and the corresponding value is the modal value or modal class |
| useful study |
| very good |
| VERY GOOD .I LIKE TO PARTICIPATE |
| VERY GOOD SURVEY |
| VERY INTERESTING |
| very interesting physician doctor this study |
| very interesting. |
| Very Nice and good |
| very nice survey |
| yes , thanks for this survey |
| Yes its nice and not to much longer and its helps to know about the good doctors. |
| Yes its nice study and not to much longer and its helps to know about the doctor profile. |
| YES THIS SURVEY WAS VERY GOOD |

# Appendix 4 Regression analyses with mean of Exceptionally Good Doctor and Average Doctor Likert questions as dependent variable.

## Exceptionally Good Doctor

Source | SS df MS Number of obs = 481

-------------+---------------------------------- F(8, 472) = 23.61

Model | 43.0221951 8 5.37777439 Prob > F = 0.0000

Residual | 107.508692 472 .227772652 R-squared = 0.2858

-------------+---------------------------------- Adj R-squared = 0.2737

Total | 150.530887 480 .313606014 Root MSE = .47726

----------------------------------------------------------------------------------------------------------------

Exceptionally good doctor | Coefficient Std. err. t P>|t| [95% conf. interval]

-----------------------------------------------+----------------------------------------------------------------

The doctor does whatever is needed to help | .1022838 .0586812 1.74 0.082 -.013025 .2175925

The doctor listens to me willingly to the end | .4087488 .059405 6.88 0.000 .2920178 .5254799

At or above 55 | .2647735 .0537432 4.93 0.000 .159168 .370379

Degree | -.0220227 .0580394 -0.38 0.705 -.1360703 .0920249

Respondent is female | .0526525 .0523077 1.01 0.315 -.0501322 .1554371

Specialist | .107637 .0441339 2.44 0.015 .0209138 .1943602

Doctor is female | .079503 .0526982 1.51 0.132 -.0240491 .1830551

Patient for a long time | .1443673 .067531 2.14 0.033 .0116688 .2770658

_cons | 3.806906 .0690803 55.11 0.000 3.671163 3.942649

----------------------------------------------------------------------------------------------------------------

## Average Doctor

Source | SS df MS Number of obs = 481

-------------+---------------------------------- F(8, 472) = 7.93

Model | 24.8950797 8 3.11188497 Prob > F = 0.0000

Residual | 185.208537 472 .392390968 R-squared = 0.1185

-------------+---------------------------------- Adj R-squared = 0.1035

Total | 210.103617 480 .437715868 Root MSE = .62641

----------------------------------------------------------------------------------------------------------------

Average Doctor | Coefficient Std. err. t P>|t| [95% conf. interval]

-----------------------------------------------+----------------------------------------------------------------

The doctor does whatever is needed to help | -.1036575 .0770208 -1.35 0.179 -.2550035 .0476885

The doctor listens to me willingly to the end | -.259544 .0779708 -3.33 0.001 -.4127568 -.1063313

At or above 55 | -.0962709 .0705395 -1.36 0.173 -.2348812 .0423393

Degree | .1251799 .0761784 1.64 0.101 -.0245108 .2748707

Respondent is female | .1584538 .0686553 2.31 0.021 .023546 .2933616

Specialist | .0207453 .057927 0.36 0.720 -.0930814 .134572

Doctor is female | .0346411 .0691679 0.50 0.617 -.1012739 .1705562

Patient for a long time | .2127108 .0886363 2.40 0.017 .0385403 .3868814

_cons | 3.549528 .0906699 39.15 0.000 3.371361 3.727694

----------------------------------------------------------------------------------------------------------------

# Appendix 5 Likert question linear regression analyses for exceptionally good and average doctors

Table Appendix 5a: The Likert questions are ordered by descending t-value for “Doctor listens willingly to end”, showing how much the 154 respondents whose doctors listen to them differ in their response to the remainder of the respondents. **Exceptionally good doctor** Likert questions.

*These tables show the results of the 34 linear regressions with each model having one of the Likert questions as the dependent variable. Table 6a shows the 34 regressions with exceptionally good doctor Likert questions.*

*We have highlighted the cases where an explanatory variable has either a t-value >= +2.5 (green) or <= -2.5 (red). We also highlighted the Likert question ‘The doctor is popular’ in Table S1a in yellow to show that patients whose doctors listen to them willingly to the end don’t see their doctor as more popular than other exceptionally good doctors do.*

| **Question text**  **Exceptionally good doctor** | **R^2^** | **N** | **Mean** | **SD** | **Doctor listens willingly to end** | **Does whatever is needed** | **Respondent over 55 years** | **Respondent degree** | **Respondent female** | **Doctor is specialist** | **Doctor female** | **Long term patient** |
| --- | --- | --- | --- | --- | --- | --- | --- | --- | --- | --- | --- | --- |
|  |  |  | **Likert score** |  | **(p-value,  t-value)** | **(p-value,  t-value)** | **(p-value,  t-value)** | **(p-value,  t-value)** | **(p-value,  t-value)** | **(p-value,  t-value)** | **(p-value,  t-value)** | **(p-value,  t-value)** |
| The doctor has integrity | 0.28 | 505 | 4.19 | 0.73 | (0.0000, 7.34) | (0.0309, 2.17) | (0.0001, 3.82) | (0.4044, -0.83) | (0.5106, -0.66) | (0.0412, 2.05) | (0.2558, 1.14) | (0.0215, 2.31) |
| The doctor is personable | 0.22 | 507 | 4.23 | 0.67 | (0.0000, 6.94) | (0.3440, 0.95) | (0.0025, 3.04) | (0.5666, 0.57) | (0.0765, 1.78) | (0.3395, 0.96) | (0.7558, 0.31) | (0.0612, 1.88) |
| The doctor is caring | 0.24 | 505 | 4.31 | 0.7 | (0.0000, 6.77) | (0.4180, 0.81) | (0.0000, 4.22) | (0.6279, -0.49) | (0.4812, 0.71) | (0.2608, 1.13) | (0.1505, 1.44) | (0.4351, 0.78) |
| The doctor cares for patient | 0.26 | 505 | 4.23 | 0.7 | (0.0000, 6.73) | (0.0536, 1.94) | (0.0028, 3.00) | (0.1328, -1.51) | (0.2332, 1.19) | (0.0442, 2.02) | (0.9941, 0.01) | (0.0141, 2.46) |
| The doctor is in good mental shape | 0.25 | 499 | 4.25 | 0.69 | (0.0000, 6.71) | (0.0739, 1.79) | (0.0000, 4.30) | (0.5876, -0.54) | (0.3900, -0.86) | (0.1250, 1.54) | (0.1685, 1.38) | (0.1989, 1.29) |
| The doctor has patience | 0.26 | 505 | 4.26 | 0.7 | (0.0000, 6.56) | (0.0761, 1.78) | (0.0000, 4.34) | (0.6081, -0.51) | (0.6229, 0.49) | (0.0201, 2.33) | (0.0613, 1.88) | (0.4069, 0.83) |
| The doctor gives the patient the time needed | 0.28 | 505 | 4.22 | 0.68 | (0.0000, 6.53) | (0.1702, 1.37) | (0.0000, 4.58) | (0.1079, -1.61) | (0.0323, 2.15) | (0.1260, 1.53) | (0.9148, 0.11) | (0.0621, 1.87) |
| The doctor is very thorough in the patient's assessment | 0.27 | 506 | 4.2 | 0.71 | (0.0000, 6.48) | (0.0896, 1.70) | (0.0000, 5.10) | (0.7518, -0.32) | (0.8935, 0.13) | (0.0030, 2.98) | (0.2781, 1.09) | (0.1177, 1.57) |
| The doctor is accurate in diagnosing the issue/ problem | 0.23 | 505 | 4.2 | 0.72 | (0.0000, 6.41) | (0.1380, 1.49) | (0.0003, 3.66) | (0.5981, -0.53) | (0.7730, 0.29) | (0.0419, 2.04) | (0.1643, 1.39) | (0.8232, -0.22) |
| The doctor is knowledgeable | 0.24 | 506 | 4.33 | 0.67 | (0.0000, 6.41) | (0.0542, 1.93) | (0.0004, 3.60) | (0.8149, 0.23) | (0.1792, 1.35) | (0.0285, 2.20) | (0.7193, -0.36) | (0.2760, 1.09) |
| The doctor is honest | 0.21 | 506 | 4.34 | 0.66 | (0.0000, 6.11) | (0.2333, 1.19) | (0.0001, 3.85) | (0.5857, -0.55) | (0.8049, 0.25) | (0.8797, 0.15) | (0.3318, 0.97) | (0.2125, 1.25) |
| The doctor is good at communicating | 0.25 | 506 | 4.32 | 0.67 | (0.0000, 6.01) | (0.2395, 1.18) | (0.0000, 5.12) | (0.4460, -0.76) | (0.5782, 0.56) | (0.0959, 1.67) | (0.2315, 1.20) | (0.2534, 1.14) |
| The doctor is understanding and/ or shows empathy | 0.24 | 507 | 4.25 | 0.7 | (0.0000, 5.82) | (0.1398, 1.48) | (0.0002, 3.75) | (0.0947, -1.67) | (0.0754, 1.78) | (0.1204, 1.56) | (0.7450, 0.33) | (0.1052, 1.62) |
| The doctor is good at explaining things | 0.23 | 505 | 4.29 | 0.68 | (0.0000, 5.71) | (0.0427, 2.03) | (0.0001, 4.03) | (0.6569, -0.44) | (0.6403, 0.47) | (0.0661, 1.84) | (0.2666, 1.11) | (0.1727, 1.37) |
| The patient trusts the doctor | 0.24 | 507 | 4.24 | 0.71 | (0.0000, 5.71) | (0.0289, 2.19) | (0.0001, 3.99) | (0.5537, -0.59) | (0.1053, 1.62) | (0.2287, 1.21) | (0.3961, -0.85) | (0.2165, 1.24) |
| Connects with the patient on a personal level | 0.23 | 504 | 4.14 | 0.7 | (0.0000, 5.59) | (0.4461, 0.76) | (0.0000, 4.41) | (0.3566, -0.92) | (0.2510, 1.15) | (0.0002, 3.73) | (0.4687, 0.73) | (0.1373, 1.49) |
| The doctor is confident | 0.18 | 505 | 4.26 | 0.68 | (0.0000, 5.57) | (0.5721, 0.57) | (0.0021, 3.10) | (0.3949, -0.85) | (0.3759, 0.89) | (0.0122, 2.51) | (0.8262, 0.22) | (0.0575, 1.90) |
| The doctor is a very good observer | 0.21 | 506 | 4.24 | 0.7 | (0.0000, 5.49) | (0.5717, 0.57) | (0.0000, 4.91) | (0.6617, -0.44) | (0.3354, 0.96) | (0.0780, 1.77) | (0.4496, 0.76) | (0.5818, 0.55) |
| The doctor is organised | 0.13 | 502 | 4.16 | 0.71 | (0.0000, 5.30) | (0.7268, -0.35) | (0.0084, 2.65) | (0.7269, -0.35) | (0.7933, -0.26) | (0.0045, 2.85) | (0.4024, 0.84) | (0.0219, 2.30) |
| The doctor is open minded | 0.17 | 504 | 4.21 | 0.7 | (0.0000, 5.28) | (0.4492, 0.76) | (0.0010, 3.30) | (0.9074, -0.12) | (0.4675, 0.73) | (0.4258, 0.80) | (0.0424, 2.03) | (0.0099, 2.59) |
| He/She sees patient as a whole person not just a collection of symptoms | 0.21 | 504 | 4.18 | 0.75 | (0.0000, 5.14) | (0.0228, 2.29) | (0.0004, 3.56) | (0.3751, -0.89) | (0.6562, 0.45) | (0.1808, 1.34) | (0.4948, 0.68) | (0.0993, 1.65) |
| Listens well, rarely or never interrupts | 0.23 | 505 | 4.12 | 0.82 | (0.0000, 4.92) | (0.0421, 2.04) | (0.0000, 4.51) | (0.0858, -1.72) | (0.6409, 0.47) | (0.2558, 1.14) | (0.3515, 0.93) | (0.1760, 1.36) |
| The doctor is adaptable, i.e. can respond to the unexpected | 0.17 | 493 | 4.05 | 0.76 | (0.0000, 4.87) | (0.0507, 1.96) | (0.0081, 2.66) | (0.4159, -0.81) | (0.8140, -0.24) | (0.2278, 1.21) | (0.1504, 1.44) | (0.0285, 2.20) |
| Acknowledges patient's experience and knowledge | 0.17 | 504 | 4.14 | 0.71 | (0.0000, 4.41) | (0.0557, 1.92) | (0.0125, 2.51) | (0.2400, -1.18) | (0.1079, 1.61) | (0.3702, 0.90) | (0.9285, 0.09) | (0.0587, 1.89) |
| Good at following things up or addressing items from prior consultation | 0.22 | 503 | 4.16 | 0.7 | (0.0000, 4.11) | (0.0013, 3.24) | (0.0000, 4.22) | (0.8699, -0.16) | (0.1749, 1.36) | (0.0069, 2.71) | (0.0938, 1.68) | (0.2595, 1.13) |
| The doctor is courageous when making difficult decisions | 0.11 | 482 | 4.08 | 0.7 | (0.0001, 3.97) | (0.5870, 0.54) | (0.0266, 2.22) | (0.8948, 0.13) | (0.4330, 0.78) | (0.0473, 1.99) | (0.2856, 1.07) | (0.0152, 2.44) |
| The doctor is in an especially harmonious or cared for treatment room | 0.10 | 491 | 4.1 | 0.7 | (0.0002, 3.74) | (0.6757, 0.42) | (0.0036, 2.93) | (0.0812, 1.75) | (0.5416, 0.61) | (0.1498, 1.44) | (0.1003, 1.65) | (0.2089, 1.26) |
| The doctor is humble | 0.11 | 501 | 4.16 | 0.73 | (0.0003, 3.69) | (0.4561, 0.75) | (0.0106, 2.57) | (0.7172, 0.36) | (0.4116, 0.82) | (0.0250, 2.25) | (0.0128, 2.50) | (0.0858, 1.72) |
| The patient has no fear of the doctor and may see as a friend | 0.12 | 505 | 4.08 | 0.74 | (0.0009, 3.36) | (0.0022, 3.08) | (0.1725, 1.37) | (0.9737, -0.03) | (0.4001, 0.84) | (0.0757, 1.78) | (0.4903, 0.69) | (0.4787, 0.71) |
| The doctor avoids using medical terminology I don't understand | 0.11 | 502 | 4.02 | 0.87 | (0.0015, 3.19) | (0.1382, 1.49) | (0.0020, 3.10) | (0.9895, -0.01) | (0.9013, 0.12) | (0.2582, 1.13) | (0.0343, 2.12) | (0.2024, 1.28) |
| Determined to get past bureaucratic obstacles that affect treatment | 0.10 | 478 | 4.03 | 0.72 | (0.0117, 2.53) | (0.8689, 0.17) | (0.0012, 3.26) | (0.0755, 1.78) | (0.1390, 1.48) | (0.1003, 1.65) | (0.3610, 0.91) | (0.0037, 2.91) |
| The doctor is in good physical shape | 0.10 | 501 | 4.09 | 0.7 | (0.0338, 2.13) | (0.7147, 0.37) | (0.0008, 3.37) | (0.2845, 1.07) | (0.0535, 1.94) | (0.4297, 0.79) | (0.0325, 2.14) | (0.0247, 2.25) |
| The doctor is always on time | 0.05 | 499 | 4.08 | 0.72 | (0.0651, 1.85) | (0.7146, -0.37) | (0.0086, 2.64) | (0.5298, 0.63) | (0.5541, -0.59) | (0.0298, 2.18) | (0.0375, 2.09) | (0.0530, 1.94) |
| The doctor is popular (if you have seen the doctor with others) | 0.11 | 465 | 3.99 | 0.78 | (0.8253, 0.22) | (0.8011, -0.25) | (0.0000, 4.45) | (0.0594, 1.89) | (0.0143, 2.46) | (0.0015, 3.19) | (0.3930, 0.85) | (0.1884, 1.32) |

Green: t-values >= 2.5. Yellow: Highlighting the Likert question where patients whose doctors listened to them willingly to the end see the least difference to other exceptionally good doctors.

Table Appendix 5b: Regression analyses with Likert questions as dependent variable. **Average doctor** Likert variables

| **Question text Average doctor** | **R^2^** | **N** | **Mean** | **SD** | **Doctor listens willingly to end** | **Does whatever is needed** | **Over 55 years** | **Degree** | **Respondent female** | **Specialist** | **Doctor female** | **Long term patient** |
| --- | --- | --- | --- | --- | --- | --- | --- | --- | --- | --- | --- | --- |
|  |  |  | **Likert score** |  | **(p-value,  t-value)** | **(p-value,  t-value)** | **(p-value,  t-value)** | **(p-value,  t-value)** | **(p-value,  t-value)** | **(p-value,  t-value)** | **(p-value,  t-value)** | **(p-value,  t-value)** |
| The doctor is confident | 0.04 | 503 | 3.89 | 0.71 | (0.1113, 1.60) | (0.5945, -0.53) | (0.0283, 2.20) | (0.2467, 1.16) | (0.0407, 2.05) | (0.8071, 0.24) | (0.9015, -0.12) | (0.1969, 1.29) |
| The doctor is knowledgeable | 0.03 | 504 | 3.86 | 0.7 | (0.6541, 0.45) | (0.2018, -1.28) | (0.4065, 0.83) | (0.5908, 0.54) | (0.0029, 2.99) | (0.8580, -0.18) | (0.1220, -1.55) | (0.5048, 0.67) |
| The doctor is organised | 0.03 | 497 | 3.76 | 0.74 | (0.6880, 0.40) | (0.4472, -0.76) | (0.2827, -1.08) | (0.5809, 0.55) | (0.0122, 2.52) | (0.6440, 0.46) | (0.7475, -0.32) | (0.2566, 1.14) |
| The doctor is in good mental shape | 0.02 | 492 | 3.83 | 0.72 | (0.9651, -0.04) | (0.8358, -0.21) | (0.4432, 0.77) | (0.7717, -0.29) | (0.3846, 0.87) | (0.6313, 0.48) | (0.1917, 1.31) | (0.0369, 2.09) |
| The doctor is honest | 0.03 | 498 | 3.84 | 0.75 | (0.6914, -0.40) | (0.8652, -0.17) | (0.3431, -0.95) | (0.5468, 0.60) | (0.0315, 2.16) | (0.1340, 1.50) | (0.2422, -1.17) | (0.0127, 2.50) |
| The doctor has integrity | 0.02 | 499 | 3.72 | 0.78 | (0.2915, -1.06) | (0.9718, -0.04) | (0.9192, 0.10) | (0.7386, 0.33) | (0.0498, 1.97) | (0.9368, 0.08) | (0.6674, -0.43) | (0.0414, 2.04) |
| The doctor is in good physical shape | 0.09 | 497 | 3.67 | 0.75 | (0.2442, -1.17) | (0.2915, -1.06) | (0.0906, -1.70) | (0.1447, 1.46) | (0.0383, 2.08) | (0.8292, -0.22) | (0.1554, 1.42) | (0.0016, 3.18) |
| The doctor cares for patient | 0.05 | 503 | 3.66 | 0.77 | (0.0607, -1.88) | (0.6805, -0.41) | (0.3913, -0.86) | (0.0236, 2.27) | (0.2500, 1.15) | (0.8347, 0.21) | (0.9488, -0.06) | (0.1561, 1.42) |
| The doctor is accurate in diagnosing the issue/ problem | 0.06 | 503 | 3.69 | 0.76 | (0.0587, -1.89) | (0.6787, 0.41) | (0.0950, -1.67) | (0.3798, 0.88) | (0.0006, 3.47) | (0.9963, 0.00) | (0.9500, 0.06) | (0.1705, 1.37) |
| The doctor is courageous when making difficult decisions | 0.08 | 489 | 3.65 | 0.8 | (0.0503, -1.96) | (0.6198, -0.50) | (0.0530, -1.94) | (0.4774, 0.71) | (0.0556, 1.92) | (0.1282, 1.52) | (0.6111, 0.51) | (0.0045, 2.86) |
| The doctor is a very good observer | 0.09 | 501 | 3.63 | 0.82 | (0.0186, -2.36) | (0.1652, -1.39) | (0.2367, -1.18) | (0.0351, 2.11) | (0.0507, 1.96) | (0.8741, -0.16) | (0.9672, 0.04) | (0.0896, 1.70) |
| The doctor is adaptable, i.e. can respond to the unexpected | 0.06 | 487 | 3.63 | 0.8 | (0.0173, -2.39) | (0.7041, -0.38) | (0.1550, -1.42) | (0.5279, 0.63) | (0.0893, 1.70) | (0.9989, 0.00) | (0.7574, 0.31) | (0.0277, 2.21) |
| The doctor is caring | 0.07 | 504 | 3.69 | 0.79 | (0.0116, -2.53) | (0.2389, -1.18) | (0.9985, 0.00) | (0.1266, 1.53) | (0.0725, 1.80) | (0.4638, 0.73) | (0.3719, 0.89) | (0.2084, 1.26) |
| The doctor is good at explaining things | 0.10 | 505 | 3.7 | 0.82 | (0.0046, -2.85) | (0.1383, -1.48) | (0.0540, -1.93) | (0.3699, 0.90) | (0.0029, 2.99) | (0.8640, 0.17) | (0.9694, -0.04) | (0.3759, 0.89) |
| The doctor is good at communicating | 0.11 | 503 | 3.65 | 0.81 | (0.0041, -2.88) | (0.1001, -1.65) | (0.0620, -1.87) | (0.4775, 0.71) | (0.0097, 2.60) | (0.5726, 0.56) | (0.3769, -0.88) | (0.0063, 2.74) |
| Good at following things up or addressing items from prior consultation | 0.08 | 502 | 3.66 | 0.79 | (0.0033, -2.95) | (0.2266, -1.21) | (0.8452, -0.20) | (0.0465, 2.00) | (0.2891, 1.06) | (0.8645, 0.17) | (0.4139, 0.82) | (0.4643, 0.73) |
| The doctor is popular (if you have seen the doctor with others) | 0.12 | 470 | 3.63 | 0.78 | (0.0029, -3.00) | (0.0973, -1.66) | (0.0155, -2.43) | (0.0814, 1.75) | (0.0144, 2.46) | (0.6681, -0.43) | (0.6951, -0.39) | (0.1414, 1.47) |
| The doctor is very thorough in the patient's assessment | 0.09 | 503 | 3.61 | 0.82 | (0.0028, -3.00) | (0.2754, -1.09) | (0.1959, -1.30) | (0.1463, 1.46) | (0.0256, 2.24) | (0.7754, 0.29) | (0.7464, 0.32) | (0.0875, 1.71) |
| The doctor is personable | 0.12 | 503 | 3.63 | 0.81 | (0.0011, -3.29) | (0.2783, -1.09) | (0.1502, -1.44) | (0.0174, 2.39) | (0.0143, 2.46) | (0.2911, 1.06) | (0.7070, -0.38) | (0.0844, 1.73) |
| The patient trusts the doctor | 0.09 | 500 | 3.62 | 0.77 | (0.0010, -3.32) | (0.8399, -0.20) | (0.6227, 0.49) | (0.0228, 2.28) | (0.0196, 2.34) | (0.9603, -0.05) | (0.9701, 0.04) | (0.0138, 2.47) |
| The doctor avoids using medical terminology I don't understand | 0.11 | 500 | 3.52 | 0.9 | (0.0009, -3.34) | (0.1313, -1.51) | (0.7939, -0.26) | (0.3519, 0.93) | (0.0009, 3.33) | (0.6149, 0.50) | (0.3216, 0.99) | (0.1640, 1.39) |
| The doctor has patience | 0.11 | 503 | 3.61 | 0.83 | (0.0006, -3.47) | (0.1353, -1.50) | (0.0787, -1.76) | (0.3628, 0.91) | (0.0340, 2.13) | (0.5243, -0.64) | (0.6323, 0.48) | (0.1278, 1.53) |
| The doctor is in an especially harmonious or cared for treatment room | 0.13 | 492 | 3.61 | 0.82 | (0.0004, -3.55) | (0.2045, -1.27) | (0.0096, -2.60) | (0.6917, 0.40) | (0.0231, 2.28) | (0.5964, -0.53) | (0.4159, 0.81) | (0.0368, 2.09) |
| The doctor is open minded | 0.12 | 498 | 3.59 | 0.86 | (0.0004, -3.58) | (0.3460, -0.94) | (0.1288, -1.52) | (0.2082, 1.26) | (0.0092, 2.62) | (0.9426, 0.07) | (0.9571, -0.05) | (0.0053, 2.80) |
| The doctor is understanding and/ or shows empathy | 0.11 | 502 | 3.63 | 0.81 | (0.0003, -3.61) | (0.2508, -1.15) | (0.2405, -1.18) | (0.5247, 0.64) | (0.2508, 1.15) | (0.7478, 0.32) | (0.1759, 1.36) | (0.0403, 2.06) |
| The doctor gives the patient the time needed | 0.14 | 504 | 3.51 | 0.89 | (0.0003, -3.64) | (0.0838, -1.73) | (0.0269, -2.22) | (0.2163, 1.24) | (0.7339, 0.34) | (0.5858, 0.55) | (0.3271, 0.98) | (0.0476, 1.99) |
| Determined to get past bureaucratic obstacles that affect treatment | 0.15 | 487 | 3.59 | 0.89 | (0.0002, -3.71) | (0.0242, -2.26) | (0.0886, -1.71) | (0.2379, 1.18) | (0.0281, 2.20) | (0.3225, 0.99) | (0.5566, 0.59) | (0.0087, 2.64) |
| Acknowledges patient's experience and knowledge | 0.12 | 504 | 3.61 | 0.87 | (0.0002, -3.74) | (0.0613, -1.88) | (0.4545, -0.75) | (0.0953, 1.67) | (0.4214, 0.80) | (0.8370, 0.21) | (0.6084, 0.51) | (0.0342, 2.12) |
| Connects with the patient on a personal level | 0.15 | 502 | 3.5 | 0.86 | (0.0002, -3.76) | (0.1120, -1.59) | (0.1393, -1.48) | (0.0068, 2.72) | (0.4546, 0.75) | (0.2531, 1.14) | (0.6794, 0.41) | (0.0229, 2.28) |
| The doctor is humble | 0.17 | 499 | 3.53 | 0.92 | (0.0000, -4.17) | (0.0376, -2.09) | (0.0342, -2.12) | (0.2254, 1.21) | (0.1227, 1.55) | (0.8464, 0.19) | (0.5508, 0.60) | (0.0132, 2.49) |
| He/She sees patient as a whole person not just a collection of symptoms | 0.14 | 502 | 3.58 | 0.88 | (0.0000, -4.35) | (0.1271, -1.53) | (0.3922, -0.86) | (0.0503, 1.96) | (0.2102, 1.25) | (0.7110, 0.37) | (0.4166, 0.81) | (0.0285, 2.20) |
| The patient has no fear of the doctor and may see as a friend | 0.13 | 502 | 3.52 | 0.88 | (0.0000, -4.39) | (0.2933, -1.05) | (0.9673, -0.04) | (0.0407, 2.05) | (0.8527, 0.19) | (0.3789, 0.88) | (0.1838, 1.33) | (0.0418, 2.04) |
| Listens well, rarely or never interrupts | 0.12 | 505 | 3.52 | 0.89 | (0.0000, -4.49) | (0.5049, -0.67) | (0.1126, -1.59) | (0.2317, 1.20) | (0.0261, 2.23) | (0.9351, 0.08) | (0.7684, -0.29) | (0.0279, 2.21) |
| The doctor is always on time | 0.23 | 501 | 3.5 | 0.98 | (0.0000, -5.60) | (0.0387, -2.07) | (0.0246, -2.25) | (0.1497, 1.44) | (0.3080, 1.02) | (0.3544, 0.93) | (0.0479, 1.98) | (0.0123, 2.51) |

Green: t-values >= 2.5, red: t-values <= -2.5.

# Appendix 6 Factor analysis

We investigated the survey data using factor analysis^45^ of the correlation matrix of the analyzed variables to test the assumption that the Likert and other questions measure characteristics of exceptionally good doctors.

We found that the 34 Likert questions asking how much exceptional doctors fulfil 34 characteristics plus “I trust this doctor more than other doctors” and “The doctor listens to me willingly to the end” yield Eigenvalues of 20.9 and 1.2 for the first two factors, i.e. these questions overwhelmingly measure a single dominating underlying factor, presumably characteristics of an exceptional doctor.

Figure 6


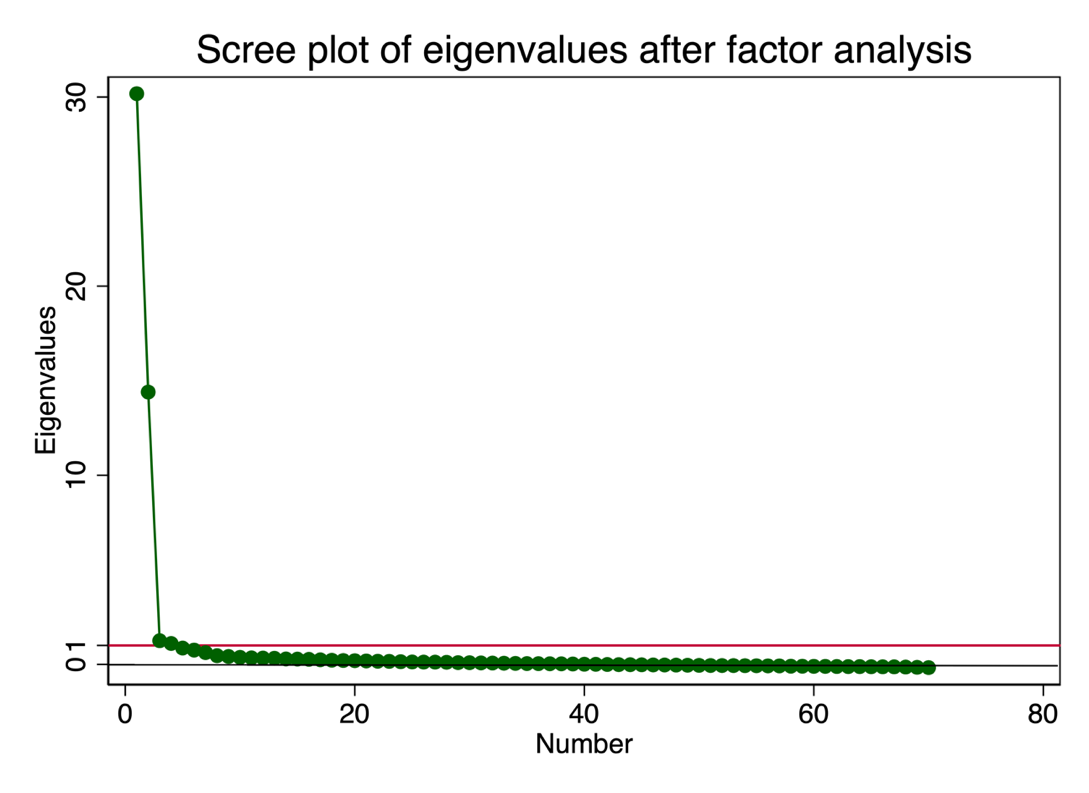


We tested this by expanding the factor analysis by adding the 34 identical Likert questions where the respondents were asked how much the average doctor fits the 34 questions. In this case factor 1 had an Eigenvalue of 30.2, factor 2 of 14.4 and factor 3 of 1.3. See the scree plot on Figure 6. After varimax rotation, factor 1 was 23.1, factor 2, 21.1 and factor 3, 0.78, showing that two major underlying factors were measured. Every Likert question for exceptionally good doctors loaded on factor 2, and every Likert question for average doctors loaded on factor 1. All but one loading (0.52) were high at between 0.60 and 0.85 for the Likert questions. The two underlying factors seem to be general doctor characteristics measured in Factor 1 and exceptionally good doctor characteristics measured in Factor 2. See Table 5 for details.

The question “I trust this doctor more than other doctors” has a small loading (0.35) for Factor 2 and the question “The doctor listens to me willingly to the end” has, after rotation, the only moderately substantial negative loading of all variables, -0.38 for Factor 1 and a positive loading of 0.46 for Factor 2. Therefore the quality of listening is a characteristic of exceptionally good doctors but seems to be negatively associated with the average doctor.

Table 5 Rotated factor loadings (pattern matrix) and unique variances

|  | **Exceptionally good  doctor** | | **Average  doctor** | |
| --- | --- | --- | --- | --- |
| **Question text** | **Factor 1** | **Factor 2** | **Factor 1** | **Factor 2** |
| The doctor cares for patient |  | 0.35 | 0.81 |  |
| The doctor listens to me willingly to the end | -0.38 | 0.46 | 0.85 |  |
| Good at following things up or addressing items from prior consultation |  | 0.86 | 0.83 |  |
| Listens well, rarely or never interrupts |  | 0.76 | 0.84 |  |
| Connects with the patient on a personal level |  | 0.82 | 0.83 |  |
| The patient has no fear of the doctor and may see as a friend |  | 0.73 | 0.79 |  |
| The patient trusts the doctor |  | 0.78 | 0.81 |  |
| He/She sees patient as a whole person not just a collection of symptoms |  | 0.71 | 0.83 |  |
| The doctor is very thorough in the patient's assessment |  | 0.82 | 0.84 |  |
| The doctor is a very good observer |  | 0.74 | 0.85 |  |
| The doctor gives the patient the time needed |  | 0.83 | 0.86 |  |
| The doctor is confident |  | 0.80 | 0.60 | 0.39 |
| The doctor is courageous when making difficult decisions |  | 0.85 | 0.82 |  |
| The doctor is good at communicating |  | 0.82 | 0.86 |  |
| The doctor is adaptable, i.e. can respond to the unexpected |  | 0.72 | 0.78 |  |
| The doctor is honest |  | 0.84 | 0.76 |  |
| The doctor is humble |  | 0.74 | 0.86 |  |
| The doctor has integrity |  | 0.82 | 0.77 |  |
| The doctor is open minded |  | 0.72 | 0.85 |  |
| The doctor is organised |  | 0.80 | 0.74 | 0.31 |
| The doctor is personable |  | 0.77 | 0.85 |  |
| Determined to get past bureaucratic obstacles that affect treatment |  | 0.77 | 0.86 |  |
| The doctor is understanding and/ or shows empathy |  | 0.81 | 0.86 |  |
| The doctor avoids using medical terminology I don't understand |  | 0.64 | 0.72 |  |
| The doctor is accurate in diagnosing the issue/ problem |  | 0.81 | 0.77 |  |
| The doctor is good at explaining things |  | 0.52 | 0.84 |  |
| The doctor is knowledgeable |  | 0.77 | 0.65 | 0.30 |
| The doctor is popular (if you have seen the doctor with others) |  | 0.80 | 0.78 |  |
| The doctor is in good physical shape |  | 0.81 | 0.72 |  |
| The doctor is in good mental shape | 0.35 | 0.54 | 0.64 | 0.32 |
| The doctor is in an especially harmonious or cared for treatment room | 0.32 | 0.61 | 0.81 |  |
| The doctor is always on time |  | 0.80 | 0.81 |  |
| The doctor has patience |  | 0.69 | 0.85 |  |
| The doctor is caring |  | 0.68 | 0.82 |  |
| Acknowledges patient's experience and knowledge |  | 0.81 |  |  |
| Good at following things up or addressing items from prior consultation |  | 0.80 |  |  |

# Appendix 7 – Survey questions
