# Supplementary material for: Characteristics of exceptionally good Doctors—A survey of public adults
Source: Heliyon. 2023 Jan 21;9(2):e13115. doi: 10.1016/j.heliyon.2023.e13115 (PMC9883187; doi:10.1016/j.heliyon.2023.e13115)
Supplement: Multimedia component 2 [file mmc2.pdf]

Supplementary File

Patients' survey of Exceptional Doctors

Q1 [Bond University Logo]

Q2 Consent form

What makes an exceptionally good doctor (physician)?

Dear Participant, My name is Christoph Schnelle and I am currently completing a PhD at Bond University at the Institute for Evidence-Based Healthcare under the supervision of Dr Mark Jones.

We are conducting a research investigation into the experience of medical doctors. I am specifically interested in finding out what is an experience of an exceptionally good doctor. It has ethical approval CS03416 from the Bond University Human Research Ethics Committee.

This anonymous survey will take between 15 and 20 minutes. Participation in this survey is completely voluntary. The information I/we obtain from you will be dealt with in a manner that ensures you remain anonymous. Your participation in this study will enhance work towards learning from exceptionally good doctors.

Participation in this study is completely voluntary and you may withdraw at any time without risking any negative consequences. The information I/we obtain from you will be dealt with in a manner that ensures you remain anonymous. Data will be stored in a secured location at Bond University for a period of 5 years after the end of this project as per 601.3/C150 of the Qld Government University Sector Retention and Disposal Schedule in accordance with the guidelines set out by the Bond University Human Research Ethics Committee.

It is anticipated that the data collected during this study will assist us in understanding what doctors consider or experience to be exceptionally good doctors. Your participation in this study will enhance work towards learning from exceptionally good doctors.

If you experience distress from participation in this research, please contact an organisation such as SANE Helpline on +61 1800 187 263.

Should you have any complaints concerning the manner in which this research is being conducted please make contact with –

Bond University Human Research Ethics Committee,
Bond University Office of Research Services.
Bond University, Gold Coast, 4229, Australia Tel: +61 7 5595 4194 Fax: +61 7 5595 1120 email: ethics@bond.edu.au

Many thanks,

Christoph and Mark

Q4 Do you consent to participate in this survey?

◯ Yes

◯ No

End of Block: Consent

Start of Block: ED Contact?

Q5 In your opinion, what are three to five traits, aspects or qualities that would make a doctor (physician) an exceptionally good doctor (physician)?

◯ 1 ____

◯ 2 ____

◯ 3 ____

◯ 4 ____

◯ 5 ____

Q6 Which of these groups do you fall into?
Please select one. If more than one is true, select the earlier answer.

◯ I have been treated in the past by an exceptionally good doctor

◯ I have met an exceptionally good doctor but was not treated by one

◯ I know of an exceptionally good doctor but have not met him/her

◯ None of the above

Q7 We will now ask you a series of questions about your experiences with an exceptionally good doctor. As you may have encountered more than one exceptionally doctor in your life, when answering this section, please only think about the MOST exceptional of these exceptionally good doctors.

End of Block: ED Contact?

Start of Block: Doctor 1 Part a

Q8 The most exceptionally good doctor (physician)

Q9 What is the **doctor's gender?** For the purpose of this survey we need to refer to the doctor as either him or her.

○ Male

○ Female

Q10 Do you have an idea how old the doctor was when you met them? It is fine to make an estimate.

○ Under 25 years old

○ 25-34 years old

○ 35-44 years old

○ 45-54 years old

○ 55-64 years old

○ 65+ years old

Q11 Do you know what type of doctor he/she is?

- ○ A primary care specialist (GP or Family Medicine / Doctor)
- ○ A medical specialist working mostly or exclusively in a hospital
- ○ A medical specialist working mostly or exclusively outside a hospital
- ○ A doctor in a hospital who is not a specialist
- ○ I met the doctor in a hospital and I think he/she is a specialist
- ○ I met the doctor in a hospital and I think he/she is NOT a specialist
- ○ I met the doctor outside a hospital and I think he/she is a specialist
- ○ I met the doctor outside a hospital and I think he/she is NOT a specialist
- ○ Other, could you give details?

____

Q12 (Optional) If it is a medical specialist (not primary care or GP), could you select the specialty from the list?

- ◯ Addiction medicine
- ◯ Anaesthetist
- ◯ Cardiologist
- ◯ Community child health
- ◯ Dermatologist
- ◯ Emergency physician
- ◯ Endocrinologist
- ◯ Gastroenterologist and hepatologist
- ◯ Geriatrician
- ◯ Gynaecological oncologist
- ◯ Haematologist
- ◯ Hospitalist or Internal Medicine
- ◯ Immunologist
- ◯ Immunologist and allergist
- ◯ Infectious diseases physician
- ◯ Intensive care physician
- ◯ Maternal–fetal medicine
- ◯ Medical administrator
- ◯ Oncologist
- ◯ Nephrologist
- ◯ Neurologist
- ◯ Neurosurgeon
- ◯ Obstetrician and gynaecologist
- ◯ Ophthalmologist
- ◯ Paediatrician

- ◯ Pain medicine physician
- ◯ Palliative medicine physician
- ◯ Pathologist
- ◯ Psychiatrist
- ◯ Public health physician
- ◯ Radiologist
- ◯ Rehabilitation physician
- ◯ Reproductive endocrinology and infertility
- ◯ Respiratory and sleep medicine physician
- ◯ Rheumatologist
- ◯ Sexual health physician
- ◯ Sport and exercise physician
- ◯ Surgeon
- ◯ Surgeon, cardio-thoracic
- ◯ Surgeon, general
- ◯ Surgeon, oral and maxillofacial
- ◯ Surgeon, orthopaedic
- ◯ Surgeon, otolaryngologist – head and neck
- ◯ Surgeon, paediatric
- ◯ Surgeon, plastic
- ◯ Surgeon, vascular
- ◯ Urologist
- ◯ I am not sure or I can't find the specialty

Page Break

Q13 How did you first come across this doctor? Tick **all** that apply.

- [ ] Discovered via an internet search
- [ ] Recommended to me by a health care professional
- [ ] Recommended by a friend or family member or acquaintance
- [ ] No recommendation, I found him or her myself
- [ ] The doctor treated a colleague of mine
- [ ] The doctor treated a family member (spouse, children, parents, siblings, close cousins, etc.)
- [ ] I worked for the doctor as an employee or the doctor was my superior
- [ ] The doctor worked for me
- [ ] The doctor was a colleague
- [ ] The doctor was my teacher
- [ ] The doctor was my student
- [ ] The doctor *is* a close or extended family member
- [ ] Other, please specify

____

Q14 Please describe the circumstances under which you first met this doctor. Please select one.

- ◯ I was a patient for a general health check-up
- ◯ I was a patient for one health event, like an operation or a particular illness
- ◯ I was a patient for multiple health events
- ◯ I was / am his or her patient for a long time
- ◯ Other, please specify ____

Q15 How many times did you visit **this** doctor in the last 12 months? Please select one.

- ◯ Not at all
- ◯ 1-2 times
- ◯ 3-5 times
- ◯ 6-10 times
- ◯ 11-20 times, i.e. on average 1-2 times a month
- ◯ 21-50 times
- ◯ 51 or more times, i.e. on average every week

End of Block: Doctor 1 Part a

Start of Block: Doctor 1 Part b

Q16 Would you like to tell us about your experience with this exceptionally good doctor in your own words? (Optional)
That would be very helpful because every individual experience can be very different and currently little is known about exceptionally good doctors.

____

____

____

____

____

Page Break

Q17 What made you think this doctor is exceptionally good?
Choose **all** that apply.

- [ ] It was an overall impression, there are multiple reasons
- [ ] I had an outstanding outcome, for example an unexpectedly successful operation or a recovery against the odds.
- [ ] Because of this doctor I am healthier than I would otherwise be
- [ ] Because of this doctor I am *much* healthier than I would otherwise be
- [ ] This doctor definitely or probably saved my life
- [ ] I trust this doctor more than other doctors
- [ ] I feel safe with this doctor, different to other doctors
- [ ] I know the doctor will do whatever is needed to help me or has done so
- [ ] The doctor treats financially poor patients at a discount or for free
- [ ] The doctor allows me to make my own decisions
- [ ] The doctor is ready to extend guidelines and prescribe medications beyond their original intended use ("off-label")
- [ ] The doctor empowered me in my healing or treatment process much more than I thought was possible
- [ ] The doctor listens to me willingly to the end
- [ ] Another reason, which is:

____

End of Block: Doctor 1 Part b

Start of Block: Doctor 1 Part c

Page Break

Display This Question:

If Q17 = I had an outstanding outcome, for example an unexpectedly successful operation or a recovery against the odds.

Or Q17 = Because of this doctor I am healthier than I would otherwise be

*Or Q17 = Because of this doctor I am *much* healthier than I would otherwise be*

Or Q17 = This doctor definitely or probably saved my life

Or Q17 = The doctor empowered me in my healing or treatment process much more than I thought was possible

Q18 Could you state your reasons as to why you said this earlier?
Choose **all** that apply.

- ▢ I had a diagnosis that other doctors missed
- ▢ I had a diagnosis that was difficult because my symptoms were obscure / hidden / unusual
- ▢ I had a diagnosis that transformed my life for the better
- ▢ I had a dangerous or difficult operation and it went well
- ▢ I was not expected to recover from a non-terminal illness but did
- ▢ I was not expected to recover from a terminal illness but did
- ▢ The doctor changed my medication with a big beneficial effect
- ▢ The doctor gave me a different treatment that worked very well
- ▢ The doctor removed medication or other treatments and I was much better
- ▢ Other, could you specify? ____

Page Break

Q19

Important - we ask this question about **the exceptionally good doctor** <u>you mentioned previously</u> here. In your evaluation, to what extent does the doctor have these traits? If you don't know or cannot be sure, **tick** 'Not sure'. If you know the doctor as a patient, then the patient in this question is *you*.

If you get an error message at the end it is usually because you haven't moved a pointer at all.

	Completely disagree	Disagree	Neither agree or disagree	Agree	Completely agree	Not sure
	1	2	3	4	5	
Cares for patient						
Acknowledges patient's experience and knowledge						
Good at following things up or addressing items from previous consultation						
Listens well, rarely or never interrupts						
Connects with the patient on a personal level						
The patient has no fear of the doctor and may see them as a friend						
The patient trusts the doctor						
The doctor sees the patient as a whole person, not just a collection of symptoms						
The doctor is very thorough in the patient's assessment						
The doctor is a very good observer						
The doctor gives the patient the time needed						

Page Break

Q20 In your evaluation, is/was the exceptionally good doctor:
If you don't know or cannot be sure, tick 'Not sure'.
If you get an error message at the end it is usually because you haven't moved a pointer at all.

	Completely disagree	Disagree	Neither agree nor disagree	Agree	Completely agree	Not sure
	1	2	3	4	5	
Confident						
Courageous when making difficult decisions						
Good at communicating						
Adaptable, i.e. can respond to the unexpected						
Honest						
Humble						
Has integrity						
Open minded						
Organised						
Personable						
Determined to get past bureaucratic obstacles that affect the treatment						
Understanding and/ or shows empathy						

Page Break

Q21 In your evaluation, is/was/does the exceptionally good doctor:
If you don't know or cannot be sure, tick 'Not sure'.
If you get an error message at the end it is usually because you haven't moved a pointer at all.

	Completely disagree	Disagree	Neither agree nor disagree	Agree	Completely agree	Not sure
	1	2	3	4	5	
Avoids using medical terminology I don't understand						
Accurate in diagnosing the issue/ problem						
Good at explaining things						
Knowledgeable						
Popular (if you have seen the doctor with others)						
In good physical shape						
In good mental shape						
In an especially harmonious or cared for treatment room						
Always on time						
Has patience						
Is caring						

Page Break

Q22
Important - we ask this question about **<u>the average doctor</u>** here. In your evaluation, to what extent does the **average** doctor have these traits? If you don't know or cannot be sure, tick 'Not sure'. If you have ever been to a doctor who you consider to be an average doctor, then the patient in this question is *you*.
If you get an error message at the end it is usually because you haven't moved a pointer at all.

	Completely disagree	Disagree	Neither agree or disagree	Agree	Completely agree	Not sure
	1	2	3	4	5	
The **average** doctor cares for the patient						
Acknowledges patient's experience and knowledge						
Good at following things up or addressing items from previous consultation						
The **average** doctor listens well, rarely or never interrupts						
Connects with the patient on a personal level						
The patient has no fear of the **average** doctor and may see them as a friend						
The patient trusts the **average** doctor						
The **average** doctor sees the patient as a whole person, not just a collection of symptoms						
The **average** doctor is very thorough in the patient's assessment						
The **average** doctor is a very good observer						
The **average** doctor gives the patient the time needed						

Page Break

Q23

More than half way through.In your evaluation, is/was the **average** doctor:

If you don't know or cannot be sure, tick 'Not sure'.

If you get an error message at the end it is usually because you haven't moved a pointer at all.

	Completely disagree	Disagree	Neither agree nor disagree	Agree	Completely agree	Not sure
	1	2	3	4	5	
The **average** doctor is confident						
Courageous when making difficult decisions						
The **average** doctor is good at communicating						
Adaptable, i.e. can respond to the unexpected						
Honest						
Humble						
Has integrity						
Open minded						
The **average** doctor is organised						
Personable						
Determined to get past bureaucratic obstacles that affect the treatment						
Understanding and/ or shows empathy						

Page Break

Q24 In your evaluation, is/was/does the **average** doctor:
If you don't know or cannot be sure, tick 'Not sure'.
If you get an error message at the end it is usually because you haven't moved a pointer at all.

	Completely disagree	Disagree	Neither agree nor disagree	Agree	Completely agree	Not sure
	1	2	3	4	5	
Avoids using medical terminology I don't understand						
Accurate in diagnosing the issue/ problem						
Good at explaining things						
The **average** doctor is knowledgeable						
Popular (if you have seen the **average** doctor with others)						
The **average** doctor is in good physical shape						
In good mental shape						
In an especially harmonious or cared for treatment room						
Always on time						
The **average** doctor has patience						
Is caring						

End of Block: Doctor 1 Part c

Start of Block: Survey End

Q25 Mostly done

Q27 How many medical doctors (physicians) do you estimate you have met in your life?

▼ 1-5 ... 101 or more

Display This Question:

If Q6 = I have been treated in the past by an exceptionally good doctor

Or Q6 = I have met an exceptionally good doctor but was not treated by one

Q28 How many **exceptionally good doctors** have you met in your life?

▼ 1 ... 5 or more

Q29 In your opinion, what is needed for a doctor to be an **exceptionally good doctor**?

- ◯ It is enough for them to be outstanding at a single item, for example: a surgeon who is like all others except they are outstandingly skilled at one type of surgery, or a doctor who is bad-mannered but has outstanding diagnostic abilities, or a doctor who is outstandingly reassuring but otherwise normal, or a doctor who is particularly skilled in dealing with bureaucracy if their clients need to get compensation or government help.
- ◯ They need to be outstanding in at least two areas to be considered an exceptionally good doctor.
- ◯ For a surgeon it is enough if they are exceptionally good at one or more types of surgery, other doctors need to be exceptional in multiple areas.
- ◯ They need to be **outstanding** in everything to be considered an exceptionally good doctor
- ◯ They need to be **above average** in everything to be considered an exceptionally good doctor
- ◯ Other, please specify ____

Display This Question: [Q30]

If Q19 [Cares for patient] >= 4.5

Or Q19 [Acknowledges patient's experience and knowledge] > 4.5

Or Q19 [Good at following things up or addressing items from previous consultation] >= 4.5

Or Q19 [Listens well, rarely or never interrupts] >= 4.5

Or Q19 [Connects with the patient on a personal level] >= 4.5

Or Q19 [The patient has no fear of the doctor and may see them as a friend] >= 4.5

Or Q19 [The patient trusts the doctor] >= 4.5

Or Q19 [The doctor sees the patient as a whole person, not just a collection of symptoms] >= 4.5

Or Q19 [The doctor is very thorough in the patient's assessment] >= 4.5

Or Q19 [The doctor is a very good observer] >= 4.5

Or Q19 [The doctor gives the patient the time needed] >= 4.5

Or Q20 [Confident] >= 4.5

Or Q20 [Courageous when making difficult decisions] >= 4.5

Or Q20 [Good at communicating] >= 4.5

Or Q20 [Adaptable, i.e. can respond to the unexpected] >= 4.5

Or Q20 [Honest] >= 4.5

Or Q20 [Humble] >= 4.5

Or Q20 [Has integrity] >= 4.5

Or Q20 [Open minded] >= 4.5

Or Q20 [Organised] >= 4.5

Or Q20 [Personable] >= 4.5

Or Q20 [Determined to get past bureaucratic obstacles that affect the treatment] >= 4.5

Or Q20 [Understanding and/ or shows empathy] >= 4.5

Or Q21 [Avoids using medical terminology I don't understand] >= 4.5

Or Q21 [Accurate in diagnosing the issue/ problem] >= 4.5

Or Q21 [Good at explaining things] >= 4.5

Or Q21 [Knowledgeable] >= 4.5

Or Q21 [Popular (if you have seen the doctor with others)] >= 4.5

Or Q21 [In good physical shape] >= 4.5

Or Q21 [In good mental shape] >= 4.5

Or Q21 [In an especially harmonious or cared for treatment room] >= 4.5

Or Q21 [Always on time] >= 4.5

Or Q21 [Has patience] >= 4.5

Or Q21 [Is caring] >= 4.5

Q30 Which 3 of these items are most important for an **exceptionally good** doctor to have? Could you choose 3, with the most important on top.
If only 1, 2 or 3 items are displayed, move these into the box.
[Items are displayed if any item of Q19, Q20, or Q21 was scored at 4.5/5 or higher. The respondents indicate which of these are their top 3 choices]

Q31 How many times did you visit a doctor or went to hospital in the last 12 months?

- ○ Not at all
- ○ 1-2 times
- ○ 3-5 times
- ○ 6-10 times
- ○ 11-20 times, i.e. on average 1-2 times a month
- ○ 21-50 times
- ○ 51 or more times, i.e. on average every week

Q32 Could you tell us **your** age?

- ○ Under 18
- ○ 18-24 years old
- ○ 25-34 years old
- ○ 35-44 years old
- ○ 45-54 years old
- ○ 55-64 years old
- ○ 65+ years old

Q33 How do you describe yourself?

- ○ Male
- ○ Female
- ○ Non-binary / third gender
- ○ Prefer to self-describe ____
- ○ Prefer not to say

Q34 What is the highest level of education you have completed?

- ○ No schooling completed
- ○ Nursery school
- ○ Grades 1 through 11
- ○ 12th grade—no diploma
- ○ Regular high school diploma
- ○ GED or alternative credential (high school diploma equivalent)
- ○ Some college (university) credit, but less than 1 year of college
- ○ 1 or more years of college (university) credit, no degree
- ○ Associates degree (for example: AA, AS)
- ○ Bachelor's degree (for example: BA. BS)
- ○ Master's degree (for example: MA, MS, MEng, MEd, MSW, MBA)
- ○ Professional degree beyond bachelor's degree (for example: MD, DDS, DVM, LLB, JD)
- ○ Doctorate degree (for example, PhD, EdD)

Q35 (Optional) Would you like to comment on the survey? Anything that can be improved or your opinion or any feedback or anything you would like added or removed? Did you enjoy

doing the survey? Was it difficult? Was it quick or did it take a long time? Is there anything else you would like to add?

____

____

____

____

____

End of Block: Survey End
